# The RNA-binding protein RNP1A is essential and interacts with contractility kit proteins to facilitate cell mechanics

**DOI:** 10.1242/jcs.264128

**Published:** 2026-01-19

**Authors:** Yinan Liu, Mark Allan Co Jacob, Jessica Leng, Ly T. S. Nguyen, Alanoud Alotaibi, Douglas N. Robinson

**Affiliations:** ^1^ Department of Cell Biology, Johns Hopkins School of Medicine, Baltimore, MD 21205, USA; ^2^ Department of Physiology, Pharmacology, and Therapeutics, Johns Hopkins School of Medicine, Baltimore, MD 21205, USA

**Keywords:** Cell mechanics, Contractility kits, Cytokinesis, Macropinocytosis, RNA-binding protein

## Abstract

Cell shape regulation is important for many biological processes. Some cell shape-regulating proteins harbor mechanoresponsive properties that enable them to sense and respond to mechanical cues. In *Dictyostelium discoideum*, mechanoresponsive network proteins formed by proteins such as myosin II, cortexillin I and IQGAP1 assemble in the cytoplasm into macromolecular complexes, which we term contractility kits (CKs). In our previous studies, we identified the RNA-binding protein RNP1A as a genetic interactor with the cytoskeletal machinery of the cell and as a biochemical interactor of cortexillin I, using *in vivo* fluorescence cross-correlation spectroscopy. In this study, we show that *Dictyostelium rnp1A* knockdown cells have reduced cell proliferation, reduced adhesion, defective cytokinesis, and a gene expression profile that indicates *rnp1A* knockdown cells shift away from the vegetative growth state. Some of the transcripts RNP1A binds encode proteins involved in macropinocytosis, a crucial cell shape change process. Loss of other CK proteins leads to macropinocytotic defects characterized by reduced macropinocytotic crown size. RNP1A interacts with IQGAP1, leading to crosstalk during macropinocytosis. Overall, RNP1A binds transcripts and contributes to cell mechanics and cell shape change processes through interactions with CK proteins.

## Teaser

**Highlighted Article:** The mechanoresponsive machinery of the cell resides in the cytoplasm in contractility kits (CKs). Using *Dictyostelium* cells, we show that the CK protein RNP1A contributes to cell mechanics, cell shape changes and the vegetative state cell.

## INTRODUCTION

Cell shape control is crucial to many biological processes, including proliferation, differentiation, cell migration, endocytosis and exocytosis. Cell shape dynamics rely on cytoskeletal polymers, motors, adhesion factors, signaling proteins and membrane channels, which mediate responses to external stimuli (Jiang and Sun, 2013; Kaverina and Straube, 2011; McBeath et al., 2004; Morlot et al., 2012; Pollard and Cooper, 2009; Rape et al., 2011). Some proteins, termed mechanoresponsive proteins, sense and redistribute in response to mechanical stress, modulating local architecture and triggering signaling pathways (Kasza and Zallen, 2011; Luo et al., 2013; McWhorter et al., 2013; Ondeck et al., 2019; Schiffhauer et al., 2016). In *Dictyostelium discoideum*, these mechanoresponsive proteins include myosin II, cortexillin I and II, and IQGAP1 and IQGAP2 (Kee et al., 2012; Luo et al., 2013; Ren et al., 2009). In our previous work, we found that IQGAP1 and IQGAP2 play opposing roles in regulating cellular mechanoresponsiveness (Kee et al., 2012; Plaza-Rodriguez et al., 2022). Specifically, loss of IQGAP2 alone abolishes the mechanoresponsive phenotype, whereas loss of IQGAP1 does not. Interestingly, simultaneous removal of both IQGAP1 and IQGAP2 restores this response, indicating that IQGAP2 counteracts the negative regulatory effects of IQGAP1. Although these proteins function at the cell cortex, we found that they assemble into macromolecular complexes, termed contractility kits (CKs), in the cytoplasm independently of actin filaments (Kothari et al., 2019b). By organizing into CKs, the proteins diffuse together, which likely helps them coordinate their response to signaling and mechanical inputs, thereby allowing the cell to reconstruct the cortex as needed on a seconds-to-minutes timescale (Dolgitzer et al., 2025; Kothari et al., 2019b; Nguyen and Robinson, 2022; Plaza-Rodriguez et al., 2022). Although the CK concept is still being fully defined, CKs are dynamic heterogeneous macromolecular assemblies that include ∼10 or more proteins per complex (Plaza-Rodriguez et al., 2022). Through this heterogeneity, the CK complexes can be more or less mechanoresponsive, which allows the IQGAP1 and IQGAP2 proteins to provide setpoint control, establishing the level of mechanosensitivity (Kee et al., 2012; Plaza-Rodriguez et al., 2022). As an example of setpoint control, cytokinetic cells are significantly more mechanoresponsive than interphase cells, which reflects how the cell cortex shifts its machinery to ensure robust shape change (Kothari et al., 2019a,b; Luo et al., 2013; Ren et al., 2009).

To highlight the complexity of CKs and their integration into many cellular processes, we previously genetically identified the *D. discoideum* RNA-binding protein RNP1A (DDB ID: DDB_G0284167), as a suppressor of nocodazole when overexpressed, as a component of the CK system, and as a strong biochemical interactor of cortexillin I with an apparent *in vivo K*_D_ of 0.33 μM (Kothari et al., 2019b; Ngo et al., 2016; Zhou et al., 2010). A related RNA-binding protein, RNP1B (DDB ID: DDB_G0285361), was also identified as an interactor of cortexillin I in the proteomic study that led to the CK concept (Kothari et al., 2019b). These findings indicate a functional relationship between RNA-binding proteins (RBPs) and CK proteins.

RBPs regulate chromatin remodeling and RNA transcription, export, modification, localization, stability and translation. Their functions vary depending on binding partners and cellular stresses (Balcerak et al., 2019; Kilchert et al., 2020; Kishore et al., 2010). In *D. discoideum*, RBPs like Puf118 facilitate localization of RNA transcripts that encode proteins that are required for chemotaxis and that localize to the chemotaxing front (Hotz and Nelson, 2017). In other organisms, RBPs localize and translate β-actin mRNA at cell migratory fronts (Dermit et al., 2020; Zhang et al., 2001).

Here, we explore the role of RNP1A role in cell shape regulation and its interactions with CK proteins. The *rnp1A* gene is an essential gene, so we leveraged the long hairpin approach to knock down its expression. The *rnp1A* knockdown cells exhibited slower cell growth, decreased cell adhesion and cytokinetic defects. RNP1A interacts with IQGAP1, is slightly mechanoresponsive and contributes to cortical tension. Furthermore, gene expression profiling revealed that *rnp1A* knockdown downregulates pathways such as translation, which are essential for *D. discoideum* vegetative growth, while upregulating genes attributed to development. Using CLIP-Seq, we identified transcripts to which RNP1A binds; one of these transcripts encodes the dynamin-like protein DlpA, which is involved in macropinocytosis. DlpA is part of the dynamin-like protein family, which is involved in vesicle formation, membrane dynamics, and overall organelle division (Williams and Kim, 2014). Consistent with this, *rnp1A* knockdown and *dlpA*-null cells exhibited macropinocytosis defects. Furthermore, the CK proteins IQGAP1, cortexillin I and myosin II contributed to macropinosome size and dynamics. Overall, RNP1A interacts with CK proteins and transcripts to support cell shape change processes, including mechanoresponsiveness, cytokinesis and macropinocytosis.

## RESULTS

### RNP1A is important for cell growth, adhesion and cytokinesis

To investigate RNP1A function, we attempted to generate *D. discoideum rnp1A-*knockout and knockdown cell lines. We utilized CRISPR with two guide RNAs that only produced an in-frame deletion of three nucleotides (37–39), which was insufficient to knockout *rnp1A* (Sekine et al., 2018). A *Klebsiella aerogenes*-SorMC approach, designed for mutants with compromised macropinocytosis, also failed after screening 176 clones (Paschke et al., 2018). We then used a long hairpin RNAi approach to knock down *rnp1A*, acquiring *rnp1A* knockdown cells using a stringent drug regimen. These results suggest that *rnp1A* is essential for cell survival.

**Fig. 1.**
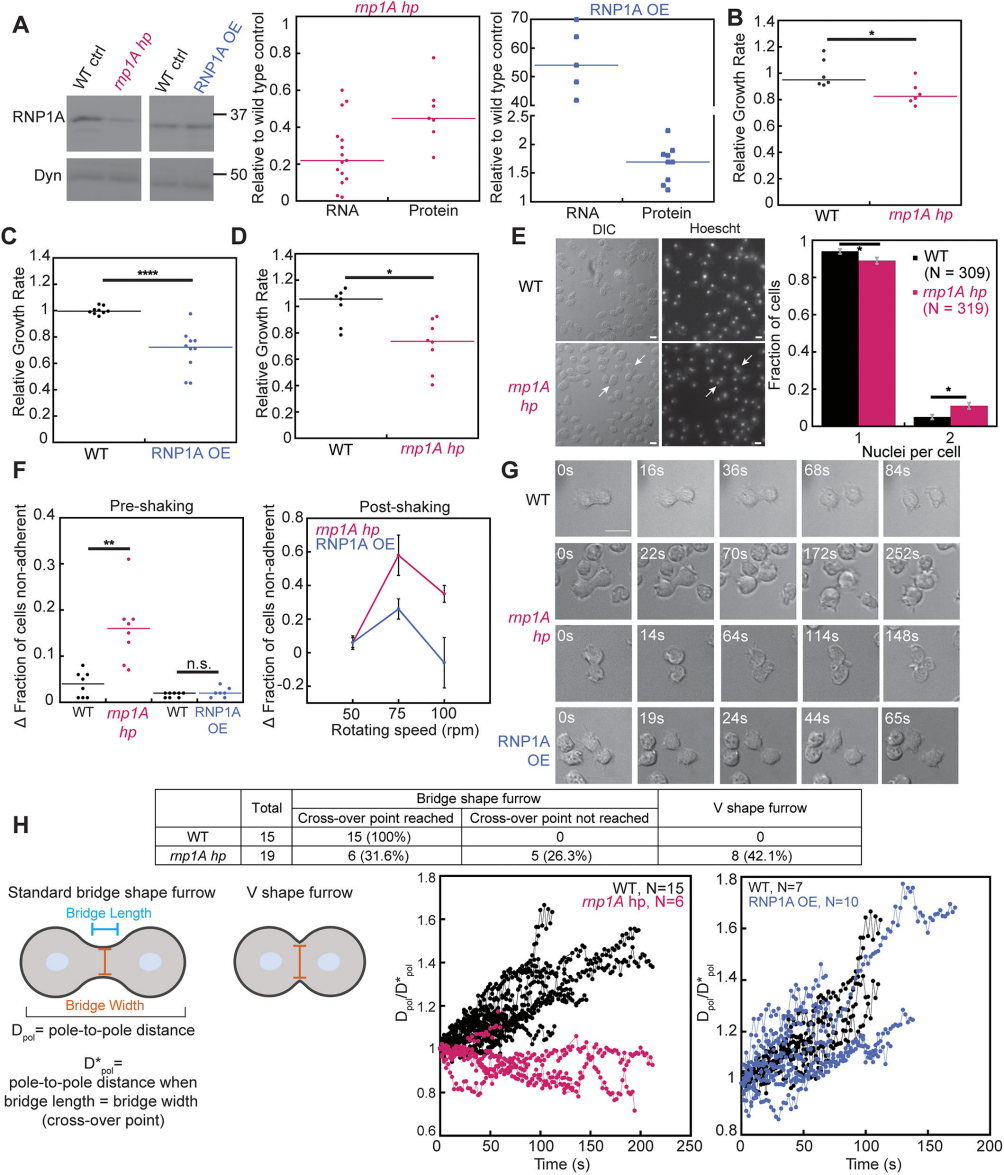
**RNP1A is important for cell growth, adhesion and cytokinesis.** (A) *rnp1A* knockdown and overexpression (OE) levels were quantified by qRT-PCR and western analysis. Dynacortin is a loading control. Original blots are provided in Fig. S6. Western blot data were analyzed by comparing to the total protein amount extracted from Coomassie gel. Each dot represents result from a single qRT-PCR run or a western blot. For *rnp1A* knockdown cells, qRT-PCR data were pooled from results from four different biological replicates and western blot data from two biological replicates. *P*-values are 0.0028 and 0.056 for the RNA and protein levels, respectively. For RNP1A overexpressing cells, both qRT-PCR data and western blot data are pooled from results from two biological replicates. *P*-values are 0.076 and 0.044 for the RNA and protein levels, respectively. Statistical analysis was performed using a Wilcoxon–Mann–Whitney test against wild-type controls. (B–D) Growth rates quantified from (B) a suspension culture of *rnp1A* knockdown cells, (C) a suspension culture of overexpressing cells, and (D) *rnp1A* knockdown cells grown on substrate were normalized to that of wild-type control. Each dot represents relative growth rate quantified from a single flask or a single well. Each data set is normalized to the wild-type control mean, but median bars are provided since the datasets are not always normally distributed. (E) Wild-type control and *rnp1A* knockdown cells were grown on surfaces and fixed with paraformaldehyde, and nuclei were stained with Hoechst 33342. Arrows point to example cells containing two nuclei. Comparison of proportions test was performed to determine statistical difference. Data were pooled from 309 wild-type control and 319 *rnp1A* knockdown cells. Comparison proportions analysis yields the following standard error values: 0.013 and 0.017 for WT and *rnp1A* hp, respectively. Gray error bars show s.e.m. Scale bar: 10 µm. (F) To assess adhesion of *rnp1A* knockdown and RNP1A-overexpressing cells, pre-shaking fraction of non-adherent cells was calculated as cells suspended in medium over total number of cells (left). Each dot represents the fraction calculated from a single well. Post-shaking fraction of non-adherent cells represents the incremental fraction of non-adherent cells compared to control (right). Error bars, s.e.m. Data were pooled from three independent experiments. Note that at higher rotation speeds, the fraction of adherent cells declines as the cells then get spun against the walls of the well. (G) DIC images of wild-type control, *rnp1A* knockdown and RNP1A-overexpressing cells during the progression of cytokinesis. Scale bar: 10 µm. (H) Number of cells exhibiting bridge-shaped or V-shaped cleavage furrows were counted for wild-type control and *rnp1A* knockdown cells. Among cells that exhibited bridge-shaped cleavage furrow, number of cells were counted based on if they reached cross-over point (when bridge diameter equals bridge length). Pole-to-pole distances were quantified at and after cells reached cross-over point and normalized to pole-to-pole distance at the cross-over point. For *rnp1A* knockdown, data are pooled from 15 wild-type control and 6 *rnp1A* knockdown cells. For RNP1A overexpression, data are pooled from 7 wild-type control and 10 RNP1A*-*overexpressing cells. *P≤0.05; **P≤0.01; ****P≤0.0001; n.s., not significant (Kruskal–Wallis followed by Wilcoxon–Mann–Whitney test). WT, wild type.

In *rnp1A* knockdown cells, transcript and protein levels were reduced by 78% and 52%, respectively, compared to that in wild type (Fig. 1A). Conversely, RNP1A-overexpressing (RNP1A-OE) cells showed a 54-fold increased transcript levels and 1.7-fold increased protein levels (Fig. 1A). Note that we have often seen limited correlation of protein expression with transcript levels, especially for overexpression of a gene using the traditional high-copy plasmid approach. Both knockdown and RNP1A-OE cells exhibited slower growth in suspension culture (Fig. 1B,C) with normalized growth rates of 0.83 (median) and 0.72 (median), respectively, relative to controls. Reduced growth rates might stem from impaired cell cycle progression, energy state, adhesion and/or cytokinesis fidelity (DeBerardinis et al., 2008; Kanada et al., 2005; Robinson and Spudich, 2000).

Cell growth on substrate revealed a slightly more severe defect in *rnp1A* knockdown cells with a normalized growth rate of 0.74 (median) relative to control (Fig. 1D), suggesting an adhesion defect. These cells also showed increased binucleation (11% versus 5% in wild type), indicating a mild cytokinesis defect (Fig. 1E). Please note that these phenotypes are associated with knockdown and are unlikely to be as severe as what would be seen in a knockout cell line, if such a line were attainable.

Adhesion assays confirmed *rnp1A* knockdown cells had impaired substrate adhesion with a higher fraction of non-adherent cells even before shaking after allowing cells to adhere for an hour (Fig. 1F). Upon shaking at 75 rpm and 100 rpm, non-adherence significantly increased in knockdown cells, whereas RNP1A-OE cells showed only a mild defect at 75 rpm, relative to their control. The reduced fraction of detached cells at 100 rpm compared to 75 rpm is due to centrifugal force driving the cells to stick to the walls of the flask upon release from the surface, a phenomenon we have observed in prior studies (Octtaviani et al., 2006). Adhesion assays suggest significant defects in *rnp1A* knockdown cells, whereas RNP1A-OE cells showed minimal adhesion defects after shaking.

We next analyzed cytokinesis in *rnp1A* knockdown and RNP1A-OE cells. *rnp1A* knockdown cells exhibited significant cytokinesis defects (Fig. 1G,H; Movies 1, 2). Wild-type cells assume a cylindrical bridge-shaped cleavage furrow, which represents the existence of the combination of ring constriction forces and traction forces (Jahan and Yumura, 2017; Zhang and Robinson, 2005). In *rnp1A* knockdown cells, 58% assumed a bridge-shaped furrow (wild type-like) whereas the remaining 42% of *rnp1A* knockdown cells assumed a V-shaped furrow, resembling cytokinesis A, which primarily depends on the constriction forces at the furrow and less on traction forces (Jahan and Yumura, 2017; Nagasaki et al., 2009). In *rnp1A* knockdown cells, only 6 out of 11 cells reached the cross-over point (bridge length=bridge width); the remaining 5 cells did not reach the cross-over point and assumed a morphology between the V-shape and bridge shape. Knockdown cells also failed to maintain pole-to-pole distance after the cross-over point, suggesting defects in cell–substrate adhesion and/or microtubule function (Fig. 1G,H) (Giodini et al., 2002; Jahan and Yumura, 2017; Poirier et al., 2012; Snyder et al., 1983). Myosin II accumulation at the cleavage furrow was unaffected in *rnp1A* knockdown cells (Fig. S1A).

Notably, the RNP1A-OE cells did not exhibit significant cytokinesis defects, abnormal adhesion, or changes in pole-to-pole distance. However, both *rnp1A* knockdown and RNP1A-OE cells showed slower growth in suspension, suggesting additional factors besides adhesion and cytokinesis defects contribute to the slower growth.

### RNP1A lightly enriches near protrusions, is slightly mechanoresponsive, and interacts with IQGAP1

**Fig. 2.**
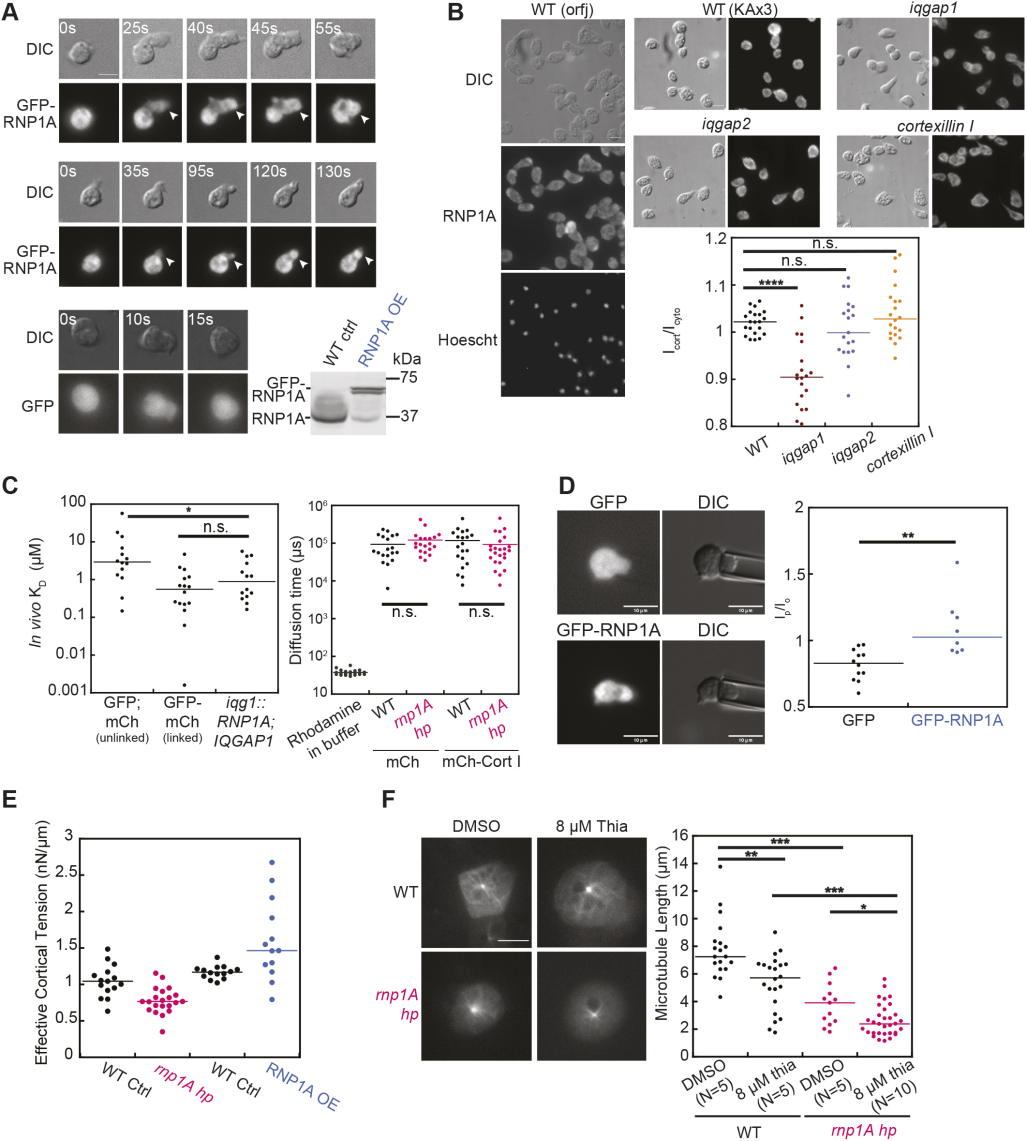
**RNP1A lightly enriches in the cortex, is slightly mechanoresponsive, interacts with IQGAP1 *in vivo* and is important for microtubule polymers*.*** (A) Images of localization of GFP–RNP1A and GFP during random cell migration. Scale bar: 10 µm. Bottom right, western blot analysis of GFP–RNP1A-transformed cells indicating that overexpression of GFP–RNP1A as compared to endogenous RNP1A levels on GFP-transformed cells. Images representative of ten repeats. (B) Left, immunofluorescence images with anti-RNP1A antibodies on fixed wild-type cells (orfJ). Right: immunofluorescence against RNP1A in wild type (KAx3), *iqgap1*-null, *iqgap2*-null and *cortexillin I*-null cells. Cells were fixed with −20°C acetone. To quantify the cortical enrichment of RNP1A, the ratio I_cort_/ I_cyto_ of cortical to cytoplasmic intensity was measured. Data were collected from 20 wild-type (KAx3 background), 19 *iqgap1*-null, 18 *iqgap2*-null, and 19 *cortexillin I*-null cells. Scale bar: 10 µm. (C) Left: *in vivo K*_D_ between unlinked GFP and mCherry (mCh, negative control), linked GFP–mCherry (positive control), and GFP–RNP1A and mCh–IQGAP1, which were measured by FCCS. Data were collected from 13 cells expressing unlinked GFP and mCherry, 15 cells expressing linked GFP–mCherry, and 13 cells expressing GFP–RNP1A and mCh–IQGAP1. The *in vivo K*_D_ between GFP–RNP1A and mCh–IQGAP1 was measured to be 0.89 µM (median). Right, diffusion time of mCherry and mCh–Cortexillin I (Cort I) in wild-type control and *rnp1A* knockdown cells was quantified by FCS. Data were pooled from 18 wild-type control cells expressing mCherry, 18 *rnp1A* knockdown cells expressing mCherry, 19 wild-type control cells expressing mCh–Cortexillin I, 22 *rnp1A* knockdown cells expressing mCh–Cortexillin I, and 20 individual measurements of rhodamine in imaging buffer. (D) Left, images show micropipette aspiration of cells expressing GFP or GFP–RNP1A. Scale bars: 10 µm. Right, the degree of mechanoresponsiveness of GFP–RNP1A was quantified as GFP intensity ratio I_p_/I_o_, the ratio of mean signal intensity inside to outside of the pipette. Data were pooled from 12 wild-type cells (orfJ background) expressing GFP and 8 wild-type cells (orfJ) expressing GFP–RNP1A. (E) Effective cortical tension was quantified using micropipette aspiration. Data were quantified from 15 wild-type control cells and 21 *rnp1A* knockdown cells. (F) Left, wild-type control or *rnp1A* knockdown cells expressing GFP–tubulin were treated with either 8 µM thiabendazole or an equal volume of DMSO vehicle and put under a thin 2% agarose sheet for better visualization. Scale bar: 10 µm. Right, microtubule lengths were quantified manually. Data were pooled from 5 wild-type control cells treated with DMSO, 5 wild-type control cells treated with 8 µM Thiabendazole, 5 *rnp1A* knockdown cells treated with DMSO and 10 *rnp1A* knockdown cells treated with 8 µM Thiabendazole. Each dot represents the measurement from a single microtubule filament. *P≤0.05; **P≤0.01; ***P≤0.001; ****P≤0.0001; n.s., not significant (Kruskal–Wallis followed by Wilcoxon–Mann–Whitney test). WT, wild type; OE, overexpression.

In a previous study, we reported RNP1A enrichment at the protrusive front during chemotaxis (Ngo et al., 2016). Here, we examined GFP–RNP1A distribution during random migration. GFP control did not exhibit any enrichment at the protrusive front (Fig. 2A; Movie 3). GFP–RNP1A became occasionally lightly enriched in the cytoplasm around sites where protrusions formed (Fig. 2A; Movie 4). This light enrichment might reflect the lack of strong polarity cues in randomly migrating cells, as RNP1A enriched more consistently at the protrusive front of polarized chemotaxing cells (Ngo et al., 2016). Quantification of intensity ratios for GFP and GFP–RNP1A in the cytoplasm of protrusions relative to the back portion of the cytoplasm, normalized to an mCherry volumetric control is provided in Fig. S1B. Also, total expression RNP1A when we expressed GFP–RNP1A in wild-type cells is 1.4× endogenous levels, which is similar to the level of overexpression of the unlabeled RNP1A (Fig. 2A versus Fig. 1A).

Next, we investigated endogenous RNP1A protein localization, using immunofluorescence imaging in wild-type and different CK protein null backgrounds to determine whether CK proteins influence RNP1A localization. RNP1A was more cortically enriched in all wild-type KAx3 and null mutants than in the wild-type orfJ (Ax3-derived, replicase orf+; see Materials and Methods for details) background, highlighting a difference in the parental strains (Fig. 2B). Among the different CK mutants, an *iqgap1*-null mutant exhibited less RNP1A cortical enrichment than seen in the wild type and other strains (Fig. 2B).

We then investigated RNP1A–IQGAP1 interactions using fluorescence cross-correlation spectroscopy (FCCS). *In vivo*, RNP1A and IQGAP1 interact with an apparent *in vivo K*_D_ of 0.88 μM in the cytosol (Fig. 2C, left). Please note that FCCS assesses an apparent *in vivo* K_D_ that reflects direct and indirect associations between proteins. This *in vivo K*_D_ is distinct from a thermodynamic *K*_D_, which is generally assessed in a well-defined *in vitro* environment. Furthermore, FCCS data are typically widely spread and log normal due to the viscoelastic milieu of the cytoplasm. Yet, with FCCS, we have had success in detecting biologically relevant direct and indirect biochemical interactions (Kothari et al., 2019b; Nguyen and Robinson, 2022; Plaza-Rodriguez et al., 2022; West-Foyle et al., 2018). Thus, although FCCS cannot determine whether this interaction is direct or mediated by other CK proteins, it supports the role of IQGAP1 in regulating RNP1A localization. As IQGAP2, another key signaling protein in the CKs, also interacts with cortexillin I (Kothari et al., 2019b), we attempted to perform FCCS between RNP1A and IQGAP2. However, attempts to assess RNP1A–IQGAP2 interactions via FCCS were inconclusive as cells did not readily co-express labeled RNP1A and labeled IQGAP2.

As RBPs have been proposed to act as protein–protein interaction hubs (Chen and Mayr, 2022), we tested whether *rnp1A* knockdown alters the overall size of cortexillin I-containing CKs by measuring the *in vivo* diffusion time of mCherry–cortexillin I in wild-type control and *rnp1A* knockdown cells. No significant differences were observed between wild-type and knockdown cells, suggesting that RNP1A reduction does not significantly impact CK complex size (Fig. 2C, right). However, the potential effects of complete *rnp1A* knockout remains untested due to essential nature of RNP1A*.*

We next assessed the mechanoresponsiveness of RNP1A using micropipette aspiration (MPA). MPA on wild-type (orfJ) cells expressing GFP–RNP1A revealed slight mechanoresponsiveness with increased GFP intensity in the cytoplasm within the micropipette relative to outside the pipette (Fig. 2D). Note that GFP alone has a mean intensity ratio below 1, typically ∼0.8, due to the difference in the thickness of the cell within the micropipette compared to outside the micropipette (Kee et al., 2012; Luo et al., 2013; Ren et al., 2009). GFP–RNP1A by contrast had a mean *I*_p_/*I*_o_ ratio of 1.1; for comparison, strong mechanoresponsive proteins such as myosin II have *I*_p_/*I*_o_ ratios typically in the range of ∼1.3–1.5 (Kee et al., 2012; Luo et al., 2013; Ren et al., 2009). Overall, the enrichment of RNP1A aligns with the existence of ‘ambiguous CKs’ containing both IQGAP1 and IQGAP2 (Plaza-Rodriguez et al., 2022). Under agarose compression, GFP–RNP1A appeared to enrich into aggregates within the cytoplasm further illustrating its potential role in mechanoresponsiveness (Fig. S1C).

### RNP1A is important for cortical mechanics and microtubule polymer stabilization

We investigated the role of RNP1A in cellular mechanics. Using MPA to measure effective cortical tension, *rnp1A* knockdown cells exhibited a 20% reduction and RNP1A overexpression cells exhibited a 37% increase in effective cortical tension compared to wild-type controls (Fig. 2E). This reduction coincided with decreased cortical F-actin levels in *rnp1A* knockdown cells (Fig. S1D,E). This cortical tension decrease is also consistent with the cortical tension reductions we have measured previously for single- and double-null mutant combinations of many of the CK components, including myosin II, IQGAP1 and IQGAP2, cortexillin I and II, and discoidin. In these mutants, cortical tension can be reduced by ∼20–80%, depending on the specific mutant scenario (Kee et al., 2012; Nguyen and Robinson, 2022; Reichl et al., 2008). Additionally, cortical tension was unaffected in wild-type cells subjected to 2 and 4 h of starvation as compared to growth conditions (Fig. S1F), which is important to note as we found that *rnp1A* knockdown cells take on a starvation-like gene expression profile (next section).

We originally identified RNP1A (Ngo et al., 2016) in the same genetic selection where we identified 14-3-3 as a genetic suppressor of nocodazole. We then found that 14-3-3 regulates myosin II bipolar filament assembly by binding to its heavy chain, inhibiting its ability to assembly (West-Foyle et al., 2018; Zhou et al., 2010). Therefore, we tested whether *rnp1A* knockdown sensitized microtubules to thiabendazole, a microtubule polymerization inhibitor. We measured microtubule length in wild-type control cells and *rnp1A* knockdown cells under 0 μM and 8 μM thiabendazole, which were put under a thin agarose sheet for better visualization (Fig. 2F). Treatment with 8 μM thiabendazole significantly decreased microtubule lengths in wild-type cells. Under both 0 μM and 8 μM thiabendazole treatment, *rnp1A* knockdown cells had reduced microtubule lengths as compared to that in wild-type cells (Fig. 2F). Our previous study also indicated that microtubule polymer stabilization is important for cortical mechanics where treatment with 10 μM nocodazole reduced cortical tension by 60% (Zhou et al., 2010), suggesting that the destabilization of microtubule polymers in *rnp1A* knockdown cells might also contribute to their reduced cortical tension.

### RNP1A knockdown poises cells to shift away from the vegetative growth to a more developmental-like transcriptional profile

**Fig. 3.**
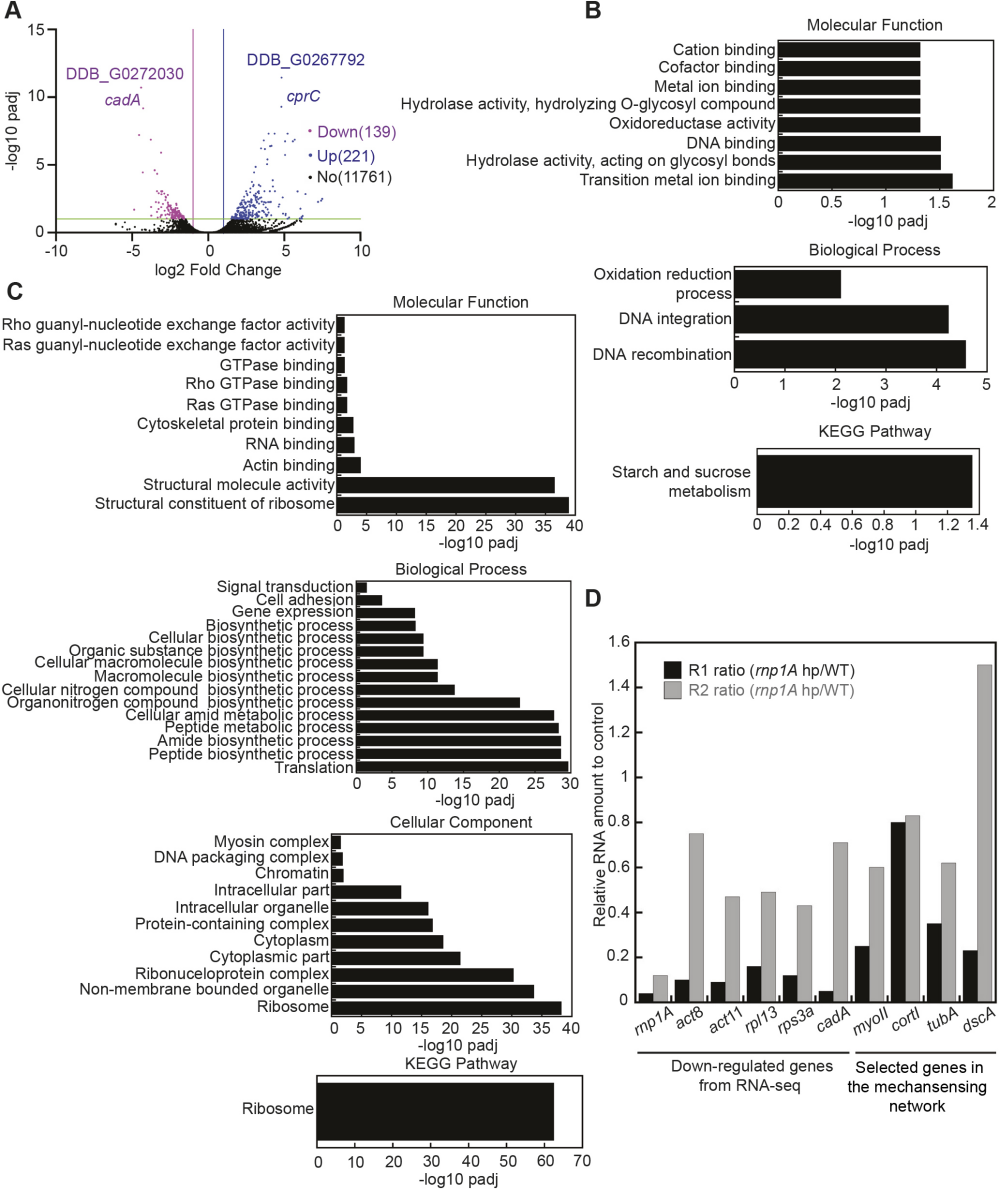
**RNA-seq of *rnp1A* knockdown cells suggests that RNP1A knockdown reduces the expression of genes involved in translation and macropinocytosis.** (A) Volcano plot of differentially expressed genes from RNA-seq of *rnp1A* knockdown cells is shown (replicate 1). Differentially expressed genes are at least twofold up- or down-regulated, and their corresponding padjs are equal or smaller than 0.1. Two of the most significantly up- or down-regulated genes are labeled with DDB_ID or gene names. (B) Gene ontology analysis of upregulated genes from RNA-seq of *rnp1A* knockdown cells (replicate 1). Gene ontology identification threshold is padj≪0.05. (C) Gene ontology analysis of downregulated genes from RNA-seq of *rnp1A* knockdown cells (replicate 1). Gene ontology identification threshold is padj≪0.05. (D) Gene expression level of selected significantly downregulated genes and genes encoding proteins of the mechanoresponsive network compared to control cells from RNA-seq are shown for both replicate 1 and replicate 2.

To explore the impact of RNP1A on cell behaviors, we investigated transcriptomic changes in *rnp1A* knockdown cells using RNA sequencing (RNA-seq). Two independent knockdown cell lines (R1 and R2) were generated alongside wild-type controls, with R1 exhibiting a greater knockdown efficiency (96%) than R2 (88%). Consistent with this, R1 knockdown cells displayed more significantly up- and down-regulated genes than R2, suggesting that the effects of RNP1A are concentration dependent (Fig. 3A; Fig. S2A,B, Tables S1–S4).

Quantitative real-time RT-PCR (qRT-PCR) validation of select downregulated genes confirmed the RNA-seq findings (Fig. S2C). Stronger *rnp1A* knockdown consistently led to greater downregulation of target genes, supporting a positive correlation between *rnp1A* transcript levels and its regulatory impact (Fig. S2C,D). Acute knockdown using the doxycycline-inducible system also reduced transcript levels of select genes after 48 or 100 h, demonstrating that RNP1A mediates both acute and chronic gene expression changes (Fig. S2D). The differential gene expression found in acute knockdown versus chronic knockdown aligns with our two initial independent knockdown cell lines, R1 and R2, where the gene expression profiles vary (Fig. 3A). Conversely, overexpression of RNP1A resulted in reduced transcript levels for *act/act11* and *dscA*, but no significant changes in *rpl13* or *rps3a*, highlighting context-dependent effects of RNP1A on gene regulation and the nuanced impact of transcription data to downstream phenotypes (Fig. S2E).

Upregulated genes were enriched for hydrolase and oxidoreductase activity, including carbohydrate catabolism genes (*nagD*, *celA* and *alfA*) and cytochrome P450 family members, which are implicated in lipid and fatty acid metabolism (Fig. 3B). Starch and sucrose metabolism, a hallmark of spore cells (Kin et al., 2018), was also upregulated. Importantly, 53% of upregulated genes overlapped with those enriched in developmental cell types compared to vegetative cells, further supporting a shift toward a developmental-like state (Table S5). This was confirmed by accelerated chemotactic aggregation of *rnp1A* knockdown cells under starvation conditions (Fig. S3A).

Among the downregulated genes in R1, pathway analysis revealed reduced expression in ribosomal genes, with translation identified as the most significantly suppressed biological process. Genes involved in cell adhesion were also significantly downregulated, aligning with the reduced adhesion phenotype of *rnp1A* knockdown cells (Fig. 3C). As genes encoding translation are highly expressed during the vegetative phase (Kin et al., 2018), their suppression in *rnp1A* knockdown suggests that loss of RNP1A might transition the cells toward a developmental-like phase. In fact, western blot analysis of developing KAx3 cells reveal reduced expression of RNP1A through developmental progression (Fig. S3B).

Interestingly, genes involved in the mechanoresponsive CK system, including *mhcA*, *tubA*, *ctxA* and *dscA*, showed reduced expression in *rnp1A* knockdown cells (R1 and R2; Fig. 3D). Significantly downregulated genes such as *act8*, *act11*, *rp13* and *cadA* exhibited more pronounced reduction in R1 cells, suggesting a correlation between the extent of *rnp1A* knockdown and gene suppression. We then measured expression of these genes in fresh *rnp1A* knockdown cells using both constitutive knockdown and Dox-inducible knockdown and RNP1A-OE cells (Fig. S2C–F). Transcripts were consistently reduced, but protein expression remained the same upon knockdown of *rnp1A*.

### Identification of RNA transcripts to which RNP1A binds

**Fig. 4.**
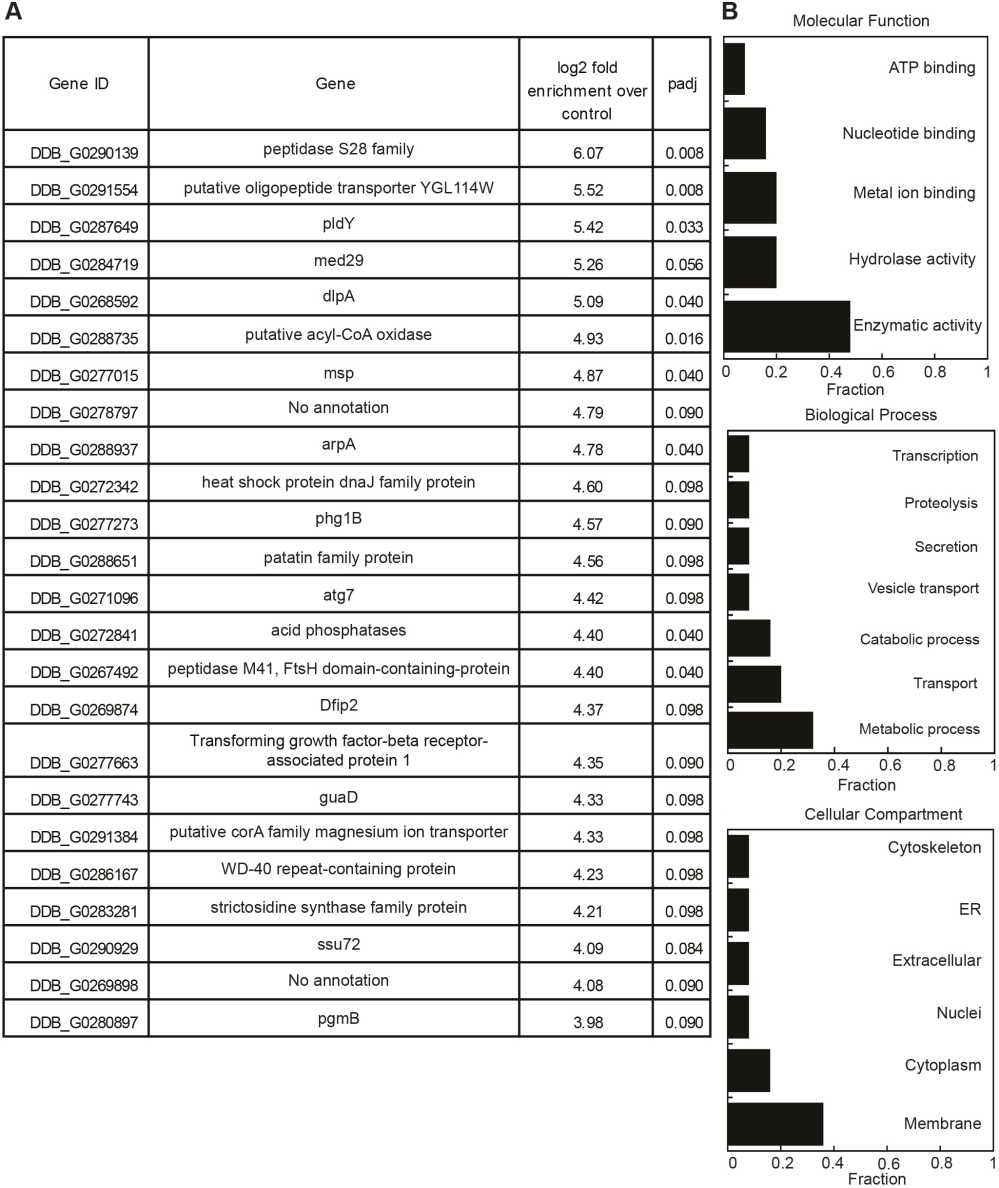
**CLIP-seq revealed mRNA transcripts to which RNP1A binds.** (A) Full list of mRNA transcripts bound to GFP–RNP1A. Another transcript, WD repeat-containing protein 70 (DDB_G0283495; padj=0.102), is not included in the list due to its padj being slightly higher than 0.1. (B) Annotation of mRNA transcripts bound by GFP–RNP1A grouped by molecular functions, biological processes, and cellular components, and presented as fraction of all 24 transcripts identified to be bound to RNP1A.

RNP1A has been found to bind non-coding RNAs (ncRNAs) involved in *D. discoideum* development (Avesson et al., 2011), confirming its role as an RBP that associates with non-messenger RNAs. To further investigate the functional roles of RNP1A roles, we used cross-linking immunoprecipitation (CLIP)-seq to identify the transcripts to which it binds. CLIP-seq analysis identified 24 mRNA transcripts that bind RNP1A with over 90% confidence [adjusted *P*-value (padj)≪0.1] (Fig. 4A). Among the 24 transcripts identified, one transcript (DDB_G0283281, strictosidine synthase family protein) was also significantly upregulated in *rnp1A* knockdown cell lines (R1 and R2) as shown by RNA-seq. Furthermore, several molecular functions associated with these 24 transcripts, such as hydrolase activities and metal ion binding, overlap with function of genes upregulated in *rnp1A* knockdown cells (Figs 3B, 4B).

One transcript encodes DlpA, a protein found to be important for cytokinesis, as *dlpA*-null mutants exhibit cytokinetic defects (Masud Rana et al., 2013). DlpA localizes to the phagocytic cup during phagocytosis (Fujimoto et al., 2019), an endocytic process evolutionarily linked to macropinocytosis and sharing similar biochemical pathways (Vines and King, 2019). Additionally, several transcripts encode proteins with metabolic functions, such as peptidase S28 family proteins, putative acyl-CoA oxidase, pldY, peptidase M41, FtsH domain-containing protein and msp. Although these proteins lack direct evidence connecting them to macropinocytosis, they might help facilitate nutrient digestion during macropinosome maturation (Vines and King, 2019).

### RNP1A contributes to macropinocytosis

Macropinocytosis is the main nutrient uptake pathway for *D. discoideum* cells during fluid-phase growth (Hacker et al., 1997; Williams and Kay, 2018). Reduced macropinocytosis causes starvation, triggering developmental programs and suppressing genes related to macropinocytosis and translation (Williams and Kay, 2018). Therefore, we hypothesized that *rnp1A* knockdown might reduce macropinocytosis given the reduction in translation-encoding genes and its binding to transcripts like *dlpA*.

**Fig. 5.**
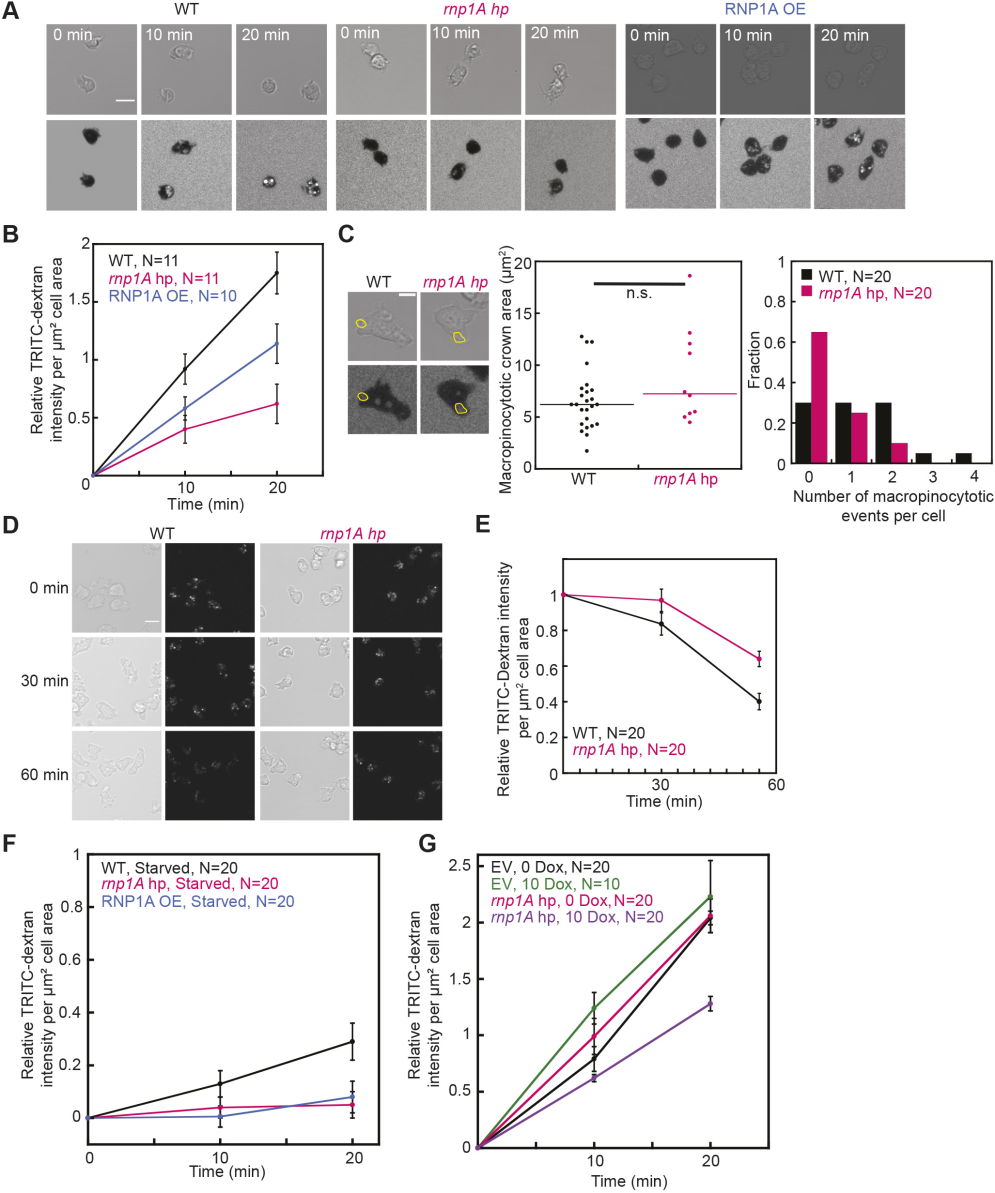
***rnp1A* knockdown cells have macropinocytosis defects.** (A,B) TRITC–Dextran uptake in wild-type, *rnp1A* knockdown and RNP1A-overexpressing cells over the span of 20 min. TRITC–Dextran intensity was quantified by mean TRITC intensity of each cell, background subtracted, normalized to cell area, and then normalized to that at the first time point. Scale bar: 10 µm. Data were pooled from 11 wild-type control, 11 *rnp1A* knockdown and 10 RNP1A*-*overexpressing cells. Error bars show s.e.m. (C) Quantification of macropinocytotic crown area and number of macropinocytotic events per cell over the span of 2 min. Macropinocytotic crowns were manually traced as shown in yellow outlined areas in images at the first timepoint of crown membrane closure. Scale bar: 5 µm. Data were pooled from 20 wild-type control cells and 20 *rnp1A* knockdown cells. n.s., not significant (Kruskal–Wallis followed by Wilcoxon–Mann–Whitney). (D,E) Images showing loss of internalized TRITC–Dextran signal in wild-type control and *rnp1A* knockdown cells over the span of 60 min. Scale bar: 10 µm. TRITC–Dextran intensity was quantified by mean TRITC intensity of each cell, background subtracted, normalized to cell area, and then normalized to the first timepoint. Error bars show s.e.m. Data were pooled from 20 wild-type control cells and 20 *rnp1A* knockdown cells. (F,G) TRITC–Dextran uptake in starved *rnp1A* knockdown, RNP1A-overexpressing cells and doxycycline-induced *rnp1A* knockdown cells. Data were pooled from 10 or 20 cells as indicated in the figure. Error bars show s.e.m. WT, wild type.

We tested macropinocytotic activity using DQ^TM^ Red BSA, which reflects both uptake and macropinosome maturation as its readout is dependent on lysosomal activity. Indeed, *rnp1A* knockdown cells had reduced DQ^TM^ Red BSA signal (Fig. S4A). However, because DQ^TM^ Red BSA cannot distinguish between macropinocytotic uptake and macropinosome maturation, we then used TRITC–Dextran, which cannot be processed in the lysosomes. We examined uptake and found that *rnp1A* knockdown cells had reduced TRITC–Dextran uptake as compared to that in wild-type controls (Fig. 5A,B**)**. Overexpression of RNP1A also reduced TRITC–Dextran uptake, though less severely (Fig. 5B). Macropinosome crown formation was unaffected in *rnp1A* knockdown cells, but these cells showed fewer macropinocytotic events per cell compared to controls (Fig. 5C). Then, we examined loss of TRITC–Dextran and found that *rnp1A* knockdown cells had slower loss of TRITC–Dextran (Fig. 5D,E). Furthermore, the GFP-tagged RNP1A signal was slightly enriched in the cytoplasm around the macropinosome region between macropinosome formation and early macropinosome retraction into the cell body as compared to GFP (Fig. 8A,B; Movies 5, 6).

Starvation slows macropinocytosis, including internalization and endocytic material transfer (Williams and Kay, 2018). This effect could contribute to the reduction in macropinocytosis frequency in *rnp1A* knockdown cells. All cell types, including *rnp1A* knockdown and overexpressing cells, had reduced TRITC–Dextran uptake after 2 h of starvation (Fig. 5F; Fig. S3C). We then asked whether long-term *rnp1A* knockdown led to a long-term starvation and reduction in TRITC–Dextran uptake. Therefore, we tested the effect of acute *rnp1A* knockdown using doxycycline-inducible cells, and these cells also exhibited reduced macropinocytosis, although less severely than persistent knockdown (Fig. 5G versus 5B; Fig. S3D).

**Fig. 6.**
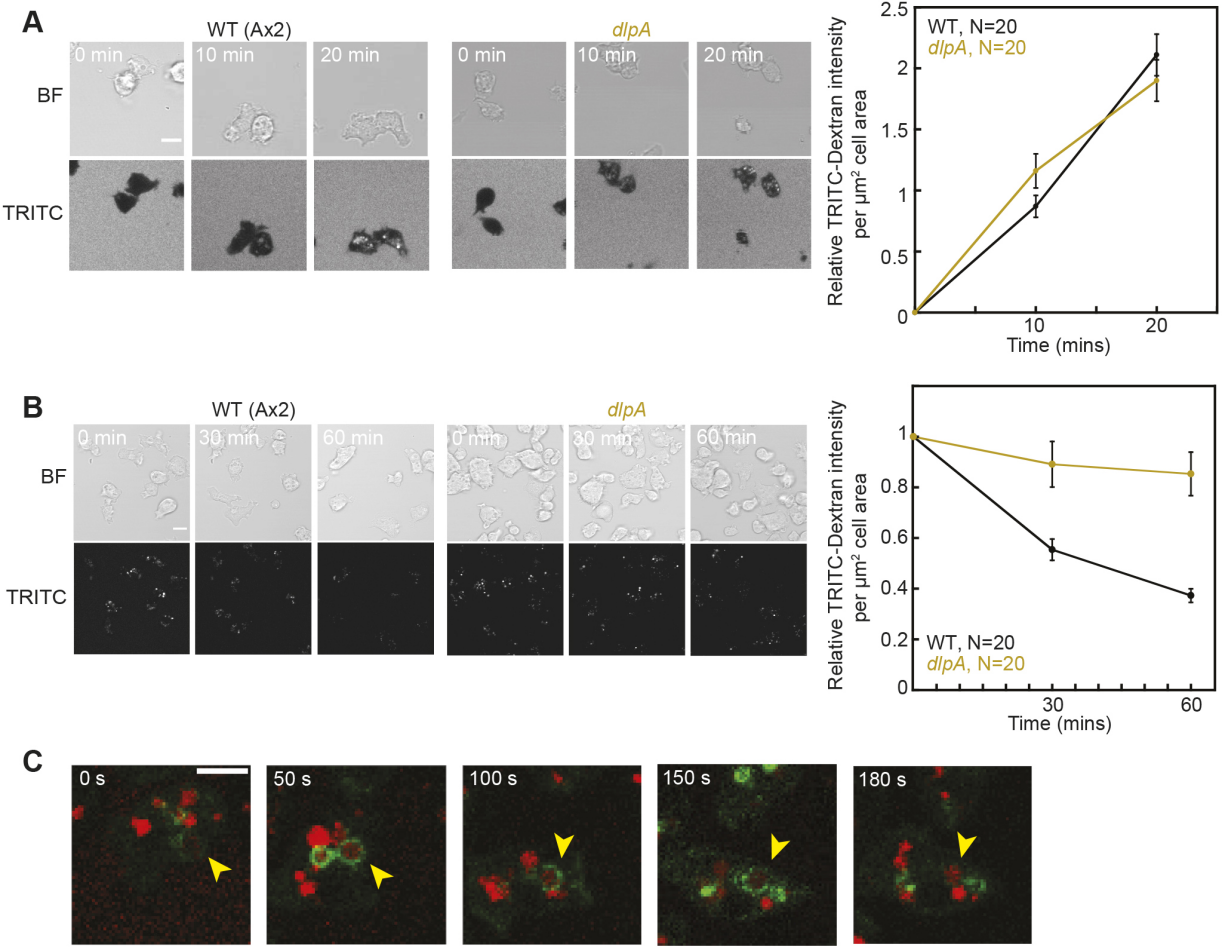
***dlpA*-null cells have delayed loss of internalized TRITC–Dextran, and GFP–DlpA persists around macropinosome for over 150 s.** (A) TRITC–Dextran uptake in wild-type (Ax2) and *dlpA*-null cells over the span of 20 min. TRITC–Dextran intensity was quantified by determining the mean TRITC intensity of each cell, background subtraction and normalization to cell area, and was then normalized to that at the first timepoint. Scale bar: 5 µm. (B) Internalized TRITC–Dextran removal in wild-type (Ax2) and *dlpA* cells over the span of 60 min. TRITC–Dextran intensity was quantified as in A. Scale bar: 10 µm. Data in A and B were pooled from 20 wild-type cells (Ax2) and 20 *dlpA-*null cells. Error bars show s.e.m. (C) GFP–DlpA localization around macropinosome during and after internalization of TRITC–Dextran. Green, GFP–DlpA; red, TRITC–Dextran. Arrowheads point to the same macropinosome over time. Images representative of five repeats. Scale bar: 10 µm. WT, wild type; BF, bright field.

To assess whether macropinocytosis activity is also regulated by RNP1A-bound transcripts, we studied macropinocytosis in *dlpA* mutant cells. GFP–DlpA localized to macropinocytotic crowns and remained on macropinosomes after internalization (Fig. 8C; Movie 7). The *dlpA*-null cells (Miyagishima et al., 2008) showed no decrease in TRITC–Dextran uptake (Fig. 6A), but when using DQ™ Red BSA, *dlpA*-null cells exhibited slower clearance of internalized material and reduced lysosomal degradation activity relative to that in wild type, indicating a role in macropinosome maturation (Fig. 6B; Fig. S4B). This result indicates that DlpA is involved in macropinosome maturation when nutrients in macropinosomes are degraded for cellular use. Newly formed macropinosomes do not mature until after ∼60 s, when V-ATPase is delivered to macropinosomes; DlpA persisted on macropinosomes throughout its maturation (Fig. 6C), coinciding with V-ATPase delivery required for maturation (Cardelli, 2001).

### CK proteins interact genetically with RNP1A to promote macropinocytosis

**Fig. 7.**
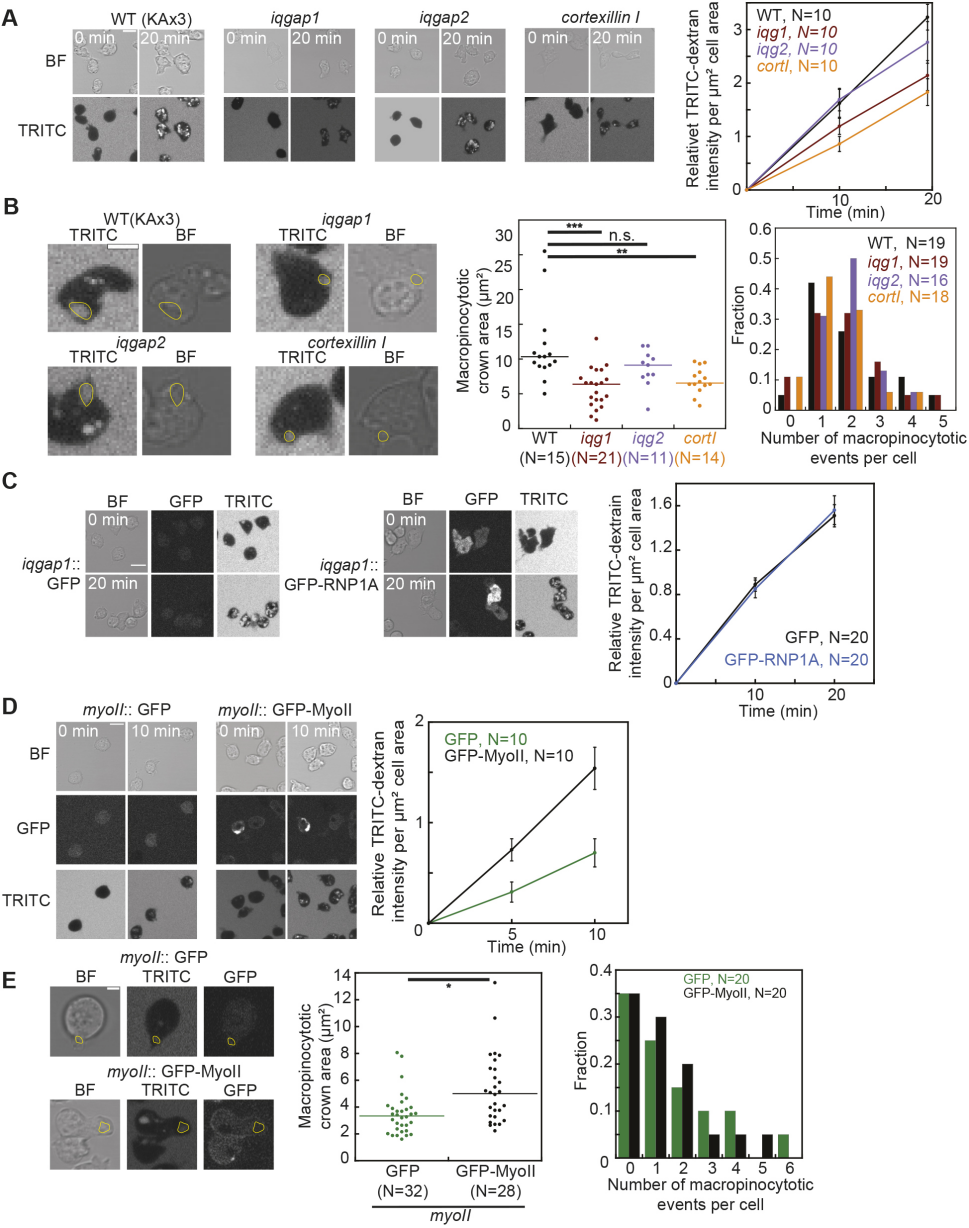
***iqgap1*-null, *cortexillin I*-null, and *myosin II*-null cells show defects in macropinocytosis.** (A) TRITC–Dextran uptake in wild-type (KAx3), *iqgap1* (*iqg1*), *iqgap2* (*iqg2*) and *cortexillin I* (*cortI*) cells over the span of 20 min. TRITC–Dextran intensity was quantified by determining the mean TRITC intensity of each cell, background subtraction and normalization to cell area, and was then normalized to that at the first timepoint. Scale bar: 10 µm. Error bars are s.e.m. errors. Data were pooled from 10 cells/cell line. (B) Quantification of macropinocytotic crown area and number of macropinocytotic events per cell over the span of 2 min for wild type (KAx3), *iqgap1*, *iqgap2* and *cortexillin I* cells. Macropinocytotic crowns were manually traced upon closure as shown in yellow outlined areas in images. Scale bar: 5 µm. Data were pooled from 10 cells/cell line. The specific number of crowns measured and events scored are indicated on the graphs. (C) TRITC–Dextran uptake in *iqgap1*-null cells expressing GFP and GFP–RNP1A. TRITC–Dextran intensity was quantified as in A. Scale bar: 10 µm. Error bars are s.e.m. Data is pooled from 20 cells/cell line. (D) TRITC–Dextran uptake in *myosin II* (*myoII*)-null cells, expressing GFP or GFP–myosin II, over the span of 10 min. TRITC–Dextran intensity was quantified as in A. Scale bar: 10 µm. Error bars are s.e.m. Data were pooled from 10 cells/cell line. (E) Quantification of macropinocytotic crown area and number of macropinocytotic events per cell over the span of 2 min in *myoII*::GFP and *myoII*::GFP-MyoII cells. Macropinocytotic crowns were manually traced as in B. Scale bar: 5 µm. Data were pooled from measurements from 20 cells/cell line. The specific number of crowns measured, and events scored are indicated on the graphs. *P≤0.05; **P≤0.01; ***P≤0.001; n.s., not significant (Kruskal–Wallis followed by Wilcoxon–Mann–Whitney test). BF, bright field.

**Fig. 8.**
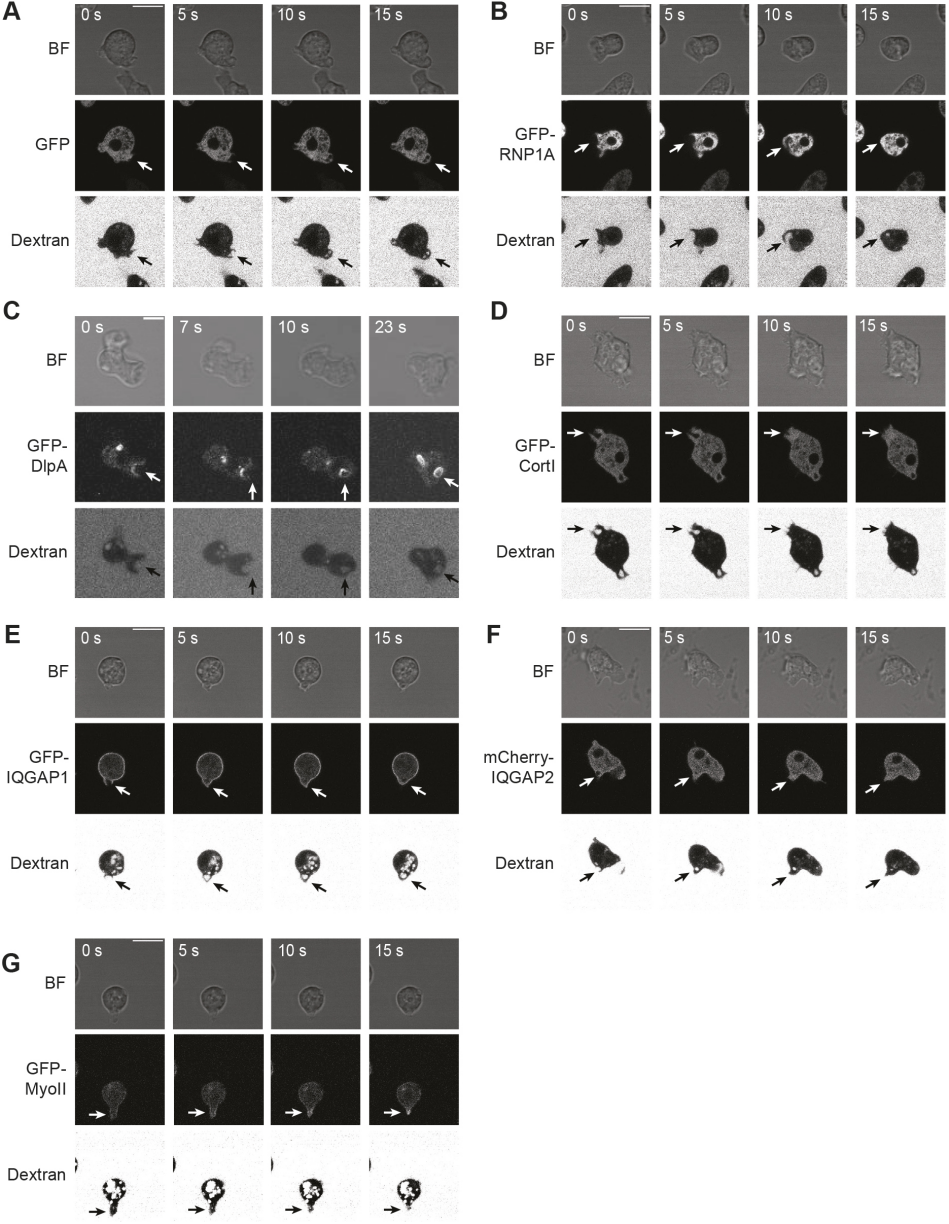
**Localization of RNP1A, DlpA and CK proteins during macropinocytotic crown formation and closure.** Localization of GFP and GFP fusion proteins during macropinocytotic crown formation and closure: (A) GFP; (B) GFP–RNP1A; (C) GFP–DlpA; (D) GFP–cortexillin I; (E) GFP–IQGAP1; (F) mCherry–IQGAP2; and (G) GFP–myosin II. Arrows point to sites of macropinocytotic crown formation. Dextran refers to the fluorescent Dextran (TRITC–Dextran or Cascade Blue–Dextran) for all panels. Scale bars: 10 µm. Images representative of three repeats. BF, bright field.

As RNP1A interacts with the CK proteins cortexillin I and IQGAP1, we next asked whether these two proteins along with myosin II and IQGAP2 contribute to macropinocytosis (Fig. 7). We assessed macropinocytosis in *cortexillin I*-, *iqgap1*-, and *iqgap2*-null cells as they were all generated in the KAx3 background. Compared to wild-type cells, *cortexillin I*-null and *iqgap1*-null cells had decreased TRITC–Dextran uptake and smaller macropinosome area, whereas *iqgap2*-null cells exhibited no defect in TRITC–Dextran uptake and macropinosome size (Fig. 7A,B). As compared to wild-type parental cells (1.9 average events/cell), none of these null mutants showed significant differences in numbers of macropinocytotic events within a 2-min interval (*cortexillin I*, 1.5; *iqgap1*, 1.9, and *iqgap2*, 1.9 average events/cell; Wilcoxon *P*>0.1 for all comparisons; Fig. 7B). The defect of macropinocytosis caused by loss of cortexillin I and IQGAP1 is then due to decreased macropinosome size, which is consistent with their roles in cell shape regulation, but distinct from what caused the macropinocytotic defect in *rnp1A* knockdown cells. We then assessed the localization of these proteins during macropinocytosis. GFP–cortexillin I was enriched at the cell cortex around the site of macropinosome formation (Fig. 8D). GFP–IQGAP1 remained cortically localized and did not show distinct changes in localization, whereas the mCherry–IQGAP2 signal only became slightly enriched at the cell cortex after membrane closure (Fig. 8E,F; Movies 8, 9).

We next explored whether RNP1A and IQGAP1 have any crosstalk during macropinocytosis given that IQGAP1 interacts with RNP1A *in vivo* and contributes to RNP1A cortical localization. We observed no difference in GFP–RNP1A localization during macropinocytosis in *iqgap1*-null cells compared to that in wild-type control cells (Fig. S5A). However, when we overexpressed RNP1A using GFP–RNP1A in *iqgap1*-null cells, RNP1A overexpression did not lead to defects in macropinocytosis (Fig. 7C), as it did in wild-type cells. This observation indicates that RNP1A overexpression works through IQGAP1 to cause macropinocytotic defects, suggesting a crosstalk between RNP1A and IQGAP1 during macropinocytosis.

Then, we assessed the involvement of myosin II in macropinocytosis. We observed that as compared to *myosin II*::GFP–myosin II (*myoII* rescue; wild type) cells, *myosin II*-null cells exhibited less TRITC–Dextran uptake and formed smaller macropinosomes (Fig. 7D). The *myosin II*-null (*myoII*::GFP) cells had a similar number of macropinocytotic events/cell (average, 1.5) to that seen in the *myoII* rescue cells (1.2 events/cell; Wilcoxon *P*=0.77) (Fig. 7E). GFP–myosin II also enriched at the cell cortex behind the retracting macropinosome (Fig. 8G; Fig. S5C, Movie 10). Moreover, both over-assembled (3×Ala) or under-assembled (3×Asp) myosin II (Hostetter et al., 2004) reduced TRITC–Dextran uptake as well, suggesting that wild-type myosin II assembly dynamics contribute to normal macropinocytosis (Fig. S5B). Both mutants displayed reduction in macropinosome area compared to wild-type cells (Fig. S5D). The 3×Asp-expressing mutant, however, exhibited a higher fraction of cells that had only one macropinocytotic event per cell, and none that had more than three macropinocytotic events per cell during a 2-min interval (Fig. S5D). Thus, compared to wild type-rescued cells, the 3×Asp mutant was less efficient in performing macropinocytosis. Finally, GFP–RNP1A overexpression did not cause additional macropinocytotic defects in *myosin II*-null cells (Fig. S5A,B versus Fig. 5C,D), similar to the absence of induced defects in *iqgap1*-null cells. These observations suggest crosstalk between RNP1A and myosin II during macropinocytosis.

As F-actin is important in the initiation of macropinocytosis, we investigated how F-actin localizes in relation to RNP1A, myosin II, cortexillin I and IQGAP1 localization. We expressed an F-actin reporter, limEΔcoil, in rescue cell lines and introduced Cascade Blue–Dextran to observe macropinocytosis. In WT, *myosin II*, *cortexillin I* and *iqgap1* rescued cell lines, the corresponding proteins localized as expected, and actin localized to macropinocytosis crowns (Fig. S5E). Finally, we observed that in *iqgap1*-null, *cortexillin I*-null and *myosin II-null* cells, GFP–DlpA localization during macropinocytosis remained similar to what we observed in wild-type cells (Fig. S5F), indicating DlpA is not dependent on CK proteins for its localization.

## DISCUSSION

Previous work has found that RNA-binding proteins are involved in multiple biological processes (Gerstberger et al., 2014; Hentze et al., 2018). For example, in *D. discoideum*, evidence suggests that RNA-binding proteins, such as Pumillo, help localize specific RNA transcripts to subcellular locations where the encoded protein functions (Hotz and Nelson, 2017). Other RBPs in *D. discoideum* have been shown to function in post-transcriptional regulation (Bai et al., 2021), miRNA processing (Meier et al., 2016) and miRNA maturation (Kruse et al., 2016). However, the function of RBPs within the mechanoresponsive system and the relationship with CK proteins had not been explored. Previously, we discovered that RNP1A interacts with cortexillin I (Kothari et al., 2019b) and is a genetic suppressor of nocodazole when overexpressed (Ngo et al., 2016). Here, we find that RNP1A is important for cell growth, adhesion and cytokinesis. We discovered that RNP1A interacts genetically with CK protein IQGAP1, is slightly mechanoresponsive and contributes to cortical tension. Consistent with our previous study, where we found that RNP1A overexpression protects microtubules from nocodazole (Ngo et al., 2016), here we found that loss of *rnp1A* leads to reduced microtubule mass. Furthermore, knockdown of *rnp1A* shifts cells away from vegetative growth to a more developmental stage-like transcriptional profile, and consistent with this, we found that when *Dictyostelium* cells undergo starvation, RNP1A protein declines. RNP1A binds to transcripts that encode proteins involved in macropinocytosis and works alongside CK proteins to facilitate macropinocytosis. In sum, our studies suggest that RNP1A works with the CK system and interacts with transcripts that encode macropinocytotic proteins to facilitate macropinocytosis and, in so doing, helps maintain *D. discoideum* vegetative growth.

The *rnp1A* knockdown phenotypes of growth and adhesion defects are linked to gene expression changes. Alterations in the gene expression profile suggest a shift away from a vegetative growth state in *rnp1A* knockdown cells, which is consistent with the reduced vegetative growth rate and macropinocytotic defects. Similarly, we found cell adhesion genes are downregulated through our RNA-seq data, and we observed adhesion defects in *rnp1A* knockdown cells as well. During early development, *Dictyostelium* cells modulate substrate adhesion as they prepare for aggregation (Huber et al., 2017; Tarantola et al., 2014). Consistent with this, *rnp1A* knockdown cells show downregulation of key vegetative adhesion genes, such as *sibA*. Although levels of *cadA*, a gene involved in cell–cell adhesion during development, are also reduced, its function is distinct from cell–substrate adhesion and might not contribute directly to the observed phenotype. These results suggest that RNP1A is involved in vegetative adhesion by supporting expression of cell–substrate adhesion genes that are normally repressed during developmental progression. We also observed aberrant cytokinesis in *rnp1A* knockdown cells, but not in RNP1A-OE cells. Morphologically, the inability to form the bridge-shaped cleavage furrow is similar to phenotype observed in cytokinesis A (Nagasaki et al., 2009), where cells depend on constriction forces rather than traction forces to complete cytokinesis, reflecting a reduction in cell–substrate adhesion. Additionally, myosin II still localizes to the cleavage furrow cortex during cytokinesis in *rnp1A* knockdown cells, suggesting RNP1A affects cytokinesis differently. We also observed that *rnp1A* knockdown and RNP1A-OE cells exhibit some similar trends phenotypically (growth, adhesion, gene expression and macropinocytosis), which suggests that optimal ‘wild-type’ expression of RNP1A is required for normal cell behavior.

We previously described *in vivo* interactions between RNP1A and cortexillin I (Kothari et al., 2019b). Here, we discovered that RNP1A interacts genetically with IQGAP1, a negative regulator of the mechanoresponsive CK network (Kee et al., 2012). RNP1A localization does not exhibit the distinctive cortical localization of cortexillin I (Cha and Jeon, 2011; Faix et al., 1996) or IQGAP1 in the wild-type orfJ [Ax3(Rep orf+)] parental strain. RNP1A does have slightly enriched cortical distribution in the wild-type KAx3 parental strain, suggesting an inherent difference between these parental strains. Interestingly, IQGAP1 binds to active Rac1A (Faix et al., 1998), and Rac1A localizes to the macropinocytotic crowns (Williams et al., 2019), which trends similarly with RNP1A localization during macropinocytosis. GFP-tagged RNP1A was slightly mechanoresponsive (i.e. it had a slight accumulation in the cytoplasm locally in response to imposed mechanical stress), which is consistent with the interaction of RNP1A with cortexillin I, a mechanoresponsive protein.

RBPs play a role in gene expression via multiple mechanisms (Ray et al., 2013). Polyadenylate-binding proteins (PABPs) have high sequence similarities with RNP1A (Ngo et al., 2016). PABPC helps regulate RNA polyadenylation, translation, localization and decay (Wigington et al., 2014). PABPC also localizes to the leading edge of migrating cells (Woods et al., 2002), similar to the localization of RNP1A. Another class of proteins that have high sequence similarities with RNP1A are the cold-induced RNA-binding proteins, including Cirbp and Rbm3. These two proteins help regulate RNA translation and stability by controlling alternative polyadenylation (Liu et al., 2013). Rbm3 also localizes to invasive pseudopodia of mesenchymal breast cancer cells (Mardakheh et al., 2015) and regulates cell spreading and migration (Pilotte et al., 2018). Although characterized in different organisms, the similar localization patterns of PABPC and Rbm3 to RNP1A suggest that more functional similarities between this group of proteins may exist.

RNP1A contains two canonical RNA recognition motif (RRM) domains. Having dual RRMs suggests potential for increased RNA-binding affinity and/or sequence specificity, potentially underlying its selective interaction with a subset of the cellular transcripts identified via CLIP-seq. However, our current data do not distinguish whether the phenotypic consequences of RNP1A knockdown – particularly the defects in cell morphology, mechanical integrity and cortical organization – are primarily driven by loss of RNA-binding function or by disruption of protein–protein interactions, as RNP1A does both. As described above, the highly essential nature of RNP1A prevented any further study seeking out what part of RNP1A is necessary. However, the fact that only one of the 24 CLIP-identified RNA targets shows significant expression change upon knockdown further raises the possibility that the cellular functions of RNP1A extend beyond direct post-transcriptional regulation. This disconnect highlights the possibility that RNP1A regulates its RNA targets through mechanisms other than mRNA stability, such as localization, translational control or sequestration. To assess whether RNP1A exhibits sequence specificity in its RNA binding, we analyzed the CLIP-seq peak regions for enriched sequence motifs using the MEME Suite Motif Discovery and Motif Enrichment Analysis tools (https://meme-suite.org/meme/). However, these analyses did not reveal a consistent or statistically significant binding motif among the identified targets. This lack of a clear consensus sequence suggests that RNP1A might not recognize a specific RNA motif with high affinity, or that its binding is driven by other contextual factors, such as RNA secondary structure, local transcript environment or cooperative interactions with other RBPs and potentially even CK components.

What are the possible mechanisms by which RNP1A interacts with transcripts and proteins and how might these activities feed into RNA stability and/or protein translation? First, RNP1A is unlikely to regulate RNA production or turnover during vegetative growth given that only one transcript to which RNP1A binds exhibited gene expression level changes in the *rnp1A* knockdown scenario. Instead, RNP1A more likely regulates localization, translation and/or modification of these transcripts during vegetative growth. Loss of RNP1A could affect the translational output from these transcripts, leading to macropinocytotic defects, which we have observed for the *rnp1A* knockdown cells. Another, but not mutually exclusive possibility is that RNP1A could have transcription factor activity, as it has been indicated that some RBPs interact with chromatin to affect gene expression (Du and Xiao, 2020). Although we cannot rule out this possibility, we suspect this is unlikely as RNP1A did not show localization within the nucleus during vegetative growth. Regarding the possible role of RNP1A regulating translation, given that localized translation takes place on the timescale of hundreds of seconds (Wang et al., 2018), it is less likely that RNP1A directs localized translation concurrently during macropinosome formation, which only takes about a minute from initiation to membrane closure and retraction. Instead, we hypothesize that RNP1A might serve as a mediator of the translation and function of these proteins when the cell is in a highly active, vegetative state.

Macropinocytosis is a biological process where robust cell and cortical shape changes take place. Cell mechanics, including cortical tension (formally, cortical tension is an energy cost of adding a unit of surface area), might help resist and then facilitate the progression of macropinocytosis. During the initiation stage of macropinocytosis, cortical tension is likely to resist macropinocytosis, and therefore, active force production through actin polymer assembly and/or a relaxation of cortical tension might promote macropinocytosis. It could also be that the *rnp1A* knockdown cells experienced starvation, which could have contributed to the reduced cortical tension in the mutant. However, we found that the cortical tension of cells in growth versus starvation medium are similar. Furthermore, in mammalian myoblasts, an acute decrease in plasma membrane tension results in initiation of macropinocytosis via phosphatidic acid production and PI(4,5)P_2_-enriched membrane ruffling (Loh et al., 2019). In the *Hydra vulgaris* outer epithelial layer, mechanical stretch inhibits macropinocytosis via activating stretch-activated channels, including Piezo, leading to Ca^2+^ influx (Skokan et al., 2024). After initiation of macropinocytosis, the macropinocytotic crown membrane continues to elongate and eventually closes. Once elongated, cortical tension along with active inward force production pulls back the cortex, re-rounding the cell.

Myosin proteins play several roles in macropinocytosis. For example, some myosin I isoforms are recruited to the macropinocytotic crowns or cups and form a broad ring around the cortex (Brzeska et al., 2016). Additionally, myosin I isoforms play a role in fluid uptake as endocytic uptake is impacted in myosin I mutants (Jung et al., 1996; Novak et al., 1995). Myosin II has also been implicated in Neuro-2a cells where myosin IIB is essential for macropinocytosis (Jiang et al., 2010). We found that in *D. discoideum*, myosin II and its assembly dynamics are essential for normal macropinocytosis. Myosin II localizes to the cell cortex around the macropinocytotic crown where it helps drive macropinosome retraction after crown closure. The reduced macropinocytosis observed in *myoII*-null cells aligns with previous findings that blebbistatin, a myosin II inhibitor, inhibits fluid uptake in *Dictyostelium* (Shu et al., 2005). This supports a role for myosin II during cortex retraction after the macropinosome has closed.

The involvement of CK proteins in macropinocytosis further indicates that cortical mechanics feed into this cell shape-change process. The transient localization of IQGAP2 right after crown closure likely signifies the transition from macropinocytotic crown formation to retraction, helping recruit myosin II to assist with crown retraction. Interestingly, although the *iqgap2* knockout strain did not exhibit significant defects in macropinocytotic crown size or fluid uptake, the *iqgC* (IQGAP3) mutants have modestly increased fluid uptake and larger macropinosomes (Marinovic et al., 2019). This discrepancy likely reflects differences in IQGAP paralog functions as IQGAP3 has RasGAP activity whereas IQGAP1 and IQGAP2 do not. Finally, the CK mutants did not have significant differences in the frequency of forming macropinocytotic cups from each other or as compared to wild-type controls. Thus, CK proteins are involved in the later steps of macropinocytosis.

Our studies also raise an interesting question: do mechanical stimuli affect RNA regulation in cells, including, but not limited to, RNA localization, translation, processing, decay and/or modification, and vice versa? Although this study does not directly address this connection, the association between RNP1A with the CK machinery, the mRNAs it binds and the association between all these factors with cell shape changes strongly support some level of interconnection between these processes. Some budding evidence from the literature also supports such a connection, suggesting this interconnection will likely turn out to be a more fundamental concept than is currently appreciated. For example, mechanical stimuli can recruit polyA mRNA and ribosomes via focal adhesion and associated proteins (Chicurel et al., 1998). More recently, heterogeneous nuclear ribonucleoprotein C (hnRNPC) has been observed to localize to the cardiomyocyte sarcomeres, and extracellular matrix remodeling in pathological conditions leads to hnRNPC association with the translational machinery. Furthermore, hnRNPC regulates the alternative splicing of transcripts encoding for mechanotransduction proteins (Martino et al., 2022). In contrast, glucocorticoid counteracts mechanotransduction in human skin fibroblasts cells by upregulating a lncRNA that promotes decay of mRNAs encoding mechanosensory proteins (Zhu et al., 2021). Recent work has identified the transcription factor GtaC as a regulator of macropinocytosis, which acts through transcriptional control of nutrient uptake genes in *Dictyostelium* (Hao et al., 2024). In contrast, our data suggest that RNP1A functions post transcriptionally, regulating a distinct set of macropinocytosis-related transcripts. Together, GtaC and RNP1A might represent complementary layers of macropinocytosis regulation in response to environmental conditions. Recent models propose that local membrane tension drives macropinosome closure, whereas global cortical tension influences cup dynamics (Lutton et al., 2023; Saito and Sawai, 2021). Our findings support this, as reduced cortical tension in *rnp1A* knockdown and CK mutant cells correlates with impaired macropinocytosis, linking mechanical regulation to the efficiency of this process. This collection of studies in addition to our present study of RNP1A, which revealed an intersection between mRNAs, CK machinery, and dynamic cell shape change processes like macropinocytosis, strongly supports the interconnectedness of these cellular subsystems.

Overall, although it should be noted that RNP1A is a very essential gene, which limited some of the approaches we could take, by examining the function of RNP1A from a variety of angles, we have been able to deduce a significant role for RNP1A in a host of cellular processes. Specifically, RNP1A contributes to cell mechanics, cytokinesis, cell substrate adhesion, genetic and biochemical interactions with other CK components, gene expression, RNA binding and macropinocytosis (Table S6). The multi-angle study draws off a range of approaches that include genetic disruptions, protein–protein interactions, bulk RNAseq, CLIP-seq, mechanical studies and cell assays. Collectively, we found that RNP1A is a key player in the integration of the CK network with a broad range of cellular processes, and this system integration and cellular programing is becoming a powerful hallmark of mechanobiology (e.g. Balaban et al., 2023).

## MATERIALS AND METHODS

### Expression plasmids and cell strains

Generation of the plasmids for GFP–mCherry (linked), and GFP- and/or mCherry-tagged fusions of RNP1A, tubulin, myosin II, myosin II 3×Asp, myosin II 3×Ala, cortexillin I, IQGAP1 and IQGAP2 have been described previously (Effler et al., 2006; Kee et al., 2012; Luo et al., 2013; Ngo et al., 2016; Wheatley and Wang, 1996). GFP–DlpA was obtained from NBRP Nenkin.

Cell strain nomenclature lists the strain or the mutant gene first, then a double colon (::) is inserted before the protein/construct being expressed is listed. Finally, the vector in which the protein/construct is being expressed is listed. In most cases, we match the plasmid backbone between controls and the lines with a protein being expressed. However, in the case of *myoII*::GFP-myoII:pBIG, the GFP protein alone is being expressed using the pDM181 vector in the same mutant cell line that has had *myoII* (*mhcA*) knocked out. Also, gene names are italicized, while the names of proteins being expressed are non-italicized.

We used cell lines from three different backgrounds, and the mutants are matched with the appropriate parental and/or rescued strain when possible. The *cortI*, *iqgap1* and *iqgap2* lines were generated in the KAx3 wild type background and were originally acquired from the laboratory of Rick Firtel (Division of Biological Sciences, University of California, San Diego, USA). The *myoII* mutant was originally generated in the JH10 background. Because the JH10 parental strain is no longer available, we always compare the *myoII* null, carrying either an empty plasmid or a GFP expression plasmid to the rescued cell line. We also use the Ax3 strain, which contains the open reading frame for the Ddp2-based plasmid replicase; this cell line is referred to as Ax3(Rep orf+) or orfJS (orfJ for short) as these cells were originally acquired from laboratory of Jim Spudich (Department of Biochemistry, Stanford University, USA). Note that for rescued lines for the knockout cell lines, these cells have been well studied and validated across many earlier papers from our group (e.g. Effler et al., 2006; Kee et al., 2012; Luo et al., 2013).

### Cell culture and maintenance

*D. discoideum* cells were maintained in Hans' enriched HL-5 medium (1.4× HL-5 medium with 8% FM (FM Minimal Medium, ForMedium, ref. no. FMM0102), penicillin (FisherBiotech, ref. no. BP914A-100) and streptomycin (Sigma-Aldrich, ref. no. S6501-25G) at 22°C in Petri dishes (Thermo Fisher Scientific; FB0875712) (Robinson and Spudich, 2000). A full list of strains used in this study are provided in Table S7. Cells transformed with plasmids were grown in HL-5 medium supplemented with corresponding selection drug [10 μg/ml or 15 μg/ml G418 (Corning®, ref. no. 61-234-RG); 40 μg/ml hygromycin (Invitrogen, ref. no. 10687010)].

### Generation of *rnp*1A knockdown cell line

We first attempted a genetic deletion using CRISPR. For this, the CRISPR guide RNAs used were as follows: guide 1: 5′-CCAACAAAAATTCTGTGGGC-3′; guide 2: 5′-ATTCTGTGGGCTGGAGTAGT-3′. Because we were unable to generate knockout cells using standard CRISPR, we then attempted the SorMC method using *K. aerogenes* adapted from (Paschke et al., 2018)*. D. discoideum* were grown with *K. aerogenes* as an alternative to grow *D. discoideum* and screen for *rnp1A* knockouts. *K. aerogenes* were grown overnight in LB medium at 37°C under 270 rpm shaking conditions. Bacteria were then harvested by centrifuging at 6000 ***g*** for 20 min at 4°C. Supernatant was poured off, and the remaining pellet was washed with SorMC (15 mM KH_2_PO_4_, 2 mM Na_2_HPO_4_, 50 µM MgCl_2_, 50 µM CaCl_2_) by pipetting up and down with serological pipette. Cell suspension was then centrifuged once more at 6000 ***g*** for 20 min at 4°C. Supernatant was aspirated, and the pellet was resuspended in SorMC to an optical density at 600 nm (OD_600_) stock solution of 100. *D. discoideum* cells were grown in Petri dishes under bacterial suspensions diluted to an OD_600_ of 2 in SorMC.

Wild-type *D. discoideum* (KAx3 background) cells were grown to a log phase before transformation. On the day of transformation, plates containing *D. discoideum* were gently washed once with 10 ml of SorMC to remove residual culture medium. *D. discoideum* cells were then resuspended with 5 ml of SorMC and transferred into a 15 ml conical. Cells were then counted with a hemocytometer, and concentrations were determined. *D. discoideum* cells were then centrifuged at 1000 ***g*** for 5 min with their supernatant subsequently poured off. The remaining *D. discoideum* pellet was resuspended to a cell density of 10^7^ cells/350 µl with pre-chilled electroporation buffer (50 mM sucrose, 10 mM KH_2_PO_4_/K_2_HPO_4_ pH 6.5). Resuspended cells were then transferred into a 4 mm-wide pre-chilled electroporation cuvette along with 1 µg of either pTM1285 or *rnp1A*:pTM1285 plasmid DNA. Cells were electroporated at 1.3 kV voltage and 3.0 µF capacitance. Electroporated cells were then transferred into a pre-chilled 10 cm plate containing no drug plus *K. aerogenes* in SorMC (OD_600_=2) growth medium to rest and grow overnight. The following day after transformation, the no drug medium was replaced with 10 ml of 10 µg/ml G418 in SorMC *K. aerogenes* growth medium for selection. Medium was replaced 2 days later with fresh selection medium to remove dead cells. The *K. aerogenes* culture were grown overnight in 100 ml of LB at 37C. The following day, the overnight *K. aerogenes* solution was used to resuspend the *D. discoideum* under selection to make 1:100, 1:1000, and 1:10,000 dilutions. 1 ml of the dilutions were plated onto an SM-5 plate for monoclonal plaque selection. SM-5 plates were grown in a 22°C incubator, and plaques developed over 3–7 days. Plaques were picked and transferred to an individual well of a 24-well plate containing 1 ml of 10 µg/ml G418K*. aerogenes* SorMC growth medium. Cells were monitored to ensure proper growth and were then moved into a 10 cm plate to expand. Knockout candidates were made into cell lysates and processed through a western blot analysis to screen for knockouts.

### Generation of *rnp*1A knockdown cell line

For the *rnp*1A knockdown strains, we used the Ax3(Rep orf+) (orfJ) wild-type strain as the parental background. Knockdowns were generated using RNA hairpin targeting the 3′ of cDNA sequence. Using the pLD1A15SN plasmid (Robinson and Spudich, 2000), the following sequences were cloned into the plasmid to create the RNA hairpin: sense strand: 5′-CGGTTTCGTCGAATTCGATGATGTTGCCAATCAACAAAAAGGTCTCACCCTTAACAAACTCTCTGTTGAAAGTAGAGAACTCTCTGTTAAAATCGCTTTAGTTCCAGAACCAAGAGATGCCACCGCAACTACTCCAGATGTTACCACTACCGCT-3′; anti-sense strand: 5′-AGCGGTAGTGGTAACATCTGGAGTAGTTGCGGTGGCATCTCTTGGTTCTGGAACTAAAGCGATTTTAACAGAGAGTTCTCTACTTTCAACAGAGAGTTTGTTAAGGGTGAGACCTTTTTGTTGATTGGCAACATCATCGAATTCGACGAAACCGAAACCTTTGCTTCTGTTGGTGTGTTTGTTGACAATGACATGAGCACTCTTTGGTGAGCAATCTTTGAAGGTTTCTAATAATTTAACATCATCAAAAGAGTATGGAATGTTTCTGACAAAGAGGGTAGTGGTACTTTGTTGTCTGTCAGCAGTGTTGGCAGCTGG-3′.

Cells were transformed with 1 µg empty control plasmid or plasmid containing the *rnp1A* hairpin on day 0 and incubated in medium without selection drug (G418) for 2 days. On day 2, the medium was replaced with medium containing 15 µg/ml G418. On day 4, the medium was changed to reduce drug selection to 7.5 µg/ml G418. On day 6, medium was changed again to reduce G418 to 5 µg/ml. From then on, medium was changed with medium containing 5 µg/ml G418 every 2 days until cell colonies were visible on the substrate of cell culture dishes. Then, medium was replaced every other day to increase G418 concentration by 1 µg/ml/change until 10 µg/ml was reached. 10 µg/ml G418 concentration was maintained from then on. *rnp1A* mRNA knockdown was verified and quantified by real time PCR, and protein knockdown was verified and quantified by western analysis.

The doxycycline-inducible *rnp1A* knockdown cell line was generated using doxycycline-inducible expression vector (pDM310), containing the same *rnp1A* hairpin sequence described above. 10 μg/ml doxycycline was used to induce expression and the induction lasted for either 48 h for qRT-PCR analysis or 100 h for western analysis.

### Growth assays

For suspension growth, cells were grown in suspension culture in flasks with a starting cell density of 10^4^ cells/ml. For the substrate growth assay, cells were grown in eight-well imaging chambers. Cell counts were taken every 24 h. Cell growth rate were determined from the exponential growth phase. Relative growth rates were calculated as growth rate of individual samples divided by the average growth rates of control cell lines.

### Adhesion assays

*D. discoideum* cells were plated on non-tissue culture six-well plates for an hour at a 10^6^ cells/ml density. Then, after gentle manual rotation to mix free cells, 200 μl samples were taken from the culture medium from each well, and cell counts were measured. The six-well plates were shaken on a shaking platform at 50, 75 or 100 rpm for 30 min at room temperature. After shaking, 200 μl samples were again collected from the culture medium from each well, and cell counts were measured. The fraction of cells non-adherent was calculated as the density of cells in culture medium divided by the total density of cells on the plates. The change in fraction of cells that were non-adherent was calculated by subtracting the control fraction of non-adherent cells from mutant fraction of non-adherent cells.

### Immunofluorescence and imaging

Cells were plated in eight-well slide chamber (Lab-Tek) at a density of 1.5×10^6^ cells/ml for 30 min. Cell culture medium was removed, and cells were fixed in −20°C acetone for 5 min or 2% PFA for 10 min. Cells were washed once with PBST (1× PBS plus 0.05% Triton X-100) and then incubated with 3% BSA in PBST for 1 h at room temperature. Primary antibody immunostaining was performed using a 1:10,000 dilution in PBST at 4°C overnight. Then, cells were washed three time with PBST. Secondary antibody immunostaining was performed using 1:3000 dilution in 3% BSA in PBST for 1 h at room temperature. Rhodamine–phalloidin staining was performed using 1:200 dilution in PBST with 3% BSA for 1 h at room temperature. Cells were again washed with PBST twice before Hoechst 33342 (Thermo Fisher Scientific, ref. #62249) staining (10 μg/ml in 3% BSA in PBST) for 10 min at room temperature. Slides were mounted with Invitrogen ProLong Diamond Antifade Mountant (P36961). Images were acquired with an Olympus IX71 epifluorescence microscope or with a Zeiss AxioObserver. Images were processed and quantified using Fiji (Schindelin et al., 2012). A list of antibodies used in this study is provided in Table S8.

### Cortical-cytoplasmic ratio intensity measurement analysis

ImageJ was used to measure the signal intensity of the cortex and cytoplasm in each cell line. The line and freehand region of interest (ROI) tools were used to capture the cortex and cytoplasmic regions of the cells. The intensity density of the cortex and cytoplasm were used for the ratio calculation. The signal intensities from both the cortex and cytoplasm were background subtracted before doing analysis. Excel was used to calculate the ratio of signal by dividing the cortical signal by the cytoplasmic signal.

### Quantification and analysis of GFP–RNP1A localization during random migration

Wild-type cells expressing GFP:pLD1A15SN; mCherry:pDRH and GFP-RNP1A:pLD1A15SN; mCherry:pDRH were imaged on a Zeiss LSM 800 confocal light laser scanning microscope, using a 63× oil 1.4NA objective. A 5-min time lapse videos with 5 s intervals were acquired, capturing the fluorescence of GFP and mCherry as well as an electronically switchable illumination and detection (ESID) acquisition. Timelapse videos were analyzed in Fiji using the ESID channel to identify leading edges of the cell. In the frame where a leading edge of the cell was identified, a ROI was defined and measured in both the GFP and mCherry channel. In the same cell, another ROI was traced along the rear of the cell and measured in the GFP and mCherry channel. The background mean of the image was then subtracted from the mean measurements acquired. The GFP signal of the cell at the leading edge was divided by the GFP signal at the rear of the cell. The ratiometric value calculated was then normalized by the value of dividing the mCherry signal at the leading edge of the cell by the mCherry signal at the rear. The final value was plotted onto a dot plot within KaleidaGraph. Additional graphs were generated within KaleidaGraph plotting the normalized ratiometric values of GFP control and GFP–RNP1A at the leading edge against the cell body (not including the leading edge) total integrated intensity.

### Nuclei per cell measurement

Immunofluorescence staining was performed as described above. Cells in exponential growth phase were grown in Petri dishes prior to fixation using the 2% paraformaldehyde protocol. The number of nuclei per cell were manually counted. The cell boundary was confirmed in the DIC channel to identify when a cell is multinucleated, and not simply two cells adhering to each other, which is very rare situation with vegetatively growing *Dictyostelium* cells. Statistical analysis was performed using comparison of proportions.

### Cytokinesis imaging

Live-cell images were taken at 2 s intervals for 5–10 min. Time-lapse imaging was initiated at the beginning of cleavage furrow formation and terminated after cell division completed or failed. Cleavage furrow or intercellular bridge length, diameter, and the distance between two poles of the dividing cells were manually tracked and measured using ImageJ.

### Generation of RNP1A antibody and western analysis

RNP1A antibody was developed by AbClonal. Full-length RNP1A peptide was used as antigen. Four immunizations were performed on two rabbits every 2 weeks. After the 4th immunization, bleeds were collected from the rabbits and polyclonal antibody was purified from the bleed.

For western analysis, doxycycline-inducible constructs were incubated in 10 μg/ml doxycycline for 100 h. Cell lysates were prepared by boiling cells in SDS sample buffer, electrophoretically separated on SDS-polyacrylamide gels, and transferred to nitrocellulose membranes. Proteins were detected by each individual antibody. A list of antibodies used in this study is provided in Table S8. Images were acquired on a LiCor Odyssey CLx system. Quantification of protein expression level was performed by quantifying intensity of each band on the blot, then background was subtracted and values normalized against total protein amount measured from the Coomassie staining of a replica gel.

For western blot analysis of RNP1A expression during 24 h of development, 10^6^ cells were seeded and incubated in MES starvation buffer (50 mM MES, pH 6.8, 2 mM MgCl_2_, and 0.2 mM CaCl_2_ in deionized water) on a non-tissue culture treated six-well plates at 22°C. Cells were harvested from each well that corresponded to how long they are developed. Cells were lysed in SDS sample buffer and ran electrophoretically as describe above and probed using our anti-RNP1A antibody.

Original source data for western blot and Coomassie-stained gels are provided in Fig. S6.

### Micropipette aspiration, effective cortical tension, and mechanoresponsiveness quantification

MPA was performed with equipment set up as previously described (Kee and Robinson, 2013). Cells were seeded in an imaging chamber at ∼10% confluency at least 30 min before imaging. The micropipette (∼5 μm diameter) was stabilized at the bottom of the cell chamber. To attach cells to the pipette tip, a small aspiration pressure was applied. Then, aspiration pressure was increased gradually to the equilibrium pressure ΔP, where the length of the cell inside the pipette (L_p_) is equal to the radius of the pipette (R_p_). The cell was then released from the pipette tip. After resting for a couple of minutes, a second measurement was performed on the same cell. Effective cortical tension is quantified using the Young–Laplace equation:

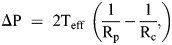
where ΔP=aspiration pressure that produces cell deformation; T_eff_=effective cortical tension; R_p_=radius of the pipette; and R_c_=radius of the cell outside the pipette.

Effective cortical tension for each cell is represented by the average from the two measurements.

To quantify the mechanoresponsiveness of a protein (Effler et al., 2006; Ren et al., 2009), cells were slowly aspirated to 0.80 nN/μm^2^ and held for at least 100 s. Mechanoresponsiveness was then calculated as the ratio of the background-corrected mean signal intensity of the cortex inside the pipette (I_p_) to that of the opposite cortex outside the pipette (I_o_).

### Fluorescence correlation spectroscopy and cross-correlation spectroscopy

Fluorescence correlation spectroscopy (FCS) and fluorescence cross-correlation spectroscopy (FCCS) were performed as previously described (Kothari et al., 2019b). Specifically, cells expressing the corresponding fluorophore-labeled proteins were plated on imaging chambers at least 30 min before imaging. Co-expressed soluble GFP and mCherry served as the negative control and expressed GFP attached to mCherry by a 5-amino-acid flexible linker served as a positive control. System calibration was performed using 100 nM Rhodamine. Experiments were performed on a Zeiss AxioObserver with 780-Quasar confocal module and FCS capability using a C-Apochromat 40× water objective. The ‘apparent *in vivo*’ *K_D_* was calculated using the following equation:

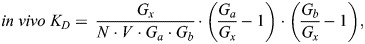
where G_x_=cross-correlation of two fluorophores; G_a_=auto-correlation for mCherry; G_b_=auto-correlation for GFP; N=Avogadro's number; and *V*=confocal volume.

Image data was processed with ZEN software (black edition) using the FCCS analysis module.

### Agarose overlay and microtubule length quantification

Agarose overlay was performed as described previously (Kee et al., 2012). Specifically, thin sheets of 2% agarose gel in water were prepared as follows. Two 22 mm×22 mm cover glasses (Fisherbrand 12542B) were placed at each end of a 22 mm×60 mm microscope cover glass (Fisherbrand 12545J). 1 ml of dissolved agarose solution was placed onto the center of the 22 mm×60 mm microscope cover glass, and a second 22 mm×60 mm microscope cover glass was immediately placed on top to spread the agar evenly between two ends of the cover glass. This method produces thin agarose sheets that are around 0.15 mm thick. Agarose sheets were cut into smaller pieces to fit into imaging chambers (Lab-Tek 155409). Prior to imaging, cells were plated onto imaging chambers and allowed to sit for 30 min. Cell culture medium was removed, and pieces of 2% agarose sheets were placed directly on top of cells in the imaging chamber.

To disassemble microtubules, cells were incubated with 8 μM thiabendazole-treated growth medium at 22°C for 30 min then transferred to an eight-well imaging chamber in which pieces of the 0.15 mm thick 2% agarose were placed directly on top of cells. A negative control was run in parallel with the same volume of DMSO (vehicle). 8 μM of thiabendazole was determined from previous a previous titration curve performed in wild-type orfJ *D. discoideum* cells to optimize microtubule depolymerization with lower concentrations. The GFP-labeled microtubules were traced manually, and lengths were measured using ImageJ.

### RNA-seq

RNA-seq was performed in collaboration with Novogene. Specifically, two replicates of total RNA were harvested from each of the two fresh independent rounds of RNA hairpin knockdown of *rnp1A* (both wild-type control and constitutive *rnp1A* hairpin cell lines; the two independent rounds of RNA hairpin knockdowns are presented as R1 and R2). A total amount of 1 μg RNA per sample was used as input material for the RNA sample preparations. Sequencing libraries were generated using NEBNext® UltraTM RNA Library Prep Kit for Illumina® (NEB, USA) following the manufacturer's recommendations, and index codes were added to attribute sequences to each sample. Briefly, mRNA was purified from total RNA using poly-T oligo-attached magnetic beads. Fragmentation was carried out using divalent cations under elevated temperature in NEBNext First Strand Synthesis Reaction Buffer (5X). First strand cDNA was synthesized using random hexamer primer and M-MuLV Reverse Transcriptase (RNase H-). Second strand cDNA synthesis was subsequently performed using DNA Polymerase I and RNase H. Remaining overhangs were converted into blunt ends via exonuclease/polymerase activities. After adenylation of the 3′ ends of DNA fragments, NEBNext Adaptor with a hairpin loop structure were ligated to prepare for hybridization. To select cDNA fragments of preferentially ∼150–200 bp in length, the library fragments were purified with AMPure XP system (Beckman Coulter, Beverly, USA). Then, 3 μl USER Enzyme (NEB, USA) was used with size-selected, adaptor-ligated cDNA at 37°C for 15 min followed by 5 min at 95°C before PCR. Then, PCR was performed with Phusion High-Fidelity DNA polymerase, Universal PCR primers and Index (X) Primer. Finally, PCR products were purified (AMPure XP system), and library quality was assessed on the Agilent Bioanalyzer 2100 system. The clustering of the index-coded samples was performed on a cBot Cluster Generation System using PE Cluster Kit cBot-HS (Illumina), according to the manufacturer's instructions. After cluster generation, the library preparations were sequenced on NovaSeq 6000 (PE150) and paired-end reads were generated. Raw data (raw reads) in FASTQ format were firstly processed through fastp. Paired-end clean reads were mapped to the reference genome using HISAT2 software. Featurecounts was used to count the read numbers mapped to each gene. And then the RPKM of each gene was calculated based on the length of the gene, and the read count that was mapped to this gene. Differential expression analysis was performed using DESeq2 R package. Gene Ontology (GO) enrichment analysis of differentially expressed genes was implemented by the clusterProfiler R package. We used clusterProfiler R package to test the statistical enrichment of differential expression genes in KEGG pathways.

### RNA extraction and qRT-PCR

RNA extraction was undertaken with TRIzol^TM^ reagent (Thermo Fisher Scientific, 15596026), following the manufacturer's instruction.

qRT-PCR was performed using Verso 1-step RT-qPCR kit (AB4104A). Doxycycline-inducible constructs were incubated in 10 μg/ml doxycycline for 48 h. RNA was extracted from cell lines, and the primers used for qRT-PCR are listed in Table S9. All experiments were conducted on a BIO-RAD CFX Opus 96 Real-Time PCR system. The program used was the following: reverse transcription, 50°C for 15 min, 95°C for 15 min; PCR, 95°C for 15 s, 60°C for 30 s, 72°C for 30 s; melt curve generation, 95°C for 30 s, 60°C for 30 s, and then increased by 0.5°C per 10 s. Quantification of gene expression from qRT-PCR was performed by calculating the differences in C_t_ value between target genes and control gene (*abcF4*; *abcF4* did not exhibit gene expression changes from RNA-seq) for each cell line, and then normalized against the wild-type control cell line.

### CLIP-seq

CLIP-seq protocol was adapted from a previous publication (Muller et al., 2013). Specifically, cells were first washed one time with phosphate buffer and then plated in Petri dishes for UV crosslinking at 250 mJ/cm^2^. Cells were resuspended in modified RIPA buffer (50 mM Tris-HCl pH 7.5, 150 mM NaCl, 0.5% NP40, 0.5% sodium deoxycholate, 1 mM EDTA), and then sonicated at 30% amplitude for 4 min (30 s on, 30 s off). Samples were then centrifuged for three time at 20,000 ***g*** for 15 min at 4°C, and the supernatant was removed. Supernatant was pre-incubated with 250 µl Sephadex G-50 beads in TE buffer. 40 µl was collected as input one. And then supernatant was incubated with 50 µl GFP-trap beads (Chromotek, gta) overnight. On the next day, beads were washed once with RIPA stringency A buffer (50 mM Tris-HCl pH 7.5, 1 M NaCl, 1% NP40, 1% sodium deoxycholate, 1 mM EDTA, 0.1% SDS, 2 M Urea), twice with RIPA stringency B buffer (50 mM Tris-HCl pH7.5, 1 M NaCl, 1% NP40, 1% sodium deoxycholate, 1 mM EDTA, 0.1% SDS, 1 M Urea). 40 µl was collected as input two. Beads were then washed once with equilibration buffer (50 mM Tris-HCl pH 7.5, 300 mM NaCl, 0.5% NP40, 0.5% sodium deoxycholate, 1 mM EDTA, 0.1% SDS). Finally, beads were added to elution buffer (50 mM Tris-HCl pH 7.5, 300 mM NaCl, 0.5% NP-40, 0.5% sodium deoxycholate, 5 mM EDTA, 0.1% SDS, 1 mg/ml proteinase K) and incubated at 42°C for 1 h followed by 56°C for 4 h. RNA was extracted with TRIzol^TM^ reagent (Thermo Fisher Scientific, 15596026). Three replicates (separate pools of cells) per cell line (control, wild-type cells expressing GFP; sample: wild-type cells expressing GFP–RNP1A) were used in downstream sequencing and analysis. RNA samples were converted into double-stranded cDNA using the Ovation RNA-Seq System v2.0 kit (Tecan, Männedorf, Switzerland), which utilizes a proprietary strand displacement technology for linear amplification of mRNA without rRNA/tRNA depletion as per the manufacturer's recommendations. This approach does not retain strand-specific information. Quality and quantity of the resulting cDNA was monitored using the Bioanalyzer High Sensitivity kit (Agilent) which yielded a characteristic smear of cDNA molecules ranging in size from 500 to 2000 nucleotides in length. After shearing 500 ng of cDNA to an average size of 250 nucleotides with the Covaris S4 (Covaris Inc., Woburn, MA, USA) library construction was completed with the Truseq Nano kit (Illumina; San Diego, CA, USA), according to the manufacturer's instructions. mRNA libraries were sequenced on an Illumina Novaseq 6000 instrument using 150 bp paired-end dual indexed reads and 1% of PhiX control. Reads were aligned to the *D. discoideum* reference genome dicty2.7.51. rsem-1.3.0 was used for alignment as well as generating gene expression levels. The ‘rsem-calculate-expression’ module was used with the following options: --star, --calc-ci, --star-output-genome-bam, --forward-prob 0.5. Differential expression analysis and statistical testing were performed using DESeq2 software. Transcripts identified to bind to RNP1A meet the threshold of padj ≪0.1.

### Macropinocytosis measurements

Cells were seeded onto eight-well imaging chambers (Nunc Lab-Tek) at a density of 5×10^5^ cells/ml for 30 min before imaging. Imaging was performed on a Zeiss LSM 780 FCS confocal microscope. Prior to imaging, TRITC–Dextran (Millipore-Sigma; 65–85 kDa) was added to cells at a final concentration of 1 mg/ml. Images were acquired every 30 s for a total duration of 10 or 20 min. We randomly chose 10–20 cells for analysis. Mean TRITC signal intensity at certain time points during the movie for an individual cell was measured, corrected for background, and then normalized to that of the first frame. In the end, the mean±s.e.m. of normalized TRITC intensity over time were calculated for each individual cell line for comparison.

To measure macropinocytotic crown cross-sectional areas (closed macropinosomes), images were taken every second after adding 1 mg/ml TRITC–Dextran for a duration of 2 min. Five to ten cells were randomly chosen from each movie for measurement. We identified the first frame where the macropinocytotic crown membrane closed, and the crown at the identified frame was manually traced to measure the area. The number of macropinocytotic events per cell over the span of 2 min was also recorded for comparison between cell lines. For visualization of RNP1A, DlpA and CK proteins localization during macropinocytosis, GFP- or mCherry-tagged proteins were expressed in different genetic backgrounds, and movies of macropinocytosis were collected as described above. Macropinocytotic events were defined as the complete timeline of macropinocytotic cup formation and closure. Macropinocytotic events in which cup formation or closure was incomplete were not scored.

To measure the rate of loss of internalized TRITC–Dextran in cells, cells were seeded, and TRITC–Dextran was added as described above. After ∼10 min, cells were washed with HL5 medium three times, and fresh HL5 medium was added to the chamber to remove the remaining TRITC–Dextran in the medium. Images were acquired every 30 s for a total duration of 60 min. 20 cells were randomly chosen for analysis described as above.

To quantify the signal of GFP–RNP1A and GFP–myoII in retracting macropinosomes, cells were incubated in 1 mg/ml TRITC-Dextran and were imaged on a Zeiss LSM 780 FCS confocal microscope with a 40× 1.4NA objective. Time lapses of at least 5 min were acquired with 1 s intervals. Macropinosomes were identified using the TRITC–Dextran channel and progression was followed until the macropinosome was fully retracted into the main body of the cell. Quantification of the signal was performed within Fiji by defining ROIs on the outer border of the macropinosome and an additional ROI within a non-macropinocytotic rear region for normalization. The final five frames were quantified once the macropinosome fully retracted into the cell body. All values used for calculations were subtracted by the background mean. The mean signal at the macropinosome was then divided by the mean signal of the non-macropinocytotic rear region. Each frame value was then plotted against time with 0 s being defined as the time at which the macropinosome is fully retracted.

### DQ™ Red BSA degradation assay

*D. discoideum* cells were grown in 1.5× HL-5 medium in 10 cm Petri dishes (Fisherbrand, Catalog #FB0875712). *D. discoideum* cells were resuspended and counted with a hemocytometer. Approximately 100,000 cells in 200 µl low-fluorescent medium were seeded into 96-well plate wells (Falcon^®^, reference 353072) and incubated at 22°C to settle and adhere to the bottom of the plate. DQ™ Red BSA (Thermo Fisher Scientific, catalog number: D12051) was reconstituted with 1 ml of 1× PBS (137 mM NaCl, 2.7 mM KCl, 10 mM Na_2_HPO_4_, 1.8 mM NaH_2_PO_4_) for a 1 mg/ml stock. Growth medium from 96-well plate wells were aspirated off and replaced with 200 µl of low-fluorescent medium and allowed to incubate for 1 h. A FLUOstar Omega plate reader was used to measure background noise using 584 nm excitation and 620 (10 nm bandwidth) emission filters. Low fluorescent medium was aspirated off cells and replaced with 200 µl of a 100 µg/ml DQ™ Red BSA in low-fluorescent medium solution. Cells had DQ™ Red BSA fluorescent signals measured using the plate reader every 5 min for 1 h. Fluorescence signal attained were background subtracted by fluorescence intensity measurement pre-DQ™ Red BSA addition. Data points were plotted across time using KaleidaGraph.

For live-cell DQ™ Red BSA image acquisition, *D. discoideum* cells were prepared as described above. Approximately 250,000 cells in 450 µl were seeded into 4-well chamber wells (Thermo Fisher Scientific, catalog number: 155383) and incubated at 22°C for 1 h to settle and adhere to the glass bottom of the well. 50 µl of a 1 mg/ml DQ™ Red BSA in 1× PBS solution was added to the wells for a final concentration of 100 µg/ml solution in a 500 µl volume. Images were acquired on a Zeiss LSM 800 Confocal Light Laser Scanning Microscope, using a 63× oil 1.4NA objective. Images were acquired every minute for an hour. Images were adjusted for visibility and modified with a scale bar using Fiji.

### Cell aggregation assay

*D. discoideum* cells were grown in 1.5× HL-5 medium in 10 cm Petri dishes (Fisherbrand™, Catalog #FB0875712). *D. discoideum* cells were resuspended and counted with a hemocytometer. Cells were spun down at 1000 ***g*** for 5 min. Cells were resuspended and washed with MES starvation buffer (50 mM MES, pH 6.8, 2 mM MgCl_2_, and 0.2 mM CaCl_2_ in deionized water). Two washes were performed to remove as much growth medium as possible. The washed cells were resuspended to a concentration of 10^6^ cells/ml in MES starvation buffer and 100 µl (100,000 cells) were seeded into each well of a non-tissue culture-treated 96-well plate (Falcon®, Reference 351172). The seeded 96-well plate was immediately imaged every 5 min for a total duration of 11 h on a Molecular Devices ImageXpress High-Content Imager using a 4× air objective in brightfield. Images acquired were analyzed with Fiji. Standard deviation was measured from each frame and plotted across time. Graph was generated using KaleidaGraph.

### Statistical analysis

All statistical analysis was performed with a Kruskal–Wallis test followed by a Wilcoxon–Mann–Whitney test, unless otherwise specified. Annotation used in figures are: *P≤0.05; **P≤0.01; ***P≤0.001; ****P≤0.0001; n.s., not significant.

## Supplementary Material

10.1242/joces.264128_sup1 Supplementary information
