# Supplementary material for: The RNA-binding protein RNP1A is essential and interacts with contractility kit proteins to facilitate cell mechanics
Source: J Cell Sci. 2026 Jan 19;139(1):jcs264128. doi: 10.1242/jcs.264128 (PMC12863306; doi:10.1242/jcs.264128)
Supplement: Supplementary information [file joces-139-264128-s1.pdf]

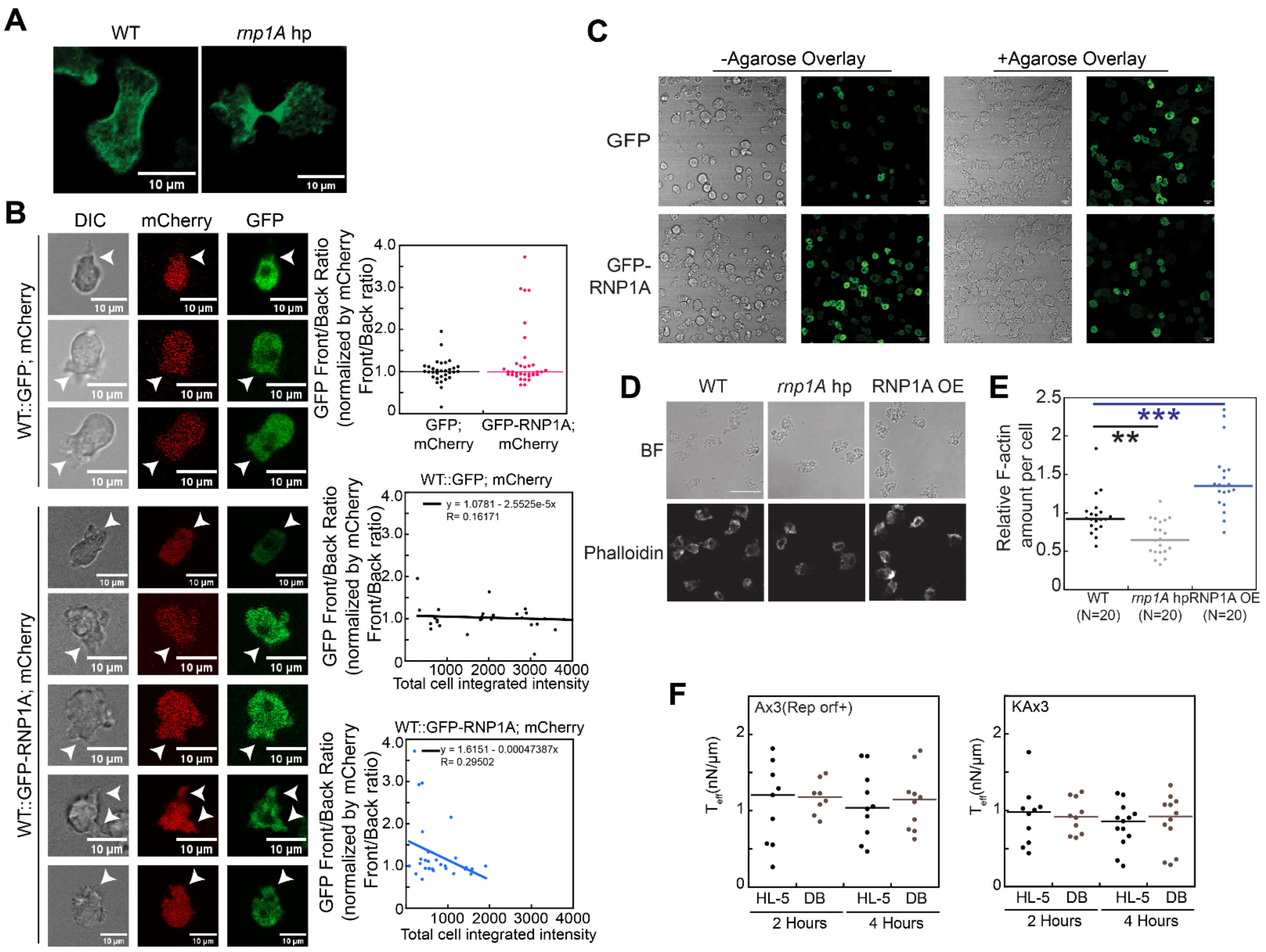

Fig. S1. Characterization of RNP1A's role and impact within the cell. (**A**) Fixed cell imaging of myosin II in WT (orfj) control cells and *rnp1A* hp cells. Cells were fixed with 4% paraformaldehyde. Fixed cell images acquired using a Zeiss LSM800 confocal microscope with a 63x oil 1.4NA objective. Scale bar, 10 µm. (**B**) Cells expressing GFP or GFP-RNP1A had their intensity at the leading edge and back measured in Fiji and normalized by an mCherry ratiometric value calculated by dividing mCherry signal at the leading edge and back (Top graph). Images used to quantify had their background subtracted and leading edges were identified through cell movement in movies. Three sets of WT (orfj)::GFP;mCherry cells and five sets of WT (orfj)::GFP-RNP1A;mCherry images are represented with arrows indicating the leading edge. Additional graphs illustrate the GFP-mCherry ratiometric values plotted against the cell body's (not including leading edge) total integrated intensity (Middle and Bottom graph). A linear regression was applied to the middle and bottom graphs along with the resulting equation. Data were pooled from 30 WT (orfj)::GFP;mCherry and 32 WT (orfj)::GFP-RNP1A;mCherry cells. (**C**) Live cell images show cells expressing GFP or GFP-RNP1A were imaged with and without an agarose compression. Agarose compression images were acquired immediately after agarose overlay introduction. Live cells images acquired by a Zeiss LSM800 confocal microscope with a 63x oil 1.4NA objective. Scale bar, 10 µm. (**D**) Fluorescence images of F-actin in wild type control, *rnp1A*-knockdown, and RNP1A-overexpressing cells labeled with Rhodamine-phalloidin. Cells were fixed with 2%

PFA. Scale bar, 10 µm. (**E**) Quantification of Rhodamine intensity per cell in wild type control, *rnp1A* knockdown, and RNP1A-overexpressing cells. Mean Rhodamine intensity of each cell is measured and normalized against the average of that in wild type control cells. Data were pooled from 20 cells per cell line. Statistical analysis was performed with Kruskal–Wallis followed by Wilcoxon-Mann–Whitney test. **, $P \leq 0.01$; ***, $P \leq 0.001$. (**F**) Micropipette aspiration was conducted on wild type Ax3 (Rep orf+) (orfj) and KAx3 parental cells incubated in MES starvation buffer (DB) with 2- and 4-hr time durations. Cortical tension (T_{eff}, nN/µm) was calculated from MPA data between cells grown in HL-5 growth media versus DB. Cells were incubated in growth or DB buffer for either two or four hours before micropipette aspiration was performed.

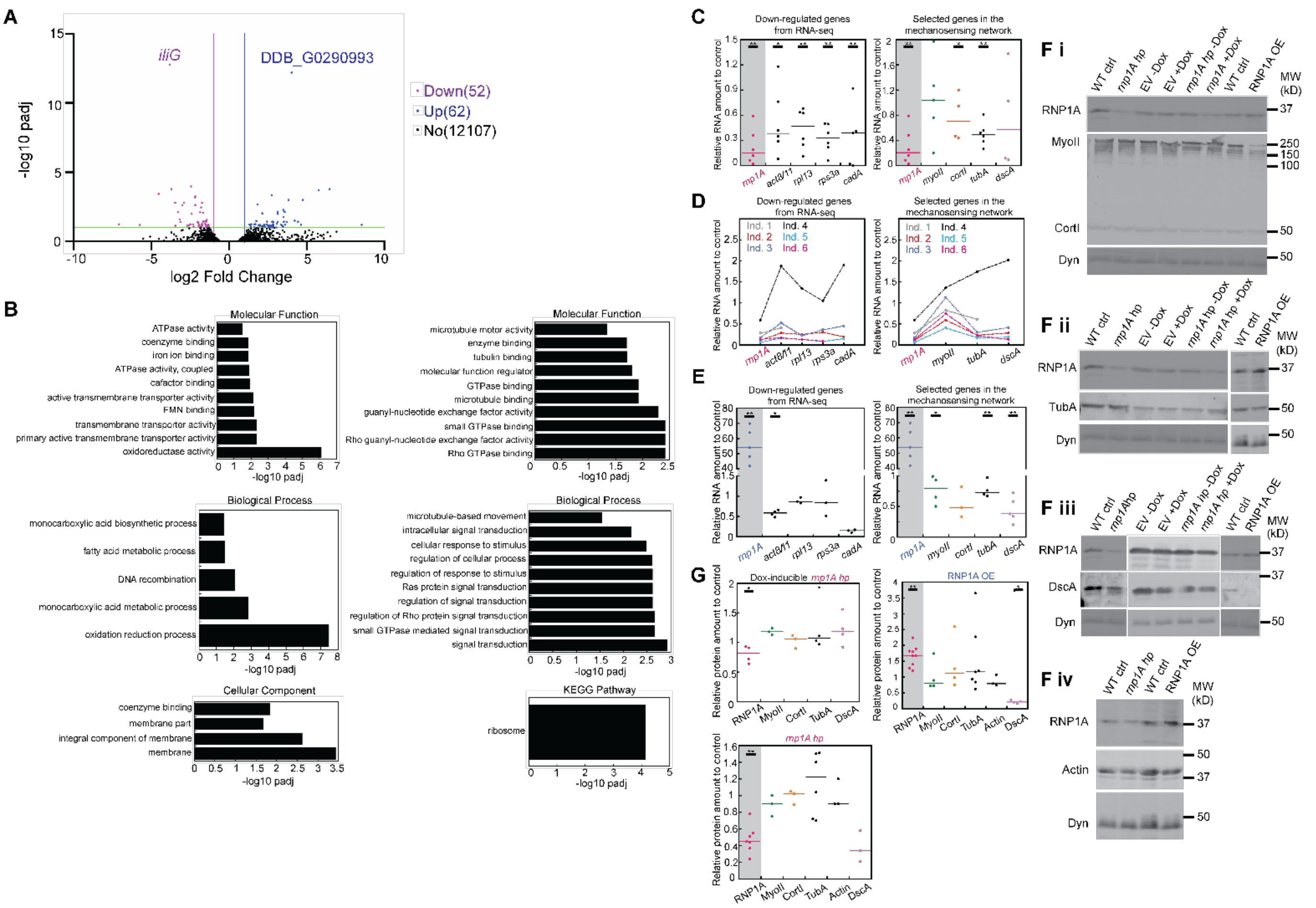

Fig. S2. RNA-seq results from *rnp1A* knockdown cells and RT-qPCR validation. (**A**) Volcano plot of differentially expressed genes from RNA-seq on *rnp1A* knockdown (replicate 2) is shown. Differentially expressed genes are at least two-fold up-regulated or down-regulated, and their corresponding padjs are equal or smaller than 0.1. Most significantly up- or down-regulated genes are labeled with DDB_ID or gene name. (**B**) Gene ontology analysis of up-regulated genes from RNA-seq of *rnp1A* knockdown cells (replicate 2; left column). Gene ontology analysis of down-regulated genes from RNA-seq of *rnp1A* knockdown cells (replicate 2; right column). (**C**) qRT-PCR validation on gene expression levels of selected genes identified from RNA-seq data of *rnp1A* knockdown cells. Each dot represents the gene's relative RNA amount normalized to respective wild-type control determined from a single qRT-PCR run. Data were pooled from measurements of at least three biological replicates. Each value is normalized to their respective control with black bars and asterisks representing statistical significance of p <0.05. (**D**) qRT-PCR validation of expression of selected genes in doxycycline-inducible *rnp1A* knockdown cells. Each dot represents a measurement from a single qRT-PCR run. The dots connected by dotted lines are qRT-PCR measurements from the same doxycycline induction (Ind.). (**E**) qRT-PCR-determined gene expression levels of selected genes in RNP1A-overexpressing cells. Each dot represents the relative RNA amount determined from a single qRT-PCR run. Data were pooled from measurements from at least two biological replicates. Each value is normalized to their respective control with black bars and asterisks representing statistical significance of p<0.05. (**F**) Western blot analysis of **i**) Myosin II and Cortexillin I, **ii**) Alpha Tubulin, and **iii**) Discoidin 1A protein expression in *rnp1A* knockdown, *rnp1A* doxycycline-inducible knockdown, and RNP1A-overexpressing cells. **iv**) Western analysis of actin expression was also tested in *rnp1A* knockdown and RNP1A-overexpressing cells. Dynacortin served

as a loading control. Relative protein expression normalized to control is provided for Dox-inducible *rnp1A* knockdown and RNP1A-overexpression cells. Western blot data were analyzed against total protein amount extracted from Coomassie staining of replica gels. EV is the empty vector control. Original blots are provided in the Blot Transparency file. **(G)** Quantification of protein amounts of selected proteins in the CK network. Each dot represents the relative protein amount determined from a single blot. Each value is normalized to their respective control with black bars and asterisks representing statistical significance of $p<0.05$. For quantification in *rnp1A* knockdown and overexpressing cells, data were pooled from measurements from at least two biological replicates. For measurement in doxycycline-inducible knockdown cells, data were pooled from at least three individual doxycycline inductions. Data information: all statistical analysis was done with Kruskal–Wallis followed by Wilcoxon-Mann–Whitney test. *, $P\leq0.05$; **, $P\leq0.01$; unlabeled, not significant.

Journal of Cell Science • Supplementary information

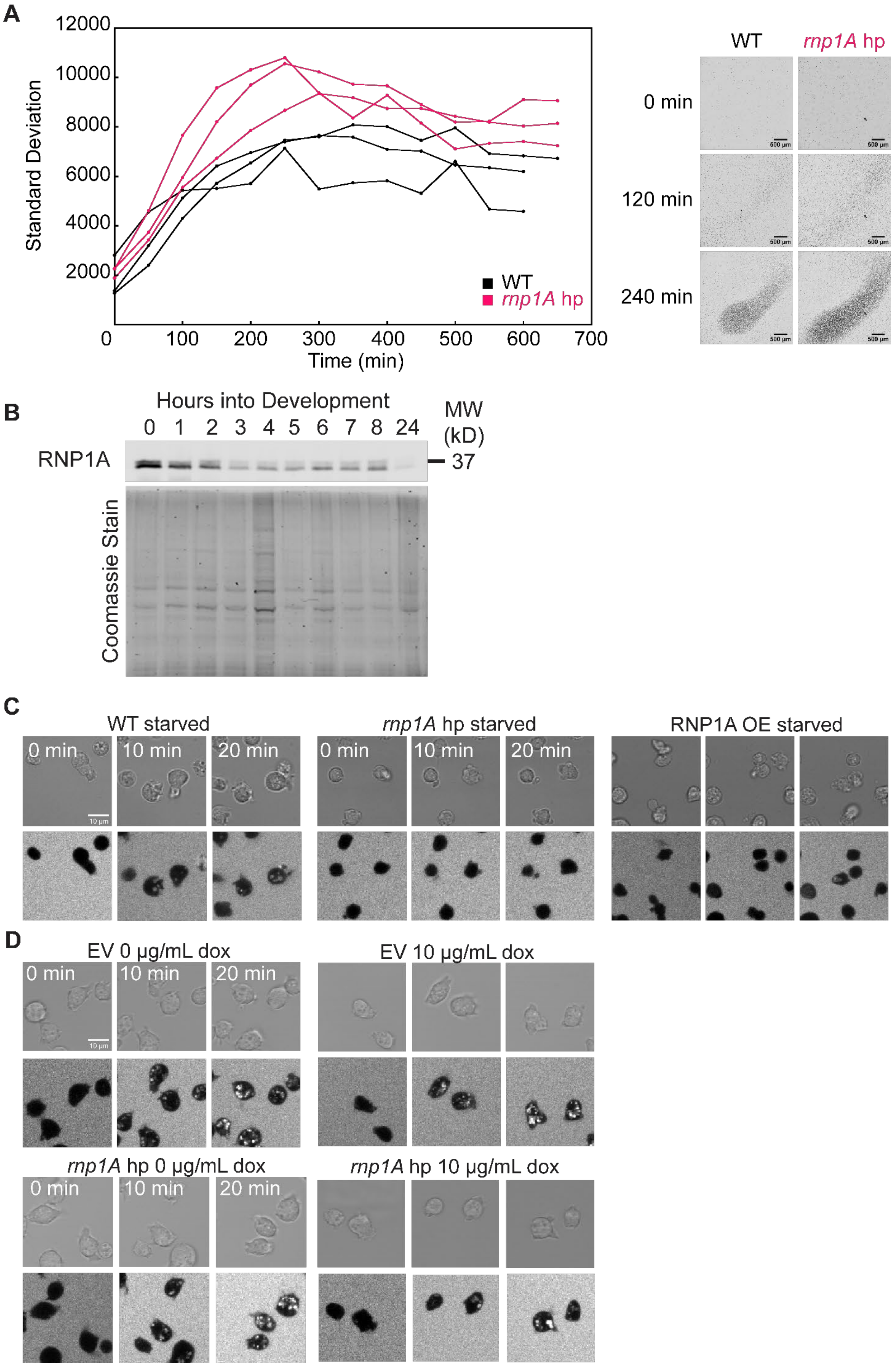

Journal of Cell Science • Supplementary information

Fig. S3. Starvation elicits different responses in *rnp1A* hp cells. (**A**) Aggregation of wild-type and *rnp1A* hp cells. Cells were seeded into MES starvation buffer and imaged every 5 minutes over 11 hours. Cells were imaged using brightfield, a 4x air objective with a Molecular Devices ImageXpress High-Content Imager. Cell aggregation was quantified by measuring each acquired frame's standard deviation using Fiji and plotting against time. Graph generated using KaleidaGraph. (**B**) Western analysis of RNP1A expression through development. WT (KAx3) cells were starved over 24 hours with hourly timepoints for the first 8 hours. Coomassie stain included below for protein loading control. (**C**, **D**) Images of TRITC-Dextran uptake in (**C**) starved wild type control, *rnp1A* knockdown and RNP1A-overexpressing cells, and in (**D**) doxycycline-inducible *rnp1A* knockdown cells over the span of 20 minutes. Scale bar, 10 µm, and applies to all images within the panel.

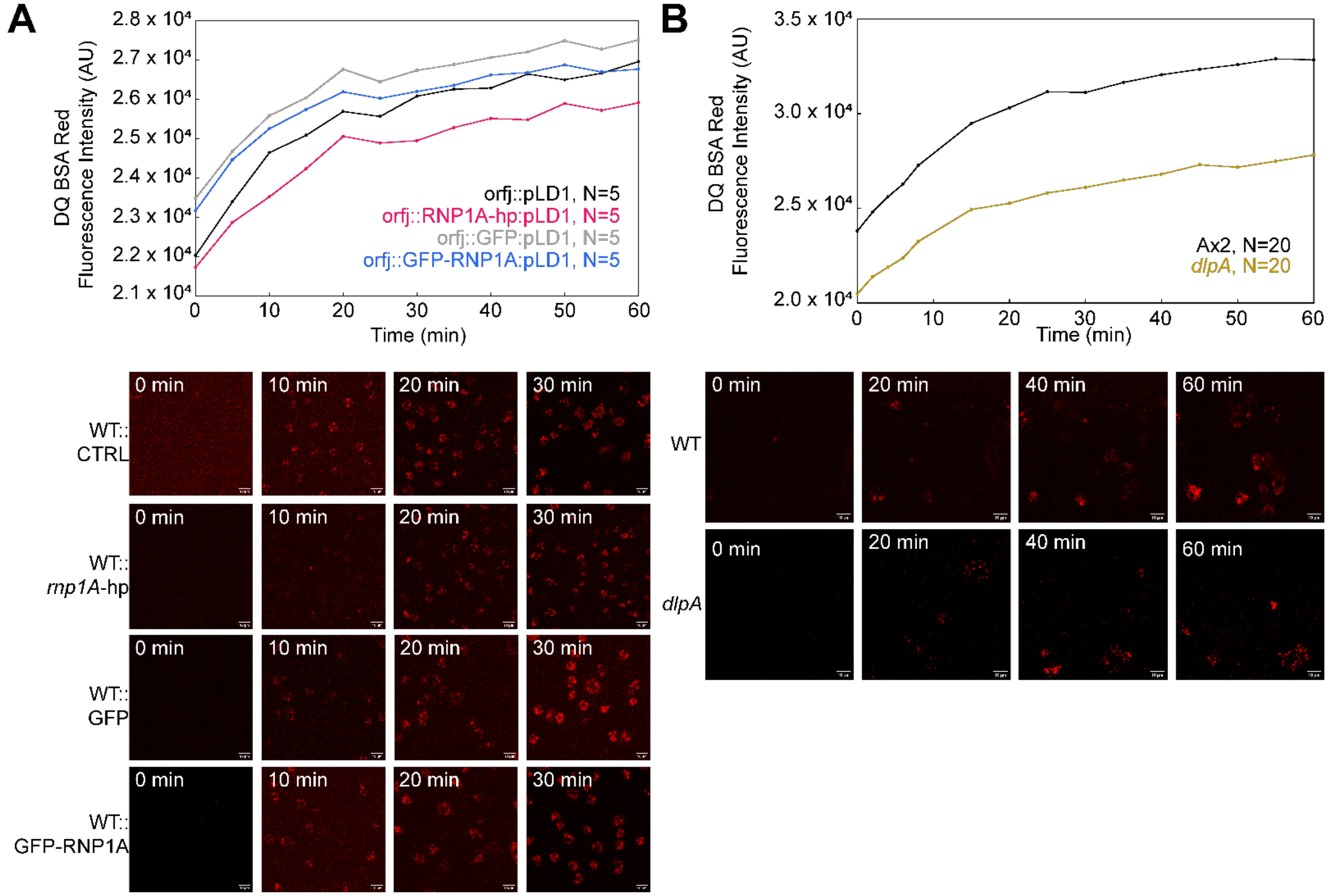

Fig. S4. *DlpA* KO and differential expression of RNP1A cells alter lysosomal activity. (**A, B) Top:** WT (orfJ) background (pLD1 empty vector; RNP1hp knockdown, GFP expression, and GFP-RNP1A) cells and WT(Ax2) and *dlpA* knockout cells were treated with DQ™ Red BSA to access lysosomal degradation kinetics. Transformants were incubated in DQ™ Red BSA and measured every 5 minutes for an hour with a microplate reader. (**A**) **Bottom:** Images show RNP1A transformants incubated with DQ™ Red BSA and imaged with confocal microscopy. (**B**) **Bottom:** Images show WT (Ax2) and *dlpA* null cells incubated with DQ™ Red BSA and imaged with confocal microscopy. Images were acquired on a Zeiss LSM 800 Confocal Light Laser Scanning Microscope, using a 63x Oil 1.4NA objective. Images were acquired every minute for an hour. Images were adjusted for visibility and modified with a scale bar using Fiji. Scale bars, 10 µm.

Journal of Cell Science • Supplementary information

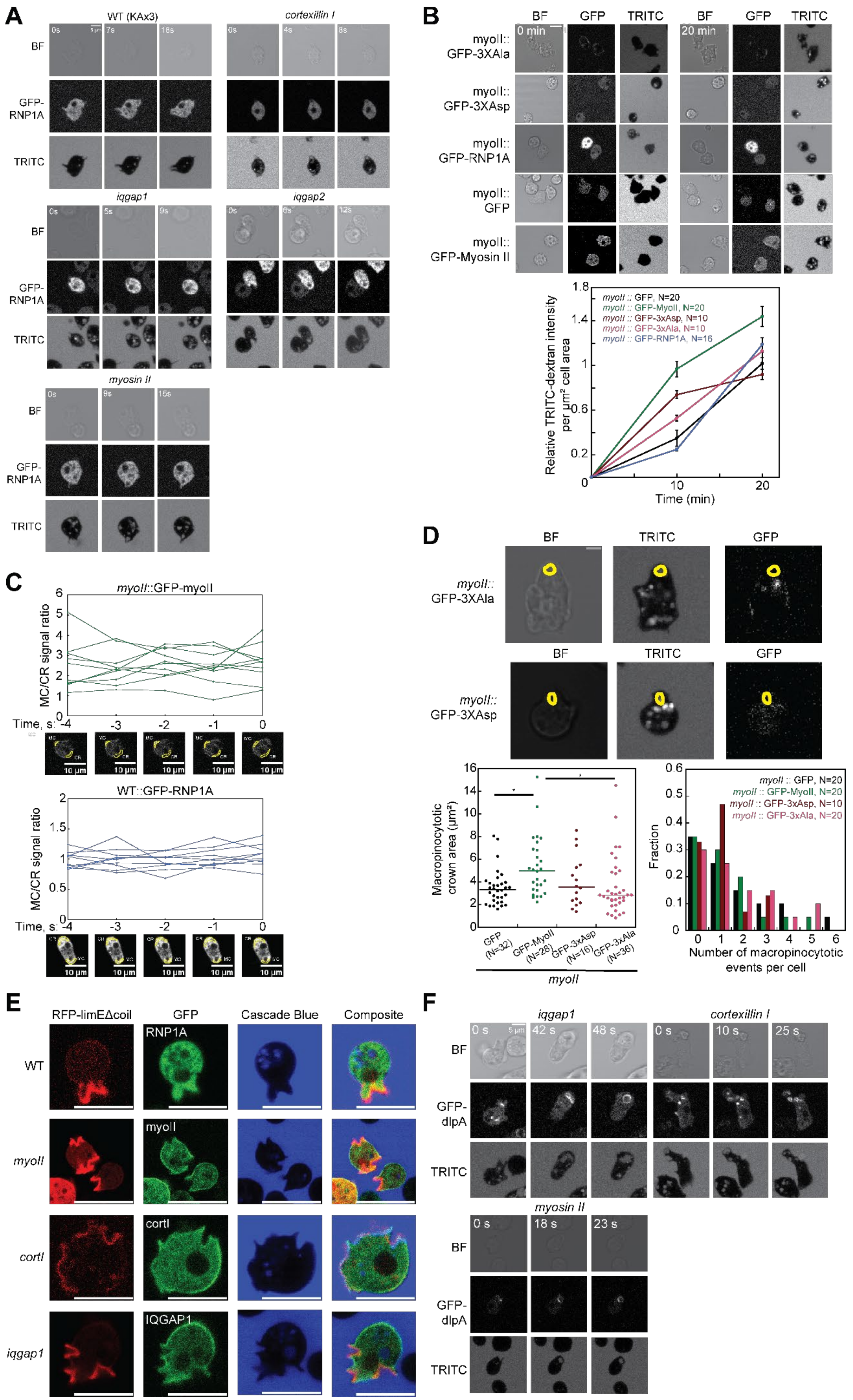

Journal of Cell Science • Supplementary information

Fig. S5. RNP1A distribution during macropinosome formation in Contractility Kit protein null mutants. (**A**) GFP-RNP1A distribution during macropinocytotic crown formation and closure in wild type (KAx3), *iqgap1* null, *iqgap2* null, *cortexillin I* null and *myosin II* null cells. Scale bar, 5 µm, and applies to corresponding panels. (**B**) TRITC-Dextran uptake in *myoII* null cells expressing GFP, GFP-Myosin II, GFP-3xAla, GFP-3XAsp, and GFP-RNP1A. TRITC-Dextran intensity was quantified by mean TRITC intensity of each cell, background subtracted, normalized to cell area, and then normalized to the first time point. Scale bar, 10 µm, and applies to all panels. Measurements were pooled from 20 GFP ctrl, 20 GFP-myoII, 10 GFP-3xAsp, 10 GFP-3xAla, and 16 GFP-RNP1A cells. Error bars indicate standard errors. (**C**) GFP-myoII and GFP-RNP1A mean signal ratio (MC/CR) of macropinosome cytoplasm/cortex (MC) divided by the mean signal of the cell rear cytoplasm/cortex (CR). Top graph provides the GFP-myosin II MC/CR ratio plotted against the last five frames (last 5 s) of the retracting macropinosome. Bottom graph indicates MC/CR ratio of GFP-RNP1A plotted against the last five frames (last 5 s) of macropinosome retraction. Example images of the last five frames are provided below the graphs, including the region of interests defining the MC and CR. (**D**) Quantification of macropinocytotic crown area and number of macropinocytotic events per cell over the span of 2 min. Average macropinocytotic events for GFP, *myoII* rescue, 3xAsp, and 3xAla are as follows: 1.5, 1.2, 0.95, and 1.7, respectively. Macropinocytotic crowns were manually traced as shown in yellow circles in images. Scale bar, 5 µm, and applies to all panels. Measurements were pooled from 20 GFP ctrl, 20 GFP-myoII, 15 GFP-3xAsp, and 20 GFP-3xAla cells. (**E**) Cascade Blue-Dextran uptake in *Ax3(Rep orf+)*, *myoII*, *cortI,* and *iqgap1* cells transformed with respective GFP-tagged rescue protein. Live cell images acquired by a Zeiss LSM 800 confocal microscope with a 63x oil 1.4NA objective. The images shown are representative of each cell line's population. Scale bars, 10 µm. (**F**) GFP-DlpA localization during macropinocytotic crown formation and closure in *iqgap1* null (Images representative of 3 repeats), *cortexillin I* null (Images representative of 9 repeats), and *myosin II* null cells (Images representative of 8 repeats). Scale bar, 5 µm, and applies to all panels. Statistical analysis was performed with Kruskal–Wallis followed by Wilcoxon-Mann–Whitney test. *, P≤0.05.

Journal of Cell Science • Supplementary information

Fig. S6. Source Data for Fig. 1 and Supplementary Fig. 2. Original Coomassie gel and western blot images that were used for analysis and data presentation in Fig. 1A and Sup. Fig. 2F. Yellow boxes indicate selected bands for data analysis and presentation, if other bands on the same blot were not used due to irrelevance, or unsuccessful transfer/blotting.

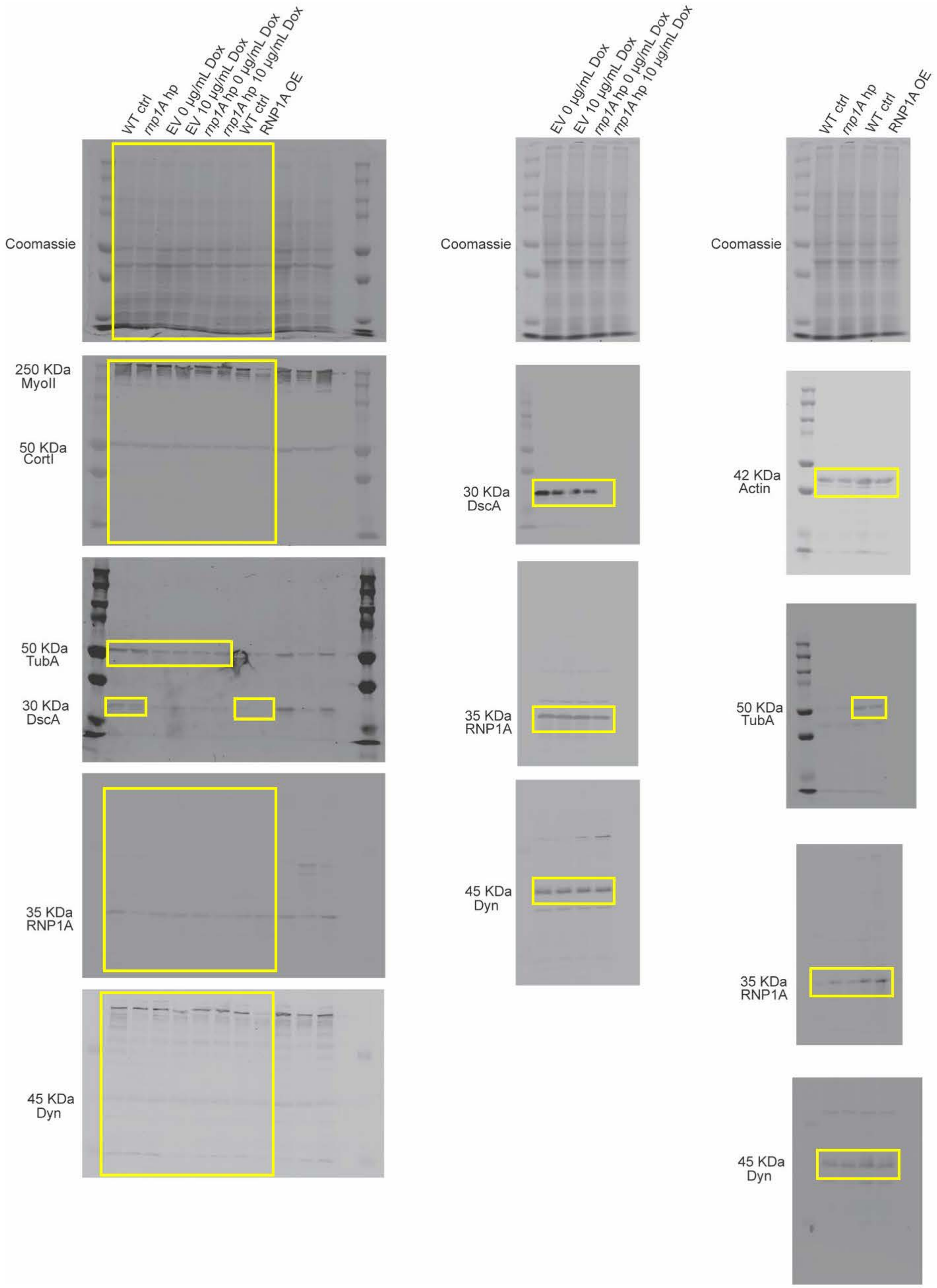

Journal of Cell Science • Supplementary information

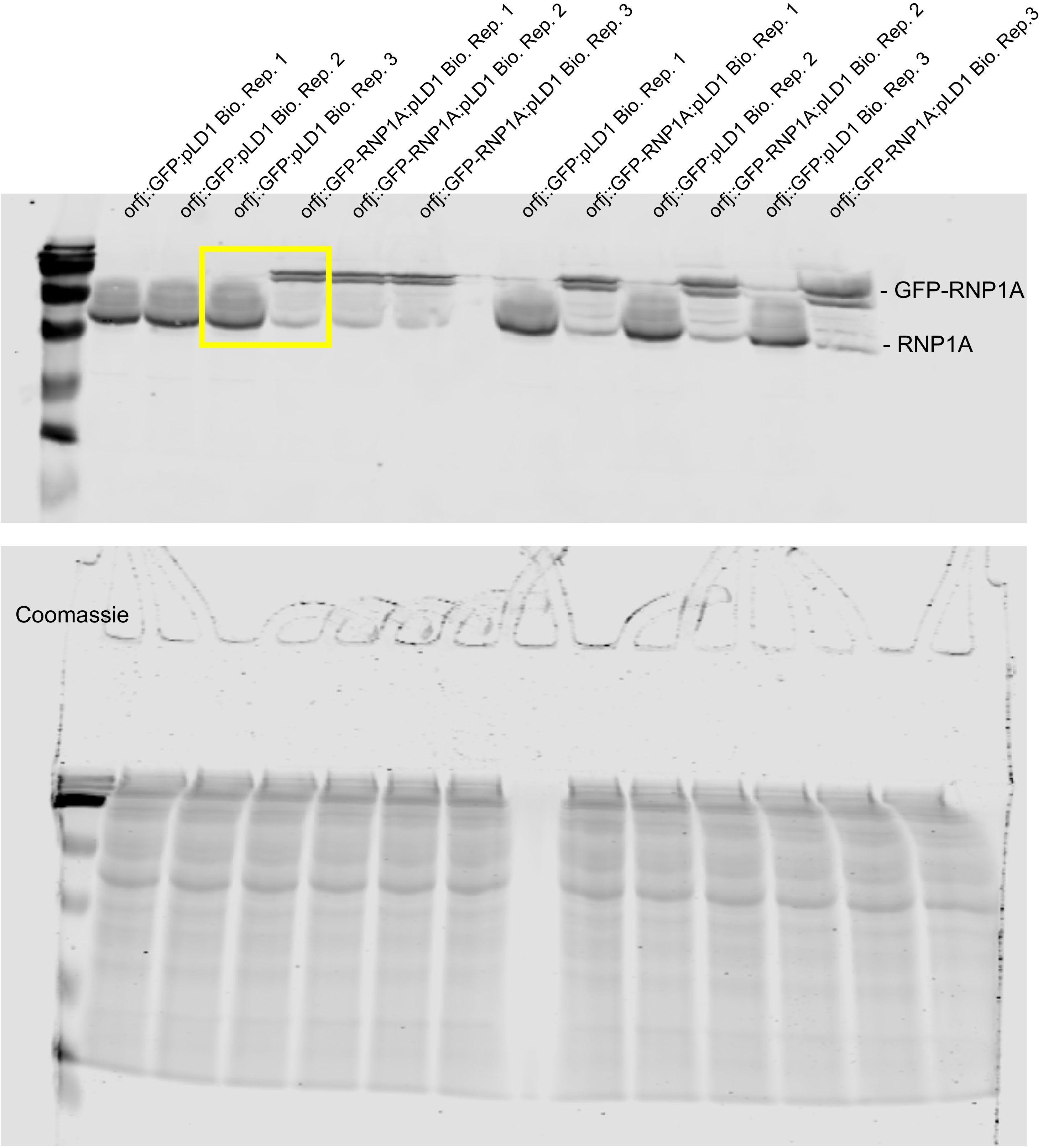

Source Data for Fig. 2A. Original western blot and Coomassie image that were used for analysis and data presentation in Fig. 2A. Yellow boxes indicate selected bands for presentation.

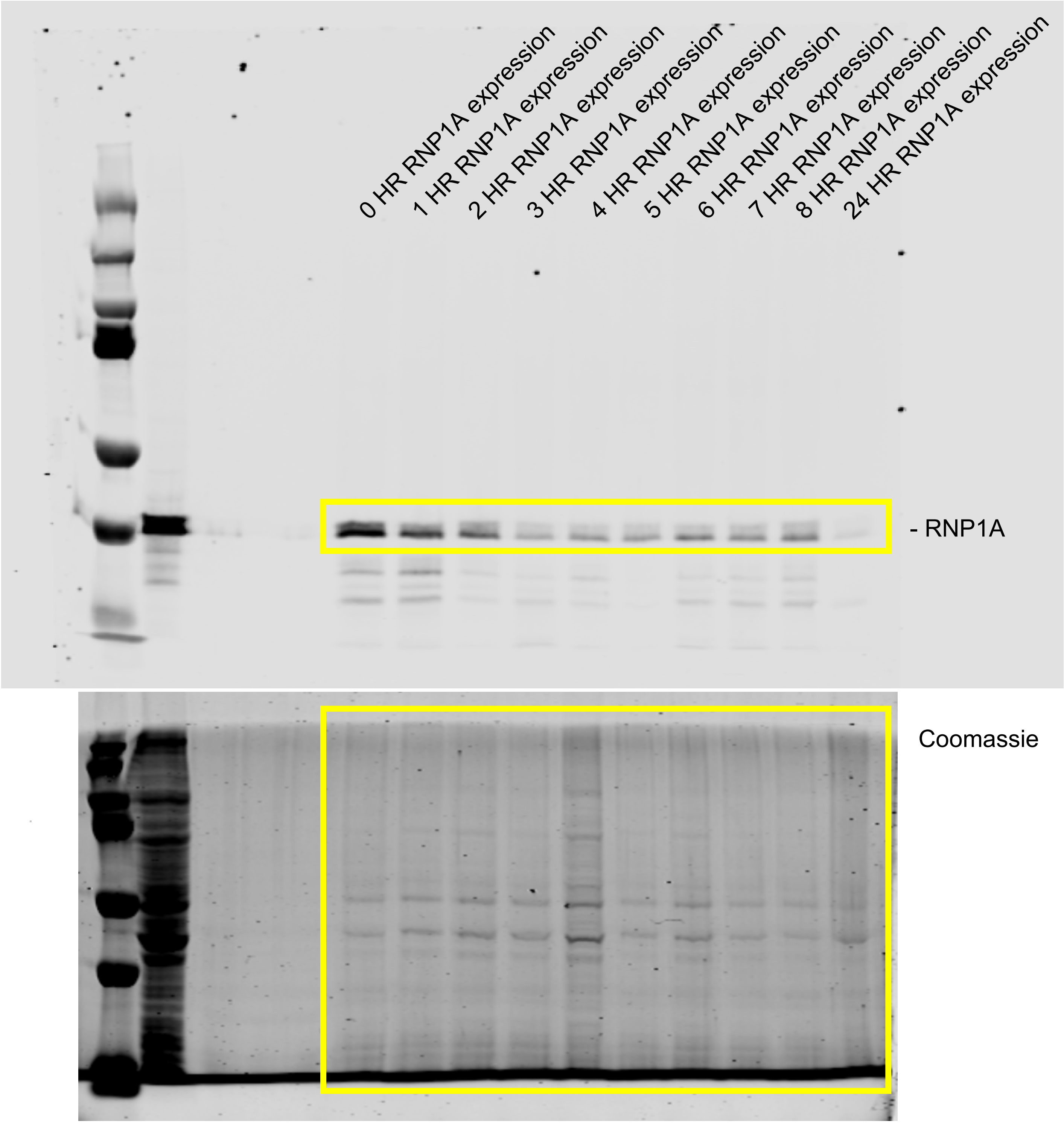

Source Data for Supplementary Fig. 3B. Original western blot and Coomassie image that were used for analysis and data presentation in Sup. Fig. 3. Yellow boxes indicate selected bands for presentation.

Fig. S6. Source Data for the western blots and Coomassie-stained gels.

Journal of Cell Science • Supplementary information

Table S1. List of genes that are significantly down-regulated from RNA-seq (R1).

DictyBase ID	log2FoldChange	padj	Gene Name	Gene Product
**DDB_G0273029**	-4.86309	0.020515	*DDB_G0273029*	-
**DDB_G0284167**	-4.53871	6.17E-08	*rnp1A*	RRM domain-containing protein RNP1A
**DDB_G0272030**	-4.40512	1.96E-11	*DDB_G0272030*	-
**DDB_G0272893**	-4.29112	3.64E-05	*rpl15-1*	S60 ribosomal protein L15
**DDB_G0285793**	-4.27707	6.55E-10	*cadA*	calcium-dependent cell adhesion molecule-1
**DDB_G0284549**	-3.77208	1.33E-07	*dduF*	speract/scavenger receptor domain-containing protein rare lipoprotein A domain-containing protein
**DDB_G0288219**	-3.74844	0.012893	*DDB_G0288219*	peptidase M66 family
**DDB_G0274093**	-3.51728	2.51E-05	*adrm1-2*	adhesion-regulating molecule family protein
**DDB_G0288879**	-3.43749	0.005595	*act11*	actin
**DDB_G0269134**	-3.35946	0.000871	*efaAI*	elongation factor 1 alpha elongation factor 1a
**DDB_G0273979**	-3.35692	0.029221	*psmD8-2*	26S proteasome regulatory subunit S14; 26S proteasome non-ATPase regulatory subunit 8
**DDB_G0293486**	-3.34721	4.57E-05	*DDB_G0293486_RTE*	TRE3-C ORF1
**DDB_G0274523**	-3.26298	0.017954	*DDB_G0274523*	RNA recognition motif-containing protein RRM
**DDB_G0272492**	-3.25398	0.006067	*DDB_G0272492*	-
**DDB_G0269234**	-3.25278	0.001236	*act8*	actin
**DDB_G0268600**	-3.09286	1.24E-06	*uduB*	-
**DDB_G0293818**	-3.08228	0.013056	*DDB_G0293818*	TRE3-C ORF2
**DDB_G0293482**	-3.07834	0.000871	*DDB_G0293482_RTE*	TRE3-C ORF2
**DDB_G0286057**	-3.07629	0.00146	*adk*	adenosine kinase ATP:adenosine 5'-phosphotransferase
**DDB_G0277345**	-3.05986	0.000943	*rps3a*	40S ribosomal protein S3a
**DDB_G0289779**	-3.0509	0.049518	*fmoA*	flavin-containing monooxygenase dimethylaniline monooxygenase [N-oxide-forming]
**DDB_G0275045**	-3.02097	0.000871	*gpbB*	guanine nucleotide-binding protein subunit beta-like protein
**DDB_G0273983**	-2.97093	0.007514	*rpl15-2*	S60 ribosomal protein L15
**DDB_G0267356**	-2.87855	0.001314	*DDB_G0267356_RTE*	Skipper GAG-PRO-POL
**DDB_G0267780**	-2.86491	0.024346	*DDB_G0267780_RTE*	TRE3-C ORF2
**DDB_G0291301**	-2.80427	0.048718	*DDB_G0291301*	amine oxidase (flavin-containing) putative sarcosine oxidase putative L-amino acid oxidase
**DDB_G0274113**	-2.80278	0.003873	*rpl8*	60S ribosomal protein L8
**DDB_G0267366**	-2.79972	0.002204	*DDB_G0267366_RTE*	Skipper GAG-PRO-POL
**DDB_G0267368**	-2.77418	0.002597	*DDB_G0267366_RTE*	Skipper GAG-PRO-POL
**DDB_G0289005**	-2.70803	0.014639	*act4*	actin
**DDB_G0270648**	-2.67362	0.042867	*DDB_G0270648_RTE*	TRE3-C ORF2
**DDB_G0288373**	-2.67221	0.013004	*efbA*	elongation factor 2
**DDB_G0272825**	-2.65533	0.006067	*rps4*	40S ribosomal protein S4
**DDB_G0290593**	-2.65343	0.001657	*DDB_G0290593*	ADF-H domain-containing protein
**DDB_G0270316**	-2.64554	0.006483	*rpsA*	40S ribosomal protein SA
**DDB_G0267418**	-2.62646	0.019447	*sahA*	adenosylhomocysteinase

Journal of Cell Science • Supplementary information

**DDB_G0291870**	-2.61677	0.00907	*rpl13*	S60 ribosomal protein L13
**DDB_G0293558**	-2.60866	0.011269	*pabpc1A*	RNA-binding region RNP-1 domain-containing protein<br>RNA recognition motif-containing protein RRM<br>polyadenylate binding protein (PABP)
**DDB_G0268580**	-2.59597	0.024666	*DDB_G0268580_RTE*	TRE3-C ORF2
**DDB_G0272520**	-2.569	0.046359	*act15*	actin
**DDB_G0269132**	-2.53335	0.048381	*ecmB*	extracellular matrix protein ST310
**DDB_G0291862**	-2.50209	0.010557	*rpl3*	60S ribosomal protein L3
**DDB_G0294144**	-2.50041	0.033772	*DDB_G0294144_RTE*	Skipper GAG-PRO
**DDB_G0292460**	-2.49655	0.010982	*rpl6*	60S ribosomal protein L6
**DDB_G0280823**	-2.49234	0.011981	*rps6*	40S ribosomal protein S6
**DDB_G0267360**	-2.46479	0.009884	-	-
**DDB_G0276969**	-2.44904	0.018166	*D2B*	type-B carboxylesterase/lipase family protein D2B
**DDB_G0293502**	-2.43015	0.014639	*rpl23a*	S60 ribosomal protein L23a
**DDB_G0277803**	-2.42961	0.019139	*rpl4*	60S ribosomal protein L4
**DDB_G0273067**	-2.37541	0.032876	*dscD-1*	discoidin I, delta chain
**DDB_G0286501**	-2.37499	0.021386	*rplP0*	ribosomal acidic phosphoprotein P0 60S acidic ribosomal protein P0
**DDB_G0282841**	-2.36918	0.044133	*DDB_G0282841*	-
**DDB_G0284047**	-2.3556	0.032668	*abcF2*	ABC transporter-related protein
**DDB_G0276203**	-2.35412	0.032668	*DDB_G0276203*	-
**DDB_G0277871**	-2.35353	0.029048	*rsc12*	-
**DDB_G0273887**	-2.34578	0.047798	*dscD-2*	discoidin I, delta chain
**DDB_G0273473**	-2.33979	0.022713	*DDB_G0273473*	VPS35 endosomal protein sorting factor-like protein
**DDB_G0285277**	-2.30669	0.024346	*rpl17*	S60 ribosomal protein L17
**DDB_G0291864**	-2.29914	0.019534	*rps8*	40S ribosomal protein S8
**DDB_G0275881**	-2.25555	0.019498	*rpl13a*	S60 ribosomal protein L13a
**DDB_G0280229**	-2.23763	0.033772	*rpl24*	S60 ribosomal protein L24
**DDB_G0267362**	-2.23519	0.02032	*DDB_G0267362_RTE*	Skipper GAG-PRO-POL
**DDB_G0276441**	-2.23378	0.02382	*rpl7*	S60 ribosomal protein L7
**DDB_G0286075**	-2.20988	0.027768	*rps5*	40S ribosomal protein S5
**DDB_G0284533**	-2.20696	0.022562	*rps13*	40S ribosomal protein S13
**DDB_G0288101**	-2.20617	0.021385	*rpl22*	S60 ribosomal protein L22
**DDB_G0278539**	-2.18033	0.032635	*rpl5*	60S ribosomal protein L5
**DDB_G0278429**	-2.16482	0.026067	*rps20*	40S ribosomal protein S20
**DDB_G0267470**	-2.16128	0.019498	*dicB*	discoidin-inducing complex (DIC) protein
**DDB_G0282379**	-2.15257	0.031617	*rpl28*	60S ribosomal protein L28
**DDB_G0293742**	-2.13509	0.033365	*rps2*	ribosomal protein S2
**DDB_G0271298**	-2.13423	0.01525	*rpl27*	S60 ribosomal protein L27
**DDB_G0271668**	-2.12509	0.026408	*rpl36*	S60 ribosomal protein L36
**DDB_G0293000**	-2.12313	0.042009	*rps3*	40S ribosomal protein S3
**DDB_G0279207**	-2.11211	0.047692	*rps19*	40S ribosomal protein S19
**DDB_G0284237**	-2.11112	0.035288	*rps12*	40S ribosomal protein S12
**DDB_G0285319**	-2.09363	0.007276	*H1*	histone H1 calmodulin-binding protein transcriptional repressor
**DDB_G0285893**	-2.08739	0.009008	*DDB_G0285893*	-
**DDB_G0282601**	-2.08275	0.044568	*rps11*	40S ribosomal protein S11

**DDB_G0289877**	-2.04933	0.040018	*rps9*	ribosomal protein 1024<br>40S ribosomal protein S9
**DDB_G0269850**	-2.04498	0.037278	*DDB_G0269850*	putative transmembrane protein<br>DG1041 family protein
**DDB_G0276457**	-2.0395	0.03017	*rps15a*	40S ribosomal protein S15a
**DDB_G0279189**	-2.03804	0.031971	*rpl11*	S60 ribosomal protein L11
**DDB_G0293522**	-2.01937	0.015432	*ponA*	ponticulin A
**DDB_G0283701**	-1.99413	0.032399	*guaB*	IMP dehydrogenase inosine-5'-<br>monophosphate dehydrogenase
**DDB_G0289025**	-1.97312	0.024666	*rps7*	40S ribosomal protein S7
**DDB_G0285617**	-1.92088	0.024592	*DDB_G0285617*	agglutinin domain-containing protein
**DDB_G0290545**	-1.88687	0.033365	*DDB_G0290545*	-
**DDB_G0281565**	-1.88233	0.042009	*rpl19*	S60 ribosomal protein L19
**DDB_G0279387**	-1.80576	0.042752	*rpl21*	S60 ribosomal protein L21

Table S2. List of genes that are significantly up-regulated from RNA-seq (R1).

DictyBase ID	log2FoldChange	padj	Gene Name	Gene Product
**DDB_G0283425**	1.789626	0.044351	DDB_G0283425	-
**DDB_G0289897**	1.800242	0.040018	DDB_G0289897	-
**DDB_G0289537**	1.896813	0.047026	DDB_G0289537	-
**DDB_G0285309**	1.909575	0.024666	DDB_G0285309	-
**DDB_G0279637**	1.914916	0.016043	DDB_G0279637	-
**DDB_G0276261**	1.946131	0.029365	mcfV	-
**DDB_G0292926**	1.961666	0.015552	iliQ	ankyrin repeat-containing protein
**DDB_G0268566**	1.991482	0.024666	DDB_G0268566	-
**DDB_G0267906**	1.998436	0.013813	DDB_G0267906	OTU domain containin protein
**DDB_G0285065**	2.056134	0.040044	DDB_G0285065	S-adenosyl-L-methionine-dependent methyltransferase family protein
**DDB_G0283923**	2.085331	0.014078	DDB_G0283923	-
**DDB_G0285975**	2.103048	0.007276	DDB_G0285975	-
**DDB_G0271916**	2.109911	0.048293	rtoA	-
**DDB_G0267930**	2.110097	0.033772	DDB_G0267930	-
**DDB_G0291694**	2.115819	0.008868	DDB_G0291694	SEC23-interacting protein
**DDB_G0281617**	2.126882	0.032309	DDB_G0281617	-
**DDB_G0293102**	2.179938	0.006067	DDB_G0293102	-
**DDB_G0275521**	2.188116	0.031726	DDB_G0275521	-
**DDB_G0284637**	2.20646	0.014115	DDB_G0284637	-
**DDB_G0282467**	2.21204	0.033772	DDB_G0282467	1-aminocyclopropane-1-carboxylate synthase<br>S-adenosyl-L-methionine methylthioadenosine-lyase<br>ACC synthase
**DDB_G0282715**	2.216831	0.008391	DDB_G0282715	Neutral and basic amino acid transport protein rBAT
**DDB_G0279721**	2.237804	0.013004	ubqH	ubiquitin H
**DDB_G0289519**	2.237857	0.004146	DDB_G0289519	-
**DDB_G0268006**	2.238722	0.031638	DDB_G0268006	UspA domain-containing protein
**DDB_G0283639**	2.264347	0.031602	DDB_G0283639	-
**DDB_G0272280**	2.309208	0.011425	DDB_G0272280	AhpC/TSA family protein
**DDB_G0269482**	2.328099	0.035288	DDB_G0269482	type A von Willebrand factor (VWFA) domain-containing protein
**DDB_G0293202**	2.328641	0.039756	trafH	TNF receptor-associated factor H
**DDB_G0269826**	2.334473	0.014331	DDB_G0269826	-
**DDB_G0270636**	2.337123	0.015206	DDB_G0270636	transmembrane protein
**DDB_G0292696**	2.348204	0.003873	colA	colossin A
**DDB_G0287083**	2.358398	0.002204	DDB_G0287083	-
**DDB_G0272843**	2.370275	0.042867	DDB_G0272843	-
**DDB_G0276271**	2.383757	0.022562	DDB_G0276271	-
**DDB_G0288497**	2.385965	0.005595	DDB_G0288497	-
**DDB_G0275145**	2.394288	0.015145	DDB_G0275145	RING zinc finger-containing protein IBR zinc finger-containing protein
**DDB_G0289799**	2.436997	0.019984	DDB_G0289799_ps	-
**DDB_G0272218**	2.454299	0.007514	DDB_G0272218	-
**DDB_G0290957**	2.468255	0.006067	cprA	cysteine proteinase 1
**DDB_G0280461**	2.482727	0.048141	DDB_G0280461	LISK family protein kinase protein kinase, TKL group tyrosine kinase-like protein

**DDB_G0290959**	2.485412	0.033365	chtC	cheater C
**DDB_G0274369**	2.495904	0.027768	DDB_G0274369	-
**DDB_G0280051**	2.497141	0.000871	DDB_G0280051	-
**DDB_G0275661**	2.502425	0.013813	DDB_G0275661	-
**DDB_G0267238**	2.515416	0.048718	DDB_G0267238_RTE	DIRS1 ORF1
**DDB_G0271134**	2.541676	0.001329	celA	cellulase 270-6
**DDB_G0274291**	2.578245	0.001685	lyT2-4	putative T4-like lysozyme 2
**DDB_G0277599**	2.578535	0.011425	DDB_G0277599	-
**DDB_G0285215**	2.62068	0.005131	DDB_G0285215	-
**DDB_G0287313**	2.661185	0.037814	DDB_G0287313	-
**DDB_G0290993**	2.666607	0.031289	DDB_G0290993	-
**DDB_G0283911**	2.669408	0.021385	hsp69	heat shock protein 69
**DDB_G0286835**	2.691517	0.005158	DDB_G0286835	-
**DDB_G0279247**	2.699428	0.04671	DDB_G0279247	cyclin-like protein
**DDB_G0293542**	2.719405	0.008609	DDB_G0293542	-
**DDB_G0275487**	2.719763	0.042529	DDB_G0275487	-
**DDB_G0268322**	2.745789	0.011981	DDB_G0268322	zinc-containing alcohol dehydrogenase (ADH)
**DDB_G0271218**	2.748004	0.031716	DDB_G0271218	-
**DDB_G0269112**	2.764599	0.001035	celB	cellulose-binding protein
**DDB_G0290377**	2.777114	0.000559	agnB	argonaut-like protein
**DDB_G0285615**	2.780971	0.010097	iliA	-
**DDB_G0289163**	2.820033	0.004236	DDB_G0289163	-
**DDB_G0276697**	2.822673	0.003547	DDB_G0276697	-
**DDB_G0287673**	2.823251	0.006067	DDB_G0287673	-
**DDB_G0285917**	2.836112	0.005588	DDB_G0285917	-
**DDB_G0285289**	2.843348	0.003873	spoB	spore-specific protein B
**DDB_G0274701**	2.863982	0.003838	DDB_G0274701	-
**DDB_G0289473**	2.870866	0.000182	DDB_G0289473	P-type ATPase<br>Ca2+-ATPase
**DDB_G0275691**	2.907466	0.04961	DDB_G0275691	-
**DDB_G0290637**	2.92128	0.034491	DDB_G0290637	-
**DDB_G0268874**	2.931953	0.010557	DDB_G0268874	-
**DDB_G0275133**	2.944104	0.024666	DDB_G0275133	-
**DDB_G0294515**	2.950333	0.000377	dutA	structural RNA
**DDB_G0291470**	2.967298	0.00907	DDB_G0291470	-
**DDB_G0289411**	2.979079	0.000303	DDB_G0289411	-
**DDB_G0275163**	3.008874	0.001755	DDB_G0275163	-
**DDB_G0280919**	3.019001	3.63E-05	DDB_G0280919	-
**DDB_G0275033**	3.022329	0.012458	DDB_G0275033	type A von Willebrand factor (VWFA) domain-containing protein
**DDB_G0280503**	3.026459	0.010978	DDB_G0280503	type A von Willebrand factor (VWFA) domain-containing protein
**DDB_G0292334**	3.047546	0.010272	DDB_G0292334	AIG1-type guanine nucleotide-binding domain-containing protein
**DDB_G0267210**	3.079572	0.045681	DDB_G0267210_RTE	DIRS1 ORF2 fragment
**DDB_G0275319**	3.084532	0.001956	DDB_G0275319	-
**DDB_G0289039**	3.120849	0.002711	DDB_G0289039	trypsin-like serine proteases family protein<br>sterile alpha motif homology domain-containing protein

Journal of Cell Science • Supplementary information

**DDB_G0293364**	3.12152	1.38E-05	sigB	peptidase M8, leishmanolysin family protein
**DDB_G0268318**	3.124346	0.001314	DDB_G0268318	-
**DDB_G0289787**	3.125172	0.003838	DDB_G0289787	-
**DDB_G0288945**	3.12668	0.014639	DDB_G0288945	-
**DDB_G0281915**	3.142497	0.044582	DDB_G0281915	transmembrane protein
**DDB_G0280615**	3.179997	0.002544	DDB_G0280615	-
**DDB_G0282059**	3.182622	0.0049	DDB_G0282059	-
**DDB_G0285833**	3.186381	0.035813	DDB_G0285833	-
**DDB_G0291742**	3.19688	0.005819	DDB_G0291742_ps	-
**DDB_G0291646**	3.20089	0.002273	DDB_G0291646	-
**DDB_G0269044**	3.217057	0.045498	DDB_G0269044	-
**DDB_G0276219**	3.219538	0.005588	DDB_G0276219	putative transmembrane protein
**DDB_G0281087**	3.251481	0.001027	gtaV	putative GATA-binding transcription factor GATA zinc finger domain-containing protein 22
**DDB_G0288709**	3.280116	0.003385	tgrM2	IPT/TIG domain-containing protein immunoglobulin E-set domain-containing protein
**DDB_G0292996**	3.309328	0.028989	rasD	Ras GTPase RasD
**DDB_G0276747**	3.313621	0.000871	DDB_G0276747	-
**DDB_G0292462**	3.331333	0.001236	DDB_G0292462	-
**DDB_G0279483**	3.33156	0.000303	pldB	phospholipase D1
**DDB_G0291223**	3.33915	0.007146	DDB_G0291223_RTE	DIRS1 ORF2/ORF3 fusion fragment
**DDB_G0272506**	3.366272	0.029187	DDB_G0272506	-
**DDB_G0273981**	3.39256	0.002817	dpm2-2	dolichyl-phosphate mannosyltransferase 2 regulatory subunit
**DDB_G0267258**	3.428489	0.000374	DDB_G0267258_RTE	DIRS1 ORF2 fragment
**DDB_G0287581**	3.474005	6.08E-07	DDB_G0287581	-
**DDB_G0284345**	3.480206	0.002525	CYP556A1	cytochrome P450 family protein
**DDB_G0295769**	3.517811	0.005157	DDB_G0295769	-
**DDB_G0287311**	3.553997	0.024666	DDB_G0287311_ps	-
**DDB_G0290969**	3.606855	0.024666	DDB_G0290969	-
**DDB_G0291420**	3.632364	0.008288	DDB_G0291420	-
**DDB_G0268212**	3.653593	1.55E-07	DDB_G0268212	putative DEAD/DEAH box helicase
**DDB_G0273467**	3.669025	0.031148	trappc1-1	trafficking protein particle complex subunit 1
**DDB_G0281607**	3.671525	0.005588	DDB_G0281607	-
**DDB_G0271314**	3.69189	0.000871	iliH	Endo-1,4-beta-glucanase family protein glycoside hydrolase family 9 protein
**DDB_G0271068**	3.732836	6.77E-07	iliC	armadillo-type fold-containing protein
**DDB_G0288791**	3.767972	0.044025	hlcs1	biotin--[acetyl-CoA-carboxylase] ligase 1
**DDB_G0275197**	3.778383	0.013849	CYP518B1	cytochrome P450 family protein
**DDB_G0274391**	3.781804	0.006067	alfA	alpha-L-fucosidase
**DDB_G0276705**	3.875559	8.85E-06	DDB_G0276705	-
**DDB_G0287659**	3.891734	3.94E-06	nagD	beta-N-acetylhexosaminidase glycoside hydrolase family 20 protein beta-hexosaminidase N-acetylglucosaminidase
**DDB_G0275975**	3.891893	0.048462	DDB_G0275975	-
**DDB_G0274707**	3.909362	0.019139	gpt5	putative glycophosphotransferase
**DDB_G0278231**	3.933854	0.005588	DDB_G0278231	-
**DDB_G0269254**	3.967805	4.91E-08	sigJ	transmembrane protein SigJ

**DDB_G0285883**	3.972244	0.033365	DDB_G0285885_ps	-
**DDB_G0268836**	4.052793	0.000871	DDB_G0268836	-
**DDB_G0285885**	4.055654	0.005602	DDB_G0285885_ps	-
**DDB_G0290435**	4.076787	4.12E-07	DDB_G0290435	-
**DDB_G0291041**	4.12055	0.00542	DDB_G0291041_ps	-
**DDB_G0285103**	4.157724	3.94E-07	DDB_G0285103	-
**DDB_G0276729**	4.234143	0.000135	DDB_G0276729	-
**DDB_G0289693**	4.248957	0.000389	DDB_G0289693	-
**DDB_G0285723**	4.36357	4.91E-08	DDB_G0285723	-
**DDB_G0291422**	4.378743	0.000871	DDB_G0291422	-
**DDB_G0272214**	4.624264	0.011921	DDB_G0272214	-
**DDB_G0274025**	4.674127	0.008288	DDB_G0274025	DUF1794 family protein
**DDB_G0283867**	4.806388	5.13E-10	cprC	cysteine proteinase 3
**DDB_G0267792**	4.820973	3.61E-12	DDB_G0267792	-
**DDB_G0273927**	4.895728	0.002519	DDB_G0273927	-
**DDB_G0273477**	4.912983	0.039756	DDB_G0273477	-
**DDB_G0285983**	5.050155	1.78E-07	lsr2	long serine homopolymer repeat protein 2
**DDB_G0268784**	5.053553	1.85E-06	DDB_G0268784	major facilitator superfamily 1 transporter (MFS-1)
**DDB_G0280761**	5.090664	0.040018	DDB_G0280761	putative sodium-dependent phosphate transporter
**DDB_G0284843**	5.216132	4.91E-08	DDB_G0284843	transmembrane protein
**DDB_G0276469**	5.562623	1.78E-07	syn16B	t-SNARE family protein<br>putative syntaxin 16
**DDB_G0276407**	5.683334	1.33E-07	DDB_G0276407	adenylate kinase
**DDB_G0282329**	5.871956	0.000898	DDB_G0282329	-
**DDB_G0287037**	6.062084	0.016833	DDB_G0287037	Protein SDS23
**DDB_G0273089**	6.391395	0.001423	coq10-1	putative coenzyme Q-binding protein
**DDB_G0282331**	7.214023	0.005377	DDB_G0282331	-
**DDB_G0274699**	7.412743	0.004364	wfdc	WAP four-disulfide core domain protein
**DDB_G0280915**	7.492754	0.003174	DDB_G0280915	-

Journal of Cell Science • Supplementary information

Table S3. List of genes that are significantly down-regulated from RNA-seq (R2).

DictyBase ID	log2FoldChange	padj	Gene Name	Gene Product
**DDB_G0273121**	-4.5229	0.000369	pakH-1	STE20 family protein kinase PakH
**DDB_G0284295**	-3.80139	1.65E-13	iliG	Endo-1,4-beta-glucanase family protein<br>glycoside hydrolase family 9 protein
**DDB_G0280649**	-3.7005	0.000163	DDB_G0280649	-
**DDB_G0267780**	-3.46671	0.001607	DDB_G0267780_RTE	TRE3-C ORF2
**DDB_G0285043**	-3.27997	0.007796	DDB_G0285043	-
**DDB_G0272242**	-3.16691	0.025419	cupI	ricin B lectin domain-containing protein<br>cup family protein
**DDB_G0282225**	-3.13298	0.040912	DDB_G0282225	-
**DDB_G0284167**	-3.00265	0.002432	rnp1A	RRM domain-containing protein RNP1A
**DDB_G0281399**	-2.89139	0.001637	DDB_G0281399	putative FAD-binding oxidoreductase
**DDB_G0284925**	-2.43535	0.000107	DDB_G0284925	-
**DDB_G0272030**	-2.40633	0.010326	DDB_G0272030	-
**DDB_G0289497**	-2.18473	0.000675	DDB_G0289497	-
**DDB_G0271692**	-2.1749	0.000576	comC	EGF-like domain-containing protein
**DDB_G0284931**	-2.17376	0.002432	DDB_G0284931	-
**DDB_G0289863**	-2.02739	0.037595	DDB_G0289863	-
**DDB_G0277383**	-1.97755	0.043463	DDB_G0277383	-
**DDB_G0285939**	-1.85917	0.007796	vinA	alpha-catenin related protein
**DDB_G0274727**	-1.7916	0.003609	act19	actin
**DDB_G0292028**	-1.74423	0.001637	DDB_G0292028	von Willebrand factor A domain-containing protein 5B1
**DDB_G0274425**	-1.67468	0.037579	DDB_G0274425	protein phosphatase 2C-related protein
**DDB_G0289629**	-1.66966	0.00857	DDB_G0289629	-
**DDB_G0279717**	-1.55108	0.036458	DDB_G0279717	carboxylesterase, type B family protein
**DDB_G0291796**	-1.4707	0.037595	araA	putative regulator of adhesion and motility 4
**DDB_G0269850**	-1.43388	0.028624	DDB_G0269850	putative transmembrane protein DG1041 family protein

Table S4. List of genes that are significantly up-regulated from RNA-seq (R2).

DictyBase ID	log2FoldChange	padj	Gene Name	Gene Product
**DDB_G0277023**	1.757552	0.033877	DDB_G0277023	-
**DDB_G0284535**	1.829913	0.002163	CYP508A4	cytochrome P450 family protein
**DDB_G0274115**	2.143325	0.009711	abcG12	ABC transporter G family protein
**DDB_G0283483**	2.452739	0.037579	srfD	putative MADS-box transcription factor
**DDB_G0276705**	2.745867	0.000452	DDB_G0276705	-
**DDB_G0270540**	2.774057	0.037595	DDB_G0270540	-
**DDB_G0295807**	2.846629	0.037595	DDB_G0295807	-
**DDB_G0287583**	3.128894	0.037595	gerC	spore germination protein C
**DDB_G0278953**	3.13045	0.007318	DDB_G0278953	CBS (cystathionine-beta-synthase) domain-containing protein
**DDB_G0286239**	3.138633	0.010326	DDB_G0286239	alpha/beta hydrolase fold-1 domain-containing protein serine hydrolase-like protein
**DDB_G0289723**	3.489629	0.005321	abhd	alpha/beta hydrolase fold-1 domain-containing protein abhydrolase domain-containing protein
**DDB_G0284511**	3.509998	0.017282	DDB_G0284511	-
**DDB_G0273073**	3.530612	0.037595	abcG17-1	ABC transporter G family protein
**DDB_G0283281**	3.585047	0.007318	DDB_G0283281	Strictosidine synthase family protein
**DDB_G0283649**	3.71777	0.037595	DDB_G0283649	NADPH-dependent FMN reductase family protein putative FMN-dependent NAD(P)H:quinone reductase
**DDB_G0278219**	3.829192	0.001003	DDB_G0278219	-
**DDB_G0290993**	4.028647	6.25E-13	DDB_G0290993	-
**DDB_G0268160**	4.225233	0.031858	gnt12	putative glycosyltransferase putative GlcNAc transferase putative beta-1,3 N-acetylglucosaminyltransferase
**DDB_G0282353**	4.44043	0.038561	CYP513E1	cytochrome P450 family protein
**DDB_G0292462**	4.745043	0.001003	DDB_G0292462	-
**DDB_G0283213**	5.771081	0.000208	DDB_G0283213	-
**DDB_G0281361**	6.47573	0.000163	DDB_G0281361	-

Journal of Cell Science • Supplementary information

Table S5. List of genes that are significantly down- and up-regulated from mRNA-seq (R1 and R2), which align with statistically significant genes associated with development described by Kin et al 2018.

Gene ID	Gene Name	Gene Product	Fold-Change during development	Found in R1 data set?	Fold-change	Adjusted p-value	Found in R2 data set?	Fold-change	Adjusted p-value
**DDB_G0284167**	rnp1A	RRM domain-containing protein RNP1A	-3.58	Yes	-4.54	6.17E-08	Yes	-3.00	0.00243
**DDB_G0272030**	DDB_G0272030	-	-2.76	Yes	-4.41	1.96E-11	Yes	-2.41	0.0103
**DDB_G0288219**	DDB_G0288219	peptidase M66 family	-2.94	Yes	-3.75	1.29E-02	No	-	-
**DDB_G0288879**	act11	actin	-3.74	Yes	-3.44	5.60E-03	No	-	-
**DDB_G0269134**	efaAI	elongation factor 1 alpha elongation factor 1a	-3.08	Yes	-3.36	8.71E-04	No	-	-
**DDB_G0268600**	uduB	-	-3.68	Yes	-3.09	1.24E-06	No	-	-
**DDB_G0286057**	adk	adenosine kinase ATP:adenosine 5'-phosphotransferase	-2.88	Yes	-3.08	1.46E-03	No	-	-
**DDB_G0289779**	fmoA	flavin-containing monooxygenase	-2.88	Yes	-3.05	4.95E-02	No	-	-
**DDB_G0289005**	act4	actin	-2.79	Yes	-2.71	1.46E-02	No	-	-
**DDB_G0288373**	efbA	elongation factor 2	-2.17	Yes	-2.67	1.30E-02	No	-	-
**DDB_G0290593**	DDB_G0290593	ADF-H domain-containing protein	-3.51	Yes	-2.65	1.66E-03	No	-	-
**DDB_G0267418**	sahA	adenosylhomocysteinase	-2.13	Yes	-2.63	1.94E-02	No	-	-
**DDB_G0293558**	pabpc1A	RNA-binding region RNP-1 domain-containing protein	-1.54	Yes	-2.61	1.13E-02	No	-	-
**DDB_G0272520**	act15	actin	-2.27	Yes	-2.57	4.64E-02	No	-	-
**DDB_G0269132**	ecmB	extracellular matrix protein ST310	-2.60	Yes	-2.53	4.84E-02	No	-	-
**DDB_G0294144**	DDB_G0294144_RTE	Skipper GAG-PRO	-1.43	Yes	-2.50	3.38E-02	No	-	-
**DDB_G0286501**	rplP0	ribosomal acidic phosphoprotein P0 60S acidic ribosomal protein P0	-1.72	Yes	-2.37	2.14E-02	No	-	-
**DDB_G0277871**	rsc12	-	-2.97	Yes	-2.35	2.90E-02	No	-	-
**DDB_G0285277**	rpl17	S60 ribosomal protein L17	-1.94	Yes	-2.31	2.43E-02	No	-	-
**DDB_G0267362**	DDB_G0267362_RTE	Skipper GAG-PRO-POL	-2.04	Yes	-2.24	2.03E-02	No	-	-
**DDB_G0282379**	rpl28	60S ribosomal protein L28	-1.94	Yes	-2.15	3.16E-02	No	-	-
**DDB_G0293742**	rps2	ribosomal protein S2	-1.54	Yes	-2.14	3.34E-02	No	-	-
**DDB_G0285319**	H1	histone H1 calmodulin-binding protein transcriptional repressor	-3.97	Yes	-2.09	7.28E-03	No	-	-
**DDB_G0279189**	rpl11	S60 ribosomal protein L11	-2.04	Yes	-2.04	3.20E-02	No	-	-
**DDB_G0279387**	rpl21	S60 ribosomal protein L21	-3.76	Yes	-1.81	4.28E-02	No	-	-
**DDB_G0284295**	iliG	Endo-1,4-beta-glucanase family protein glycoside hydrolase family 9 protein	-8.04	No	-	-	Yes	-3.80	1.65E-13
**DDB_G0272242**	cupI	ricin B lectin domain-containing protein cup family protein	-4.93	No	-	-	Yes	-3.17	0.0254
**DDB_G0289497**	DDB_G0289497	-	-2.74	No	-	-	Yes	-2.18	0.000675
**DDB_G0289863**	DDB_G0289863	-	-4.79	No	-	-	Yes	-2.03	0.0376
**DDB_G0277383**	DDB_G0277383	-	-1.83	No	-	-	Yes	-1.98	0.0435
**DDB_G0285939**	vinA	alpha-catenin related protein	-2.48	No	-	-	Yes	-1.86	0.00780
**DDB_G0274727**	act19	actin	-3.29	No	-	-	Yes	-1.79	0.00361
**DDB_G0292028**	DDB_G0292028	von Willebrand factor A domain-containing protein 5B1	-1.93	No	-	-	Yes	-1.74	0.00164
**DDB_G0274425**	DDB_G0274425	protein phosphatase 2C-related protein	-3.20	No	-	-	Yes	-1.67	0.0376
**DDB_G0279717**	DDB_G0279717	carboxylesterase, type B family protein	-2.54	No	-	-	Yes	-1.55	0.0365
**DDB_G0292462**	DDB_G0292462	-	7.95	Yes	3.33	0.00124	Yes	4.75	0.001003
**DDB_G0276705**	DDB_G0276705	-	2.24	Yes	3.88	8.85E-06	Yes	2.75	0.000452
**DDB_G0283425**	DDB_G0283425	-	5.40	Yes	1.79	0.04435	No	-	-
**DDB_G0279637**	DDB_G0279637	-	3.82	Yes	1.91	0.01604	No	-	-
**DDB_G0276261**	mcfV	-	1.88	Yes	1.95	0.02937	No	-	-
**DDB_G0268566**	DDB_G0268566	-	2.53	Yes	1.99	0.02467	No	-	-
**DDB_G0267906**	DDB_G0267906	OTU domain containing protein	3.16	Yes	2.00	0.01381	No	-	-
**DDB_G0285065**	DDB_G0285065	S-adenosyl-L-methionine-dependent methyltransferase family protein	3.09	Yes	2.06	0.04004	No	-	-
**DDB_G0285975**	DDB_G0285975	-	3.92	Yes	2.10	0.00728	No	-	-
**DDB_G0271916**	rtoA	-	2.23	Yes	2.11	0.04829	No	-	-
**DDB_G0267930**	DDB_G0267930	-	3.35	Yes	2.11	0.03377	No	-	-
**DDB_G0293102**	DDB_G0293102	-	3.01	Yes	2.18	0.00607	No	-	-
**DDB_G0275521**	DDB_G0275521	-	3.15	Yes	2.19	0.03173	No	-	-
**DDB_G0284637**	DDB_G0284637	-	7.01	Yes	2.21	0.01412	No	-	-
**DDB_G0282467**	DDB_G0282467	1-aminocyclopropane-1-carboxylate synthase	2.00	Yes	2.21	0.03377	No	-	-
**DDB_G0282715**	DDB_G0282715	Neutral and basic amino acid transport protein rBAT	1.97	Yes	2.22	0.00839	No	-	-

**DDB_G0279721**	ubqH	ubiquitin H	4.98	Yes	2.24	0.01300	No	-	-
**DDB_G0268006**	DDB_G0268006	UspA domain-containing protein	3.70	Yes	2.24	0.03164	No	-	-
**DDB_G0272280**	DDB_G0272280	AhpC/TSA family protein	8.87	Yes	2.31	0.01143	No	-	-
**DDB_G0269482**	DDB_G0269482	type A von Willebrand factor (VWFA) domain-containing protein	3.03	Yes	2.33	0.03529	No	-	-
**DDB_G0270636**	DDB_G0270636	transmembrane protein	2.02	Yes	2.34	0.01521	No	-	-
**DDB_G0292696**	colA	colossin A	2.90	Yes	2.35	0.00387	No	-	-
**DDB_G0275145**	DDB_G0275145	RING zinc finger-containing protein IBR zinc finger-containing protein	1.79	Yes	2.39	0.01515	No	-	-
**DDB_G0290957**	cprA	cysteine proteinase 1	2.11	Yes	2.47	0.00607	No	-	-
**DDB_G0280461**	DDB_G0280461	LISK family protein kinase protein kinase, TKL group tyrosine kinase-like protein	2.32	Yes	2.48	0.04814	No	-	-
**DDB_G0290959**	chtC	cheater C	2.18	Yes	2.49	0.03337	No	-	-
**DDB_G0274369**	DDB_G0274369	-	1.33	Yes	2.50	0.02777	No	-	-
**DDB_G0280051**	DDB_G0280051	-	1.79	Yes	2.50	0.00087	No	-	-
**DDB_G0275661**	DDB_G0275661	-	2.14	Yes	2.50	0.01381	No	-	-
**DDB_G0267238**	DDB_G0267238_RTE	DIRS1 ORF1	3.19	Yes	2.52	0.04872	No	-	-
**DDB_G0271134**	celA	cellulase 270-6	5.70	Yes	2.54	0.00133	No	-	-
**DDB_G0274291**	lyT2-4	putative T4-like lysozyme 2	2.51	Yes	2.58	0.00169	No	-	-
**DDB_G0283911**	hsp69	heat shock protein 69	2.40	Yes	2.67	0.02139	No	-	-
**DDB_G0279247**	DDB_G0279247	cyclin-like protein	4.50	Yes	2.70	0.04671	No	-	-
**DDB_G0293542**	DDB_G0293542	-	2.96	Yes	2.72	0.00861	No	-	-
**DDB_G0275487**	DDB_G0275487	-	4.02	Yes	2.72	0.04253	No	-	-
**DDB_G0271218**	DDB_G0271218	-	5.47	Yes	2.75	0.03172	No	-	-
**DDB_G0269112**	celB	cellulose-binding protein	1.74	Yes	2.76	0.00104	No	-	-
**DDB_G0276697**	DDB_G0276697	-	2.37	Yes	2.82	0.00355	No	-	-
**DDB_G0285917**	DDB_G0285917	-	2.21	Yes	2.84	0.00559	No	-	-
**DDB_G0285289**	spoB	spore-specific protein B	7.62	Yes	2.84	0.00387	No	-	-
**DDB_G0289473**	DDB_G0289473	P-type ATPase	1.52	Yes	2.87	0.00018	No	-	-
**DDB_G0290637**	DDB_G0290637	-	3.24	Yes	2.92	0.03449	No	-	-
**DDB_G0275133**	DDB_G0275133	-	3.77	Yes	2.94	0.02467	No	-	-
**DDB_G0289411**	DDB_G0289411	-	2.03	Yes	2.98	0.00030	No	-	-
**DDB_G0280919**	DDB_G0280919	-	2.60	Yes	3.02	0.00004	No	-	-
**DDB_G0289039**	DDB_G0289039	trypsin-like serine proteases family protein sterile alpha motif homology domain-containing protein	4.23	Yes	3.12	0.00271	No	-	-
**DDB_G0293364**	sigB	peptidase M8, leishmanolysin family protein	3.42	Yes	3.12	0.00001	No	-	-
**DDB_G0268318**	DDB_G0268318	-	3.24	Yes	3.12	0.00131	No	-	-
**DDB_G0289787**	DDB_G0289787	-	2.11	Yes	3.13	0.00384	No	-	-
**DDB_G0282059**	DDB_G0282059	-	3.91	Yes	3.18	0.00490	No	-	-
**DDB_G0285833**	DDB_G0285833	-	3.82	Yes	3.19	0.03581	No	-	-
**DDB_G0291646**	DDB_G0291646	-	2.96	Yes	3.20	0.00227	No	-	-
**DDB_G0281087**	gtaV	putative GATA-binding transcription factor GATA zinc finger domain-containing protein 22	1.86	Yes	3.25	0.00103	No	-	-
**DDB_G0288709**	tgrM2	IPT/TIG domain-containing protein immunoglobulin E-set domain-containing protein	3.55	Yes	3.28	0.00339	No	-	-
**DDB_G0292996**	rasD	Ras GTPase RasD	3.18	Yes	3.31	0.02899	No	-	-
**DDB_G0279483**	pldB	phospholipase D1	9.56	Yes	3.33	0.00030	No	-	-
**DDB_G0291223**	DDB_G0291223_RTE	DIRS1 ORF2/ORF3 fusion fragment	3.38	Yes	3.34	0.00715	No	-	-
**DDB_G0272506**	DDB_G0272506	-	2.92	Yes	3.37	0.02919	No	-	-
**DDB_G0267258**	DDB_G0267258_RTE	DIRS1 ORF2 fragment	3.00	Yes	3.43	0.00037	No	-	-
**DDB_G0287581**	DDB_G0287581	-	1.95	Yes	3.47	0.00000	No	-	-
**DDB_G0284345**	CYP556A1	cytochrome P450 family protein	2.78	Yes	3.48	0.00253	No	-	-
**DDB_G0295769**	DDB_G0295769	-	3.72	Yes	3.52	0.00516	No	-	-
**DDB_G0287311**	DDB_G0287311_ps	-	5.53	Yes	3.55	0.02467	No	-	-
**DDB_G0291420**	DDB_G0291420	-	4.53	Yes	3.63	0.00829	No	-	-
**DDB_G0281607**	DDB_G0281607	-	5.53	Yes	3.67	0.00559	No	-	-
**DDB_G0271314**	iliH	Endo-1,4-beta-glucanase family protein glycoside hydrolase family 9 protein	9.21	Yes	3.69	0.00087	No	-	-
**DDB_G0271068**	iliC	armadillo-type fold-containing protein	6.52	Yes	3.73	0.00000	No	-	-
**DDB_G0288791**	hlcs1	biotin--[acetyl-CoA-carboxylase] ligase 1	1.83	Yes	3.77	0.04403	No	-	-
**DDB_G0275197**	CYP518B1	cytochrome P450 family protein	10.47	Yes	3.78	0.01385	No	-	-
**DDB_G0274391**	alfA	alpha-L-fucosidase	2.60	Yes	3.78	0.00607	No	-	-
**DDB_G0274707**	gpt5	Putative glycophosphotransferase	7.72	Yes	3.91	0.01914	No	-	-
**DDB_G0278231**	DDB_G0278231	-	4.90	Yes	3.93	0.00559	No	-	-
**DDB_G0269254**	sigJ	transmembrane protein SigJ	5.70	Yes	3.97	0.00000	No	-	-
**DDB_G0285103**	DDB_G0285103	-	2.23	Yes	4.16	0.00000	No	-	-

Journal of Cell Science • Supplementary information

**DDB_G0276729**	DDB_G0276729	-	3.39	Yes	4.23	0.00014	No	-	-
**DDB_G0289693**	DDB_G0289693	-	2.40	Yes	4.25	0.00039	No	-	-
**DDB_G0291422**	DDB_G0291422	-	4.87	Yes	4.38	0.00087	No	-	-
**DDB_G0272214**	DDB_G0272214	-	1.65	Yes	4.62	0.01192	No	-	-
**DDB_G0283867**	cprC	cysteine proteinase 3	2.02	Yes	4.81	0.00000	No	-	-
**DDB_G0280761**	DDB_G0280761	putative sodium-dependent phosphate transporter	3.82	Yes	5.09	0.04002	No	-	-
**DDB_G0284843**	DDB_G0284843	transmembrane protein	2.66	Yes	5.22	0.00000	No	-	-
**DDB_G0276407**	DDB_G0276407	adenylate kinase	4.64	Yes	5.68	0.00000	No	-	-
**DDB_G0287037**	DDB_G0287037	Protein SDS23	5.11	Yes	6.06	0.01683	No	-	-
**DDB_G0280915**	DDB_G0280915	-	9.16	Yes	7.49	0.00317	No	-	-
**DDB_G0283483**	srfD	putative MADS-box transcription factor	2.86	No	-	-	Yes	2.45	0.037579
**DDB_G0270540**	DDB_G0270540	-	3.51	No	-	-	Yes	2.77	0.037595
**DDB_G0287583**	gerC	spore germination protein C	7.80	No	-	-	Yes	3.13	0.037595
**DDB_G0278953**	DDB_G0278953	CBS (cystathionine-beta-synthase) domain-containing protein	5.50	No	-	-	Yes	3.13	0.007318
**DDB_G0286239**	DDB_G0286239	alpha/beta hydrolase fold-1 domain-containing protein serine hydrolase-like protein	2.63	No	-	-	Yes	3.14	0.010326
**DDB_G0289723**	abhd	alpha/beta hydrolase fold-1 domain-containing protein abhydrolase domain-containing protein	2.14	No	-	-	Yes	3.49	0.005321
**DDB_G0284511**	DDB_G0284511	-	3.79	No	-	-	Yes	3.51	0.017282
**DDB_G0283281**	DDB_G0283281	Strictosidine synthase family protein	5.58	No	-	-	Yes	3.59	0.007318
**DDB_G0283649**	DDB_G0283649	NADPH-dependent FMN reductase family protein putative FMN-dependent NAD(P)H:quinone reductase	2.39	No	-	-	Yes	3.72	0.037595
**DDB_G0268160**	gnt12	putative glycosyltransferase putative GlcNAc transferase putative beta-1,3 N-acetylglucosaminyltransferase	3.06	No	-	-	Yes	4.23	0.031858
**DDB_G0282353**	CYP513E1	cytochrome P450 family protein	6.23	No	-	-	Yes	4.44	0.038561
**DDB_G0283213**	DDB_G0283213	-	6.44	No	-	-	Yes	5.77	0.000208
**DDB_G0281361**	DDB_G0281361	-	2.75	No	-	-	Yes	6.48	0.000163

Journal of Cell Science • Supplementary information

Table S6. Summary of rnp1A knockdown and overexpression phenotypes.

Phenotypes	*rnp1A* knockdown	RNP1A overexpression
Growth Rate	↓	↓
Adhesion	↓	No Change
Cytokinesis Defect	✓	No Change
Cortical Tension	↓	↑
Microtubule Contact Number	↓	N/A
Microtubule Contact Length	↓	N/A
Total Nutrient Uptake via TRITC-Dextran	↓↓	↓
DQ-Red BSA Uptake	↓↓	↓
Macropinocytosis Frequency	↓	N/A
TRITC-Dextran Degradation Speed	↓	N/A

Table S7. A summary of strains used in this study.

**Strain name**	**Strain genotype**	**Experimental applications**
WT	Ax3 (Rep orf+)::pLD1A15SN	Cell growth; Adhesion; Cytokinesis; RNA-seq; qRT-PCR; western blot; macropinocytosis
WT (doxycycline induction)	Ax3 (Rep orf+)::pDM310	qRT-PCR; western blot; Macropinocytosis
WT::GFP	Ax3 (Rep orf+)::GFP:pLD1A15SN	Live cell imaging; CLIP-seq; macropinocytosis
WT::mCh	Ax3 (Rep orf+)::mCherry:pDRH; pLD1A15SN	FCS
WT::GFP-RNP1A	Ax3 (Rep orf+)::GFP-RNP1A:pLD1A15SN	Live cell imaging; CLIP-seq; macropinocytosis; gel compression
WT::mCh-Cortexillin I	Ax3 (Rep orf+)::mCherry-Cortexillin I:pDRH; pLD1A15SN	FCS
WT::GFP-tubulin	Ax3 (Rep orf+)::GFP-alpha tubulin:pDRH; pLD1A15SN	TIRF
WT::GFP-DlpA	Ax3 (Rep orf+)::GFP-DlpA:pSAD	Macropinocytosis
WT::GFP-RNP1A;RFP-limEΔcoil	Ax3 (Rep orf+)::GFP-RNP1A:pLD1A15SN; RFP-limEΔcoil:pDRH	Macropinocytosis
WT::GFP-RNP1A	KAx3::GFP-RNP1A:pLD1A15SN; pREP	Macropinocytosis
WT::GFP-mCh linked	KAx3::GFP-5AA-mCherry:pDM181; FLAG:pDRH	FCCS
WT::GFP; mCh unlinked	KAx3::GFP:pDM181; mCherry:pDRH	FCCS
WT::GFP; mCherry	Ax3 (Rep orf+)::GFP: pLD1A15SN; mCherry:pDRH	Live cell imaging and random migration
WT::GFP-RNP1A; mCherry	Ax3 (Rep orf+)::GFP-RNP1A: pLD1A15SN; mCherry:pDRH	Live cell imaging and random migration
WT (*dlpA* null parental)	Ax2	Macropinocytosis
dlpA null	*dlpA-*	Macropinocytosis
Doxycycline-inducible *rnp1A* hp	Ax3 (Rep orf+)::*rnp1A* hp:pDM310	qRT-PCR; western blot; macropinocytosis
rnp1A hp	Ax3 (Rep orf+)::*rnp1A* hp:pLD1A15SN	Cell growth; Adhesion; Cytokinesis; RNA-seq; qRT-PCR; western blot; macropinocytosis
rnp1A hp; mCh	Ax3 (Rep orf+)::mCherry:pDRH; *rnp1A* hp:pLD1A15SN	FCS
rnp1A hp; mCh-Cortexillin I	Ax3 (Rep orf+)::mCherry-Cortexillin I:pDRH; *rnp1A* hp:pLD1A15SN	FCS
rnp1A hp; GFP-tubulin	Ax3 (Rep orf+)::GFP-alpha tubulin:pDRH; *rnp1A* hp:pLD1A15SN	TIRF
RNP1A OE	Ax3 (Rep orf+)::RNP1A:pLD1A15SN	Cell growth; Adhesion; Cytokinesis; qRT-PCR; western blot; macropinocytosis
myoII::GFP	*mhcA*::GFP:pDM181	Macropinocytosis
myoII::GFP-Myosin II	*mhcA*::GFP-Myosin II:pBIG	Macropinocytosis
myoII::GFP-RNP1A	*mhcA*::GFP-RNP1A:pLD1A15SN; pREP	Macropinocytosis
myoII::GFP-DlpA	*mhcA*::GFP-DlpA:pSAD	Macropinocytosis
myoII::3xAla	*mhcA*::GFP-3xAla:pBIG	Macropinocytosis
myoII::3xAsp	*mhcA*::GFP-3xAsp:pBIG	Macropinocytosis
myoII::GFP-Myosin II; RFP-limEΔcoil	*mhcA*::GFP-Myosin II:pBIG; RFP-limEΔcoil:pDRH	Macropinocytosis
cortI::GFP-Cortexillin I	*cortexillin I* (KAx3)::GFP-Cortexillin I:pExp4	Macropinocytosis
cortI::GFP-RNP1A	*cortexillin I*(KAx3)::GFP-RNP1A:pLD1A15SN; pREP	Macropinocytosis
cortI::GFP-DlpA	*cortexillin I* (KAx3)::GFP-DlpA:pSAD	Macropinocytosis
cortI::GFP-Cortexillin I; RFP-limEΔcoil	*cortexillin I* (KAx3)::GFP-Cortexillin I:pExp4; RFP-limEΔcoil:pDRH	Macropinocytosis
iqg1::GFP	*iqgap1* (KAx3)::GFP:pExp4	Macropinocytosis
iqg1::GFP-IQGAP1	*iqgap1* (KAx3)::GFP-IQGAP1:pExp4	Macropinocytosis
iqg1::GFP-RNP1A	*iqgap1* (KAx3)::GFP-RNP1A pLD1A15SN; pREP	Macropinocytosis
iqg1::GFP-DlpA	*iqgap1* (KAx3)::GFP-DlpA:pSAD	Macropinocytosis
iqg1::GFP-RNP1A;mCh-IQGAP1	*iqgap1* (KAx3)::mCherry-IQGAP1:pDRH; GFP-RNP1A:pLD1A15SN; pREP	FCCS
iqg2::GFP-IQGAP2	*iqgap2* (KAx3)::FLAG-GFP-IQGAP2:pDM181	Macropinocytosis
iqg2::GFP-RNP1A	*iqgap2* (KAx3)::GFP-RNP1A:pLD1A15SN; pREP	Macropinocytosis
iqg2::GFP-RNP1A; mCh-IQGAP2	*iqgap2* (KAx3)::GFP-RNP1A:pLD1A15SN; pREP; mcherry-IQGAP2:pDRH	FCCS
iqg1::GFP-IQGAP1; RFP-limEΔcoil	*iqgap1* (KAx3)::GFP-IQGAP1:pExp4; RFP-limEΔcoil:pDRH	Macropinocytosis

Journal of Cell Science • Supplementary information

Table S8. List of antibodies used in this study.

Protein	Species/Chemical	Dilution Factor	Source or reference	Catalogue number
Cortexillin I	mouse	1000	Developmental Studies Hybridoma Bank	241-438-1
alpha Tubulin	mouse	1000	Developmental Studies Hybridoma Bank	12G10
Actin	phalloidin-647	400	Developmental Studies Hybridoma Bank	224-236-1
F-actin	Rhodamine-phalloidin	400	Sigma-Aldrich	P1951
Discoidin 1A	mouse	1000	Developmental Studies Hybridoma Bank	80-52-13
Dynacortin	rabbit	50000	Robinson & Spudich, 2000	-
Myosin II heavy chain	mouse	1000	Developmental Studies Hybridoma Bank	56-396-5
RNP1A	rabbit	2000	AbClonal	-

Table S9. List of primers used in this study.

Primer name	Sequence
myoII 3' FWD	GCCGATAAGAGTAAGAAGAC
myoII 3' REV	GGATTGTTCAGCTTCAGATT
act8/11 5' FWD	ATATGTAGGTGATGAAGCCC
act8/11 5' REV	GACGTACATGGCTGGGGTGTT
tubA 5' FWD	AGAAAGTTGTCCCAAGAGCC
tubA 5' REV	CGTCTGATACGATCGACGCA
rnp1A 5' FWD	CGACGGTAAACAAGTCAATG
rpn1A 5' REV	GTAGCATTTTCGCCAGTGG
abcF4 3' FWD	GAGCGAGAGGATGCTTCACT
abcF4 3' REV	GAGAGAATCTAGCTACACGC
rpl13 5' FWD	CGACCATTTCCGTAAACACTG
rpl13 5' REV	CTAAGGTGAAACCACGACCA
cadA 5' FWD	GATGTAACATTTGGGAGCAT
cadA 5' REV	GCTCCTGGTAAGACTTGGAA
rps3a 5' FWD	TCGACCCATTCACCCGTAAG
rps3a 5' REV	GTTTGGTACCTTGGGTTCTG
discoidin 1A FWD	ATGTCTACCCAAGGTTTAGT
discoidin 1A REV	CACAACCAGCAACAATGTAT
cortexillin I 3' FWD	GGCTCGTATGGAACTCAGAG
cortexillin I 3' REV	AAGAGACCAAGACCTTGAGC

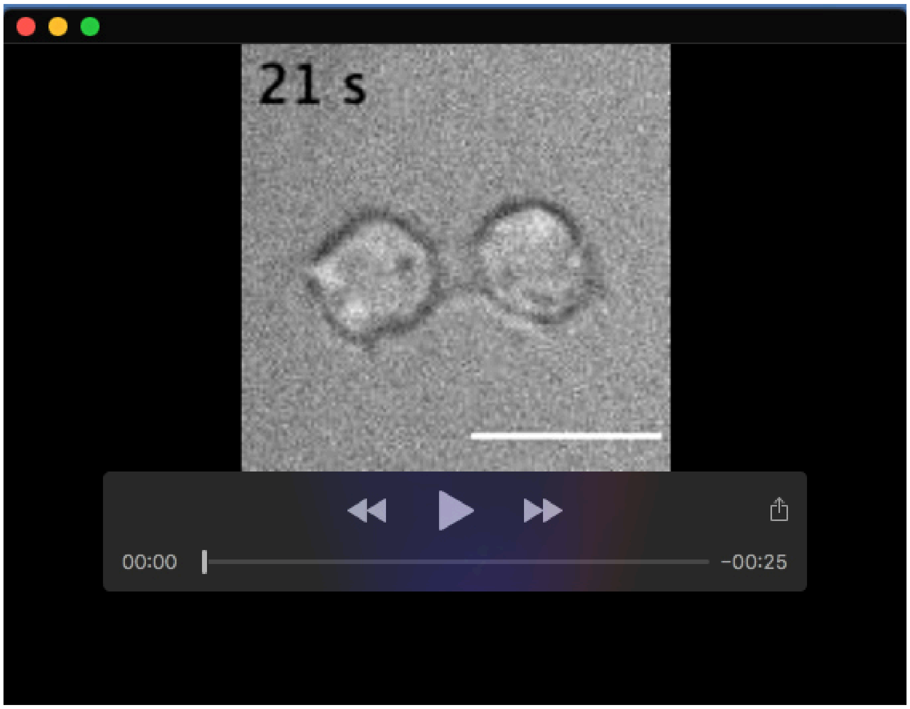

Movie 1. Cytokinesis progression of a wild type control cell. Scale bar, 10 μm.

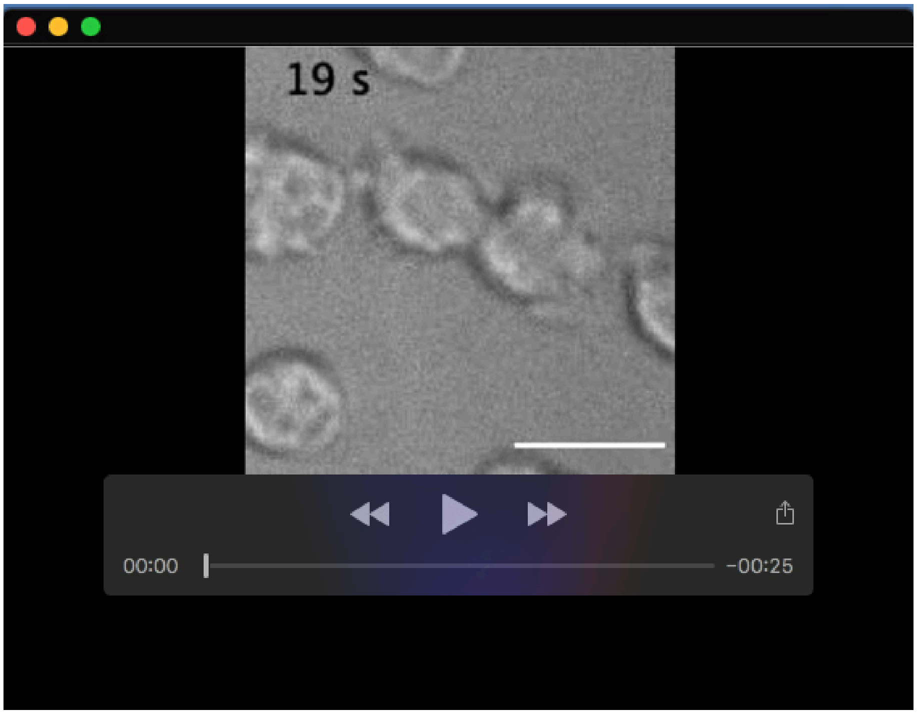

Movie 2. Cytokinesis progression of an *rnp1A* knockdown cell. Scale bar, 10 μm.

Journal of Cell Science • Supplementary information

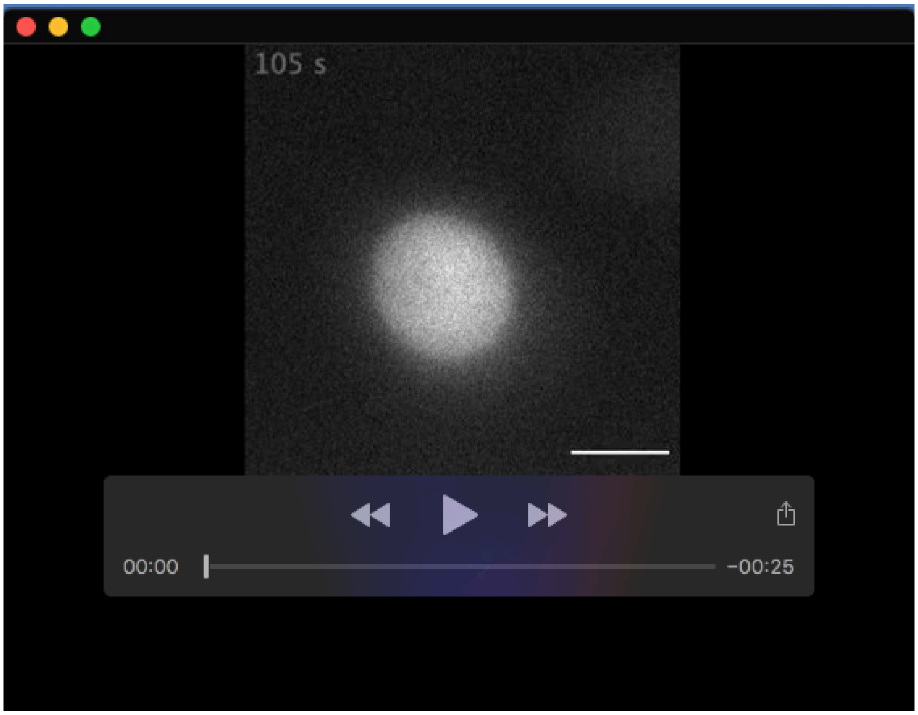

Movie 3. Random migration of a wild type cell, expressing GFP. Scale bar, 10 µm.

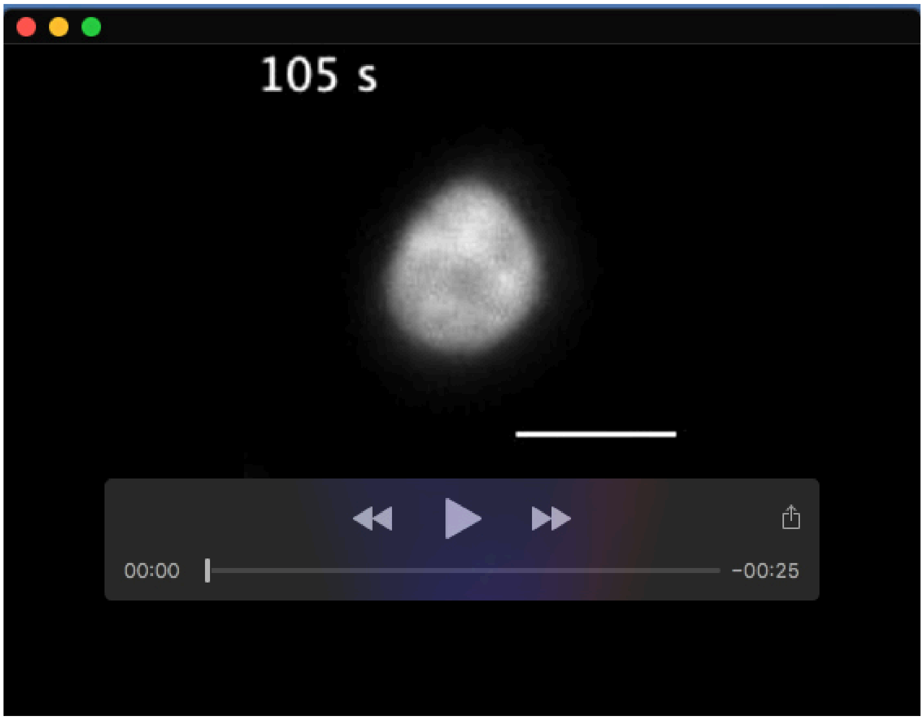

Movie 4. Random migration of a wild type cell, expressing GFP-RNP1A. Scale bar, 10 µm.

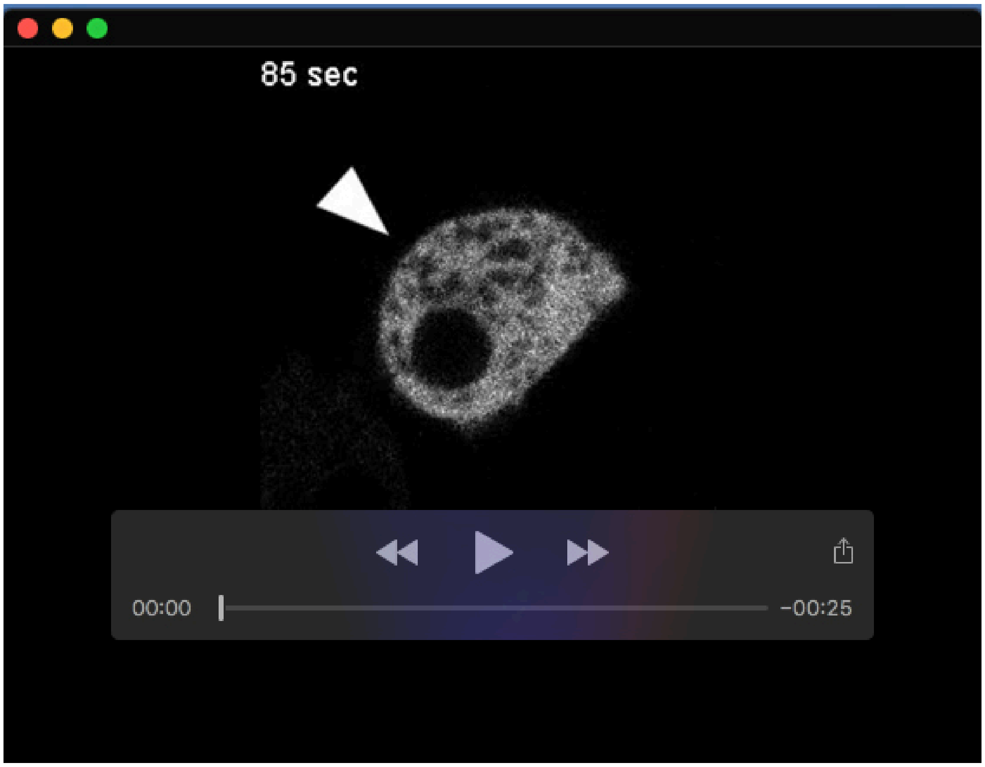

Movie 5. Macropinocytotic crown formation and closure of a wild type cell, expressing GFP. Cascade Blue Dextran was used for macropinocytosis. Scale bar, 10 µm.

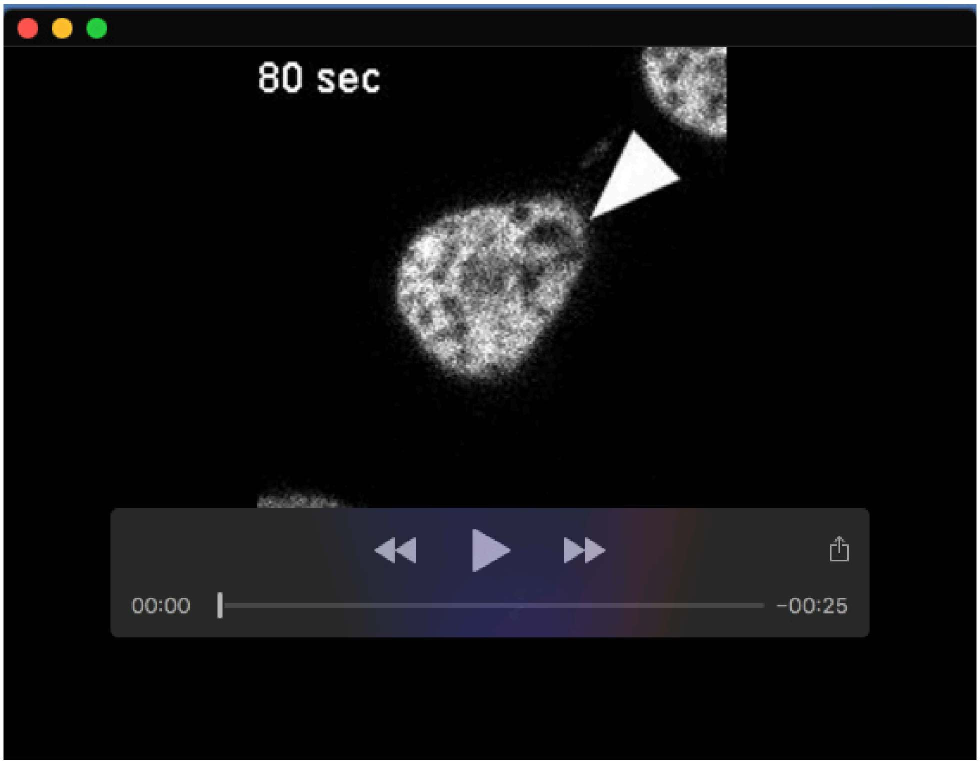

Movie 6. Macropinocytotic crown formation and closure of a wild type cell, expressing GFP-RNP1A. Cascade Blue Dextran was used for macropinocytosis. Scale bar, 10 µm.

Journal of Cell Science • Supplementary information

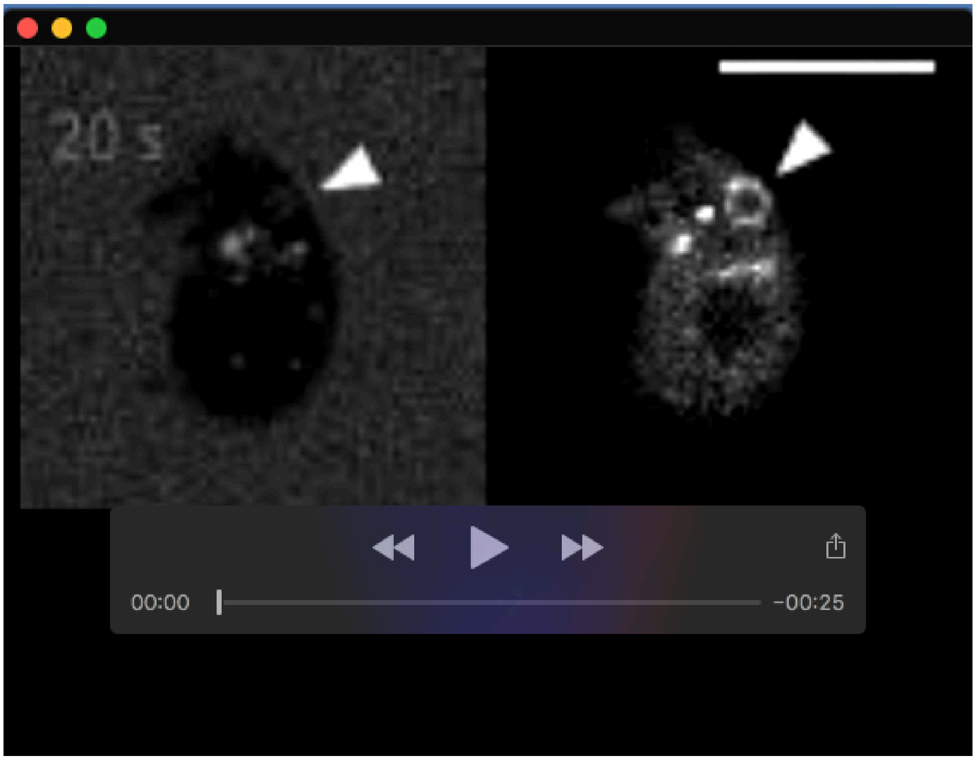

Movie 7. Macropinocytotic crown formation and closure of a wild type cell, expressing GFP-DlpA. Left: TRITC-Dextran; Right: GFP-DlpA. Scale bar, 10 µm.

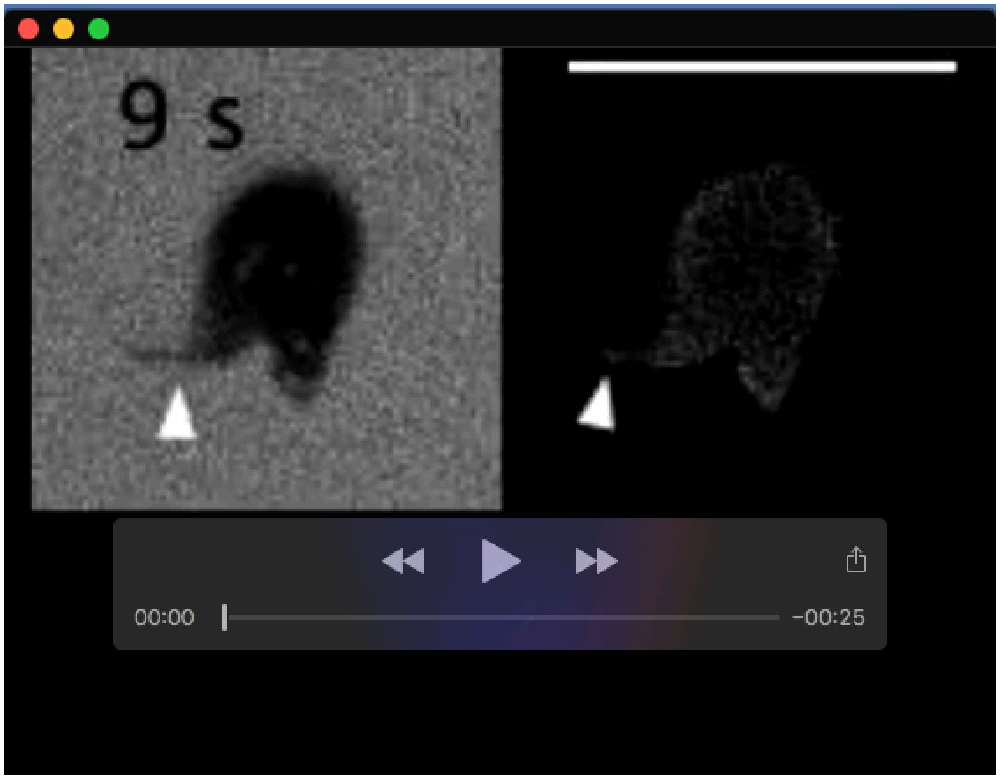

Movie 8. Macropinocytotic crown formation and closure of an *iqgap1* null cell, expressing GFP-IQGAP1. Left: TRITC-Dextran; Right: GFP-IQGAP1. Scale bar, 10 µm.

Journal of Cell Science • Supplementary information

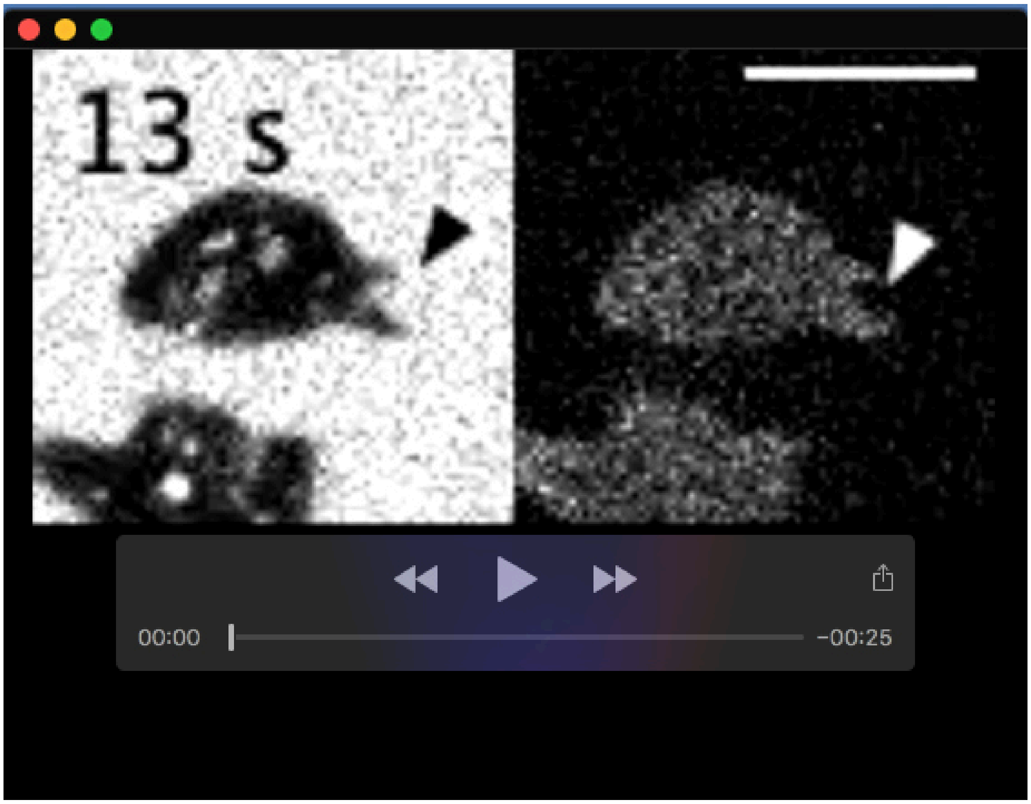

Movie 9. Macropinocytotic crown formation and closure of an *iqgap2* null cell, expressing GFP-IQGAP2. Left: TRITC-Dextran; Right: GFP-IQGAP2. Scale bar, 10 µm.

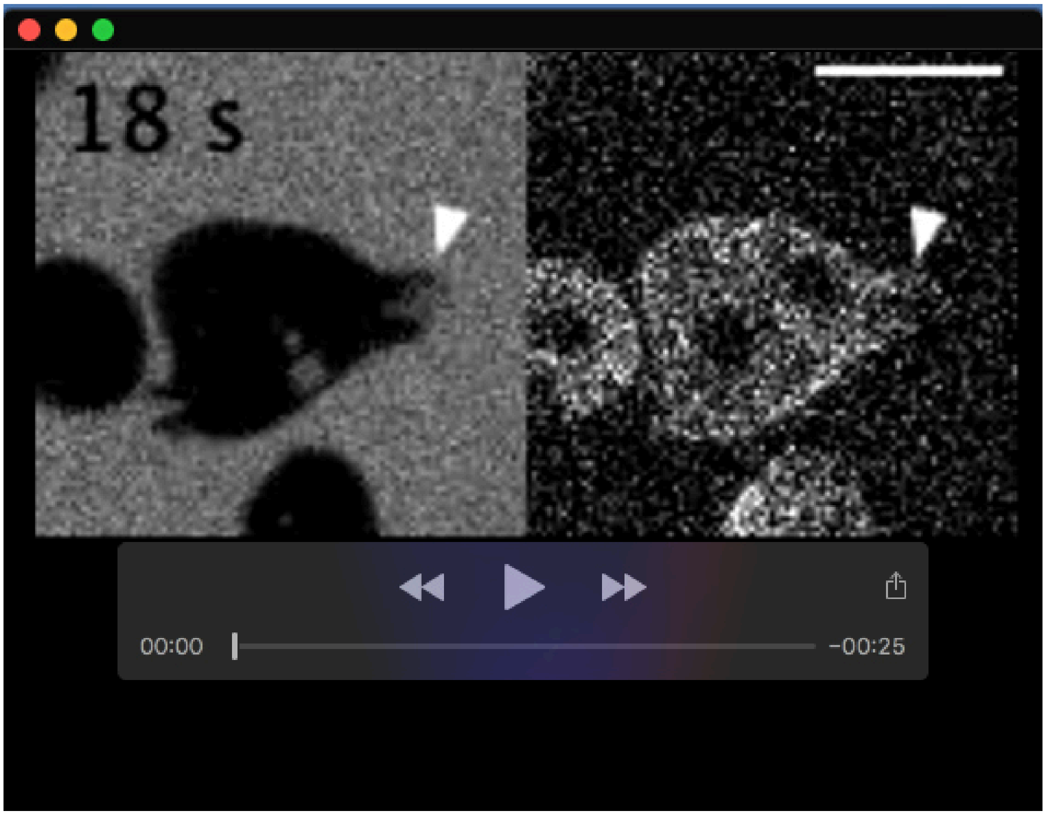

Movie 10. Macropinocytotic crown formation and closure of a *myosin II* null cell, expressing GFP-Myosin II. Left: TRITC-Dextran; Right: GFP-Myosin II. Scale bar, 10 µm.

Journal of Cell Science • Supplementary information
